# Iron Complexes Supported by a Rigid Macrocyclic PNPN Ligand

**DOI:** 10.1021/acs.inorgchem.6c02531

**Published:** 2026-08-26

**Authors:** Stefan Weber, Berthold Stöger, Maren Podewitz

**Affiliations:** † Institute of Applied Synthetic Chemistry, 27259 TU Wien, Getreidemarkt 9, A-1060 Vienna, Austria; ‡ X-Ray Center, TU Wien, Getreidemarkt 9, A-1060 Vienna, Austria; § Institute of Materials Chemistry, TU Wien, Getreidemarkt 9, A-1060 Vienna, Austria

## Abstract

Seesaw ligands enforce a coordination geometry of the two coligands being in a *cis* configuration in an octahedral environment. While nitrogen-based weak field seesaw ligands are often employed, strong field ligands with mixed nitrogen and phosphine donors are rarely encountered. Herein we report the synthesis of iron­(II) dihalide complexes **1** supported by a rigid seesaw PNPN ligand, yielding *C*_2*v*_-symmetric compounds. Halide abstraction resulted in formation of 5-fold coordinated cationic complexes **2**. Uptake of π-acidic ligands gave cationic complex **3**. Dicationic complex **4**, containing two acetonitrile monodentate ligands, was prepared from Fe­(OTf)_2_. The structure of **1** and **3** was studied in solid-state and in solution, including ^15^N NMR spectroscopy. Furthermore, the application as catalysts in sp^2^–sp^3^ Kumada cross-coupling was demonstrated, showing high reactivity with a catalyst loading of 1 mol% in 1 h reaction time at room temperature.

## Graphical abstract

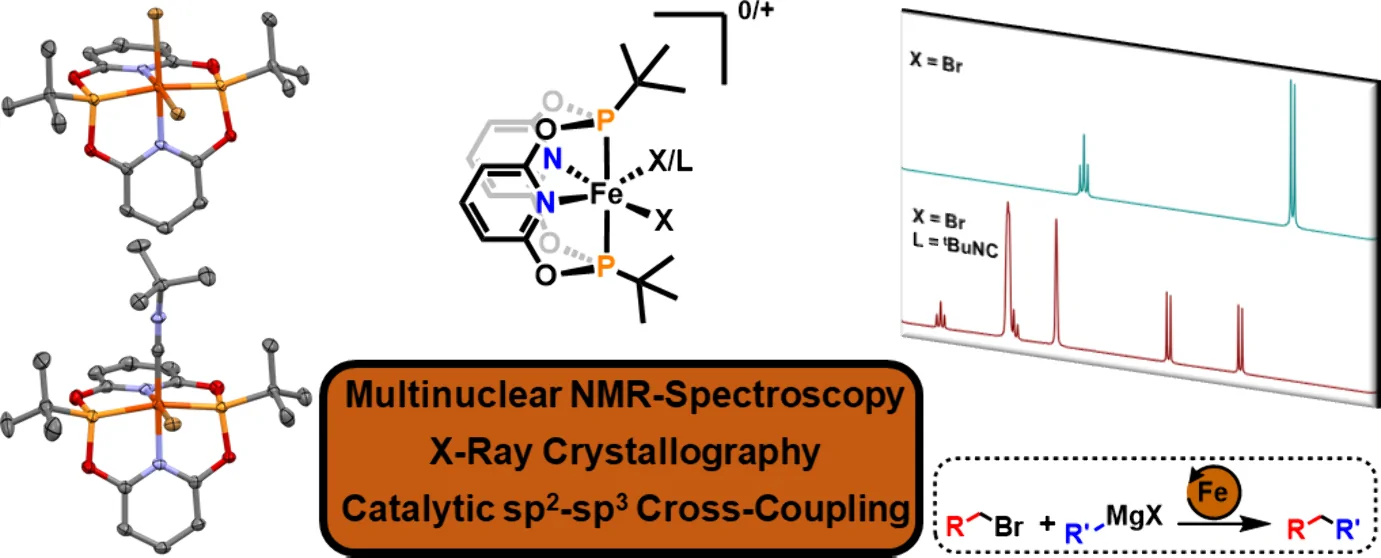

Controlling the coordination geometry and fine-tuning of the electronic structure in transition-metal complexes remain central principles to tailor their properties. Polydentate ligands are frequently employed to fulfill this task. Concerning planar tetradentate macrocyclic ligands, porphyrins^1^ and corroles^2^ are often encountered in various applications. In contrast, macrocyclic tetradentates that bind to the metal center in a seesaw fashion can also be employed. Late transition metals supported by seesaw all-nitrogen donors^3^ were recently leveraged for biological^4^ and synthetic applications.^5^ Notably, the stabilization of unusual oxidation states and their potential in bond formation reactions was impressively demonstrated.^6^

Reports on mixed nitrogen/phosphine donors are less common. Fryzuk introduced a dianionic macrocyclic ligand for the activation and derivatization of dinitrogen with zirconium^7^ and the investigation of tantalum alkyl and alkylidene complexes (Figure 1).^8^ Niecke employed a sterically demanding neutral PNPN ligand to support a copper­(I) center.^9^ Recently, the application of octahedral nickel­(II) complexes based upon a PNPN ligand was showcased by Mirica.^10^

**Figure 1:**
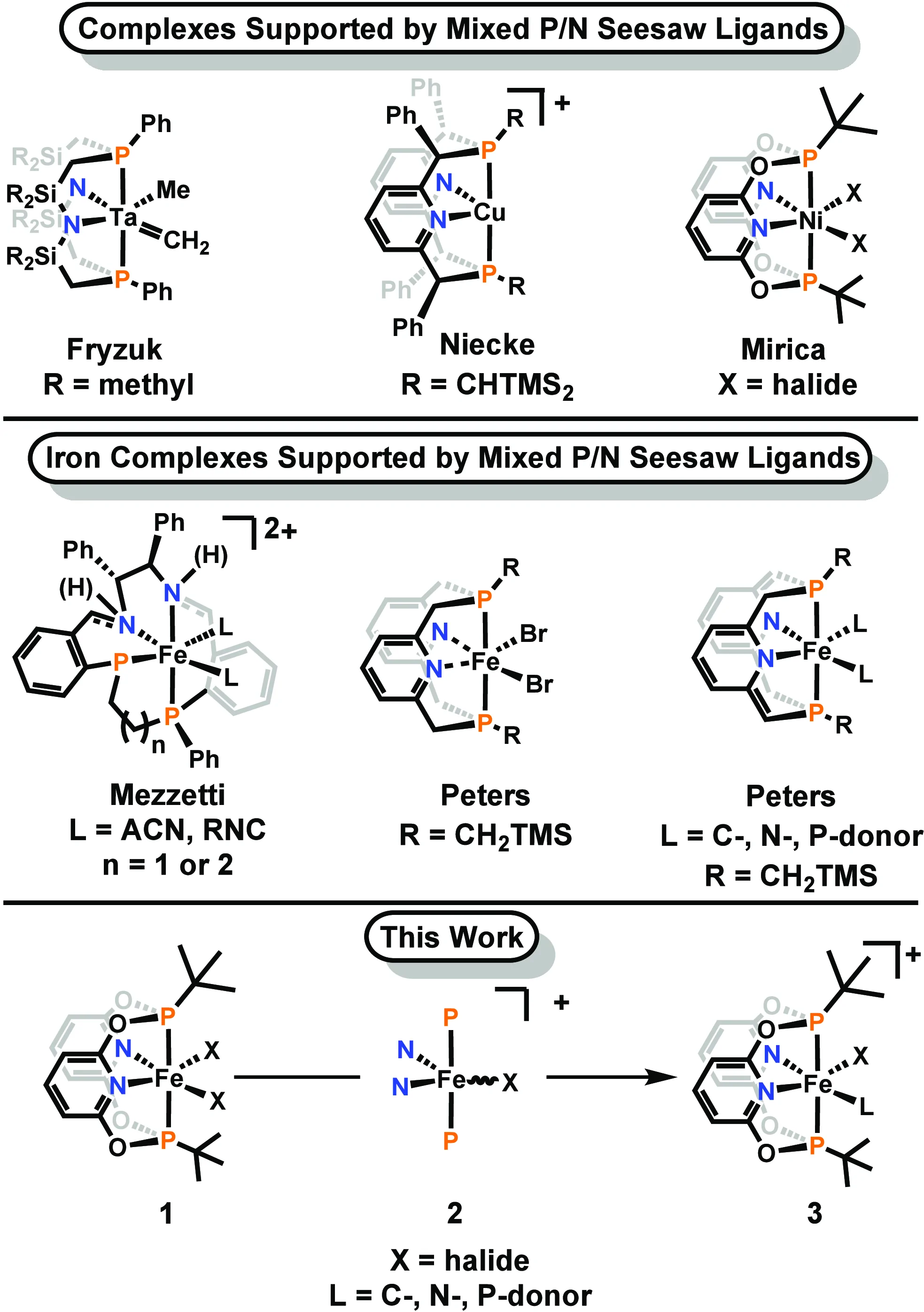
Representative examples of transition metal complexes supported by mixed nitrogen/phosphorus seesaw ligands and outline of this article.

Focusing on iron, reports on complexes supported by macrocyclic seesaw strong field P/N donors are relatively rare. Mezzetti reported iron complexes based upon PPNN ligands.^11^ However, due to the flexibility of the ligand backbone, isomerization from a seesaw to a planar coordination mode could be observed. More recently, Peters mapped the coordination chemistry of a PNPN ligand, containing C–H acidic linkers.^12^ In its neutral form, the PNPN ligand scaffold is flexible enough to bind in a seesaw mode to give an octahedral geometry or in a bidentate fashion with the phosphines binding, yielding a tetrahedral high spin complex. Dual dearomatization leads to the formation of octahedral low spin complexes upon uptake of π-accepting ligands.

Given that reports on iron complexes supported by strong-field macrocyclic seesaw ligands are still relatively scarce, we wondered how the coordination chemistry of iron­(II) complexes would change if Mirica’s rigid and synthetically more accessible PNPN ligand^10^ were employed. In this work, we describe the synthesis of a series of iron­(II) complexes supported by this PNPN ligand, characterization thereof, and their application in sp^2^–sp^3^ Kumada cross-coupling reactions.

Synthetic entrance to well-defined Fe­(II) complexes could be achieved upon reaction of the PNPN ligand with FeX_2_ (X = Cl, Br, I) to yield red complexes **1** in good yields (Scheme 1). Having **1** as defined complexes in hand, we studied the reaction with halide abstraction reagents. No reaction of **1** with the typical halide abstraction agents NaBF_4_ or NH_4_PF_6_ could be observed while the corresponding silver salts gave intractable mixtures. However, the abstraction of one equivalent of halide and formation of cationic complexes **2** was observed, when NaBArF_24_ was employed. This is in contrast to the reaction of the iron PNPN complex reported by Peters, which undergoes demetalation upon halide abstraction.^12^ Compounds **2** are paramagnetic with a μ_eff_ of 2.9 for **2-Cl**, 1.6 for **2-Br**, and 1.3 for **2-I** in dichloromethane solution at room temperature. Unfortunately, we were unable to obtain single crystals for complexes **2**; thus we turned our attention to computational modeling. Based on DFT calculations (TPSSh/def2-TZVPP/D4), **2** are best described as 5-fold coordinated *C*_2_-symmetric complexes. This is also supported by the ^1^H NMR spectroscopy for **2-Cl**, which gives assignable resonances. We found a small singlet/triplet gap, slightly favoring the triplet states over diamagnetic states (1.8–5.4 kcal/mol, for details see ). The quintet states also seem to be accessible, given that these high spin states lie only 4.9–8.5 kcal/mol above the triplet states. Thus, we propose that a population of multiple spin states for complexes **2** is viable. This is in line with optical spectroscopy and computational modeling thereof, which indicate that the resulting spectra may be mixtures with signatures of individual spin states (for details see ). Additional halide abstraction from **2** (>5 equiv of NaBArF_24_) was not observed.

**Scheme 1:**
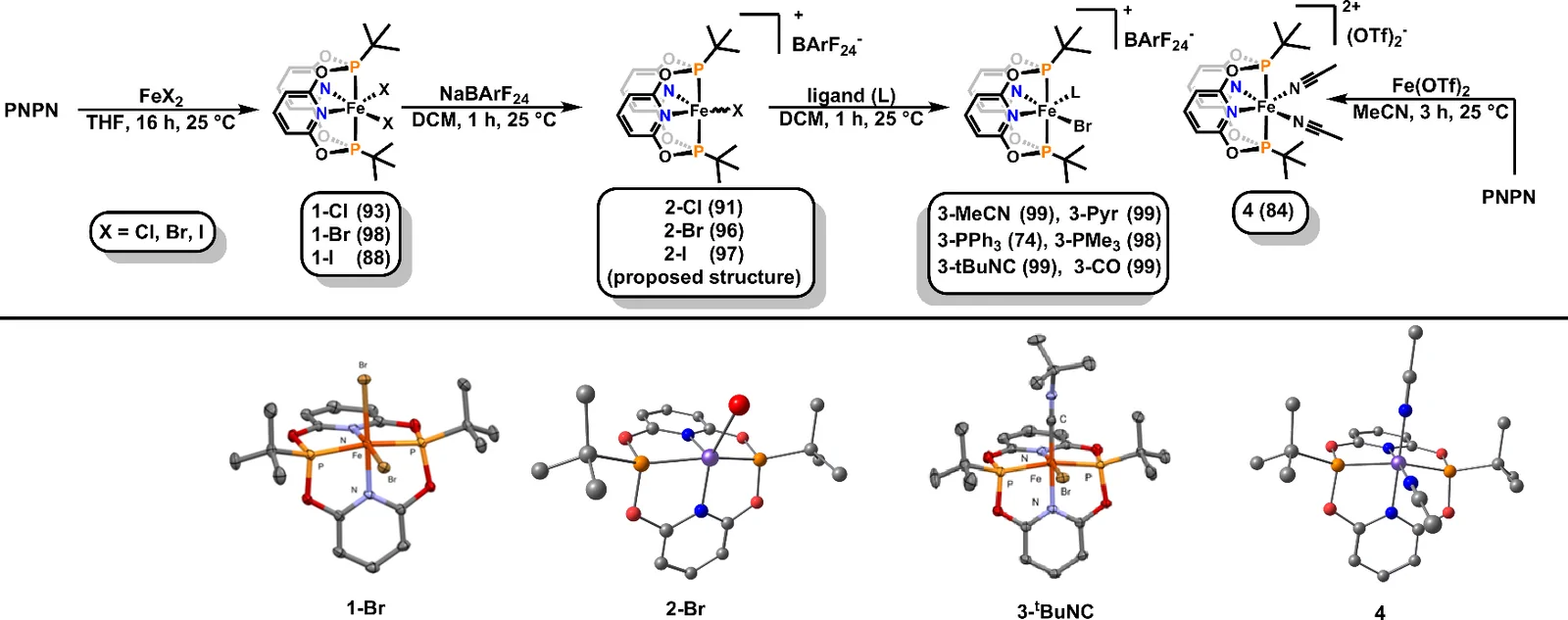
Synthesis of Iron­(II) Complexes **1**–**4** Supported by PNPN Ligand (top), Solid State Structure of **1-Br** (bottom left) and **3-tBuNC** (bottom, second from right),^a^ and DFT Optimized Structures of **2-Br** in Triplet State (bottom, second from left) and **4** in Singlet State (bottom right)^b^

a Thermal ellipsoids are set at 50% probability; hydrogen atoms and counter ion (for **3-tBuNC**) are omitted for clarity.
b DFT calculations employed TPSSh,^13^ def2-TZVPP^14^ basis set, and D4 correction.^15^

Complexes **2** readily react with stoichiometric amounts of acetonitrile, yielding monomeric complexes, which is consistent with a coordinatively unsaturated structure in solution. Under the same reaction conditions, complexes **1** are inert to acetonitrile. In order to explore the coordination chemistry of **2-Br**, binding of various ligands to the Fe-center was tested to give diamagnetic complexes **3**. We found that π-donating ligands, such as alcohols or ethers do not bind to the metal center. However, a broad range of π-acceptors show high affinity to the Fe-center. Nitrogen-, phosphorus-, and carbon-based donors were found to stabilize the cationic complexes in a singlet state. Again, no halide abstraction from **3** was observed in the presence of monodentate compound (e.g., MeCN) and NaBArF_24_. However, we were able to prepare the dicationic complex **4** by utilizing Fe­(OTf)_2_ as iron precursor in acetonitrile as solvent.

Concerning the structure of complexes in the solid state, **1-Br** adopts a slightly distorted octahedral geometry with the two bromide ligands in *cis* configuration. Likewise, **3-tBuNC** shows a slightly distorted octahedral geometry. The Fe–N bond lengths for the pyridine donor *trans* to the bromide ligand in **1-Br** and **3-tBuNC** do not differ, while a slightly longer Fe–N bond for the pyridine donor *trans* to the tBuNC ligand can be observed for **3-tBuNC** (Table 1). A minor contraction of the Fe–Br bond in **3-tBuNC** compared with **1-Br** can be observed, as expected for a cationic complex versus a neutral congener. The N–Fe–N bond angle is slightly affected by ligand substitution, while a decrease of 1.5° of the P–Fe–P bond angle is observed from neutral **1-Br** to cationic **3-tBuNC**. Most prominent is the decrease of the bond angle for the two monodentate ligands form 93.5° in **1-Br** to 85.6° in **3-tBuNC**.

**Table 1:** Selected Structural Parameters of **1-Br** and **3-tBuNC** as Detected by Single Crystal Analysis

	**1-Br**	**3-tBuNC**
Bond Length [Å]^a^		
N(*trans* Br)–Fe	1.950(2)	1.950(2)
N(*trans* tBuNC)–Fe	-	1.980(2)
P–Fe	2.140(9)	2.152(7)
Br–Fe	2.430(7)	2.440(8)
Bond angles [deg]		
N–Fe–N	89.5(9)	89.9(1)
P–Fe–P	158.8(1)	157.3(6)
Br–Fe–Br	93.5(8)	-
Br–Fe–C	-	85.6(8)

a Averaged within one molecule.

Furthermore, the structures of complexes **1**, **3**, and **4** in solution were studied by multinuclear NMR spectroscopy. As expected, complexes **1** maintain *C*_2*v*_-symmetry, indicated by two resonances of the pyridine donor of the seesaw ligand (Figure 2, top). Noteworthy, Peters’ PNPN iron dibromide complex^12^ is paramagnetic presumably due to fluxional binding of the pyridine donors. We attribute the fact that complexes **1** are diamagnetic to increased rigidity of the here employed O-linked PNPN over the more flexible PNPN containing CH_2_-linkers. Upon substitution of one halide by a neutral donor in **3**, the *C*_2*v*_-symmetry is lost. Consequently, these complexes give rise to two sets of signals for the pyridine donor of the PNPN ligand in the ^1^H and ^13^C NMR spectra as expected for *C*_*s*_ symmetric compounds. The *C*_2*v*_-symmetry is restored in complex **4**.

Furthermore, we were interested in the impact of ligand substitution on the electronic structure of the pyridine donors of the macrocyclic ligands and thus the shifts in ^15^N NMR (Table 2). In the halide series **1**, we could observe an increased deshielding from chloride over bromide to iodide. By combining the data sets of **1-Br** and **4**, we were able to assign the ^15^N resonances for the respective pyridine donors of unsymmetrical complexes **3**. Within the series of **3**, the impact on ^15^N shifts of the pyridine donor *trans* to the bromide ligand is modest for phosphine- or carbon-based donors, while nitrogen-based donors lead to increased shielding of the ^15^N resonance (see details in the ). We found a reasonable correlation between the ^15^N resonance and the calculated Hirshfeld charges^16^ for the Fe (for details see ). Shielding is more prominent in the ^15^N shifts of the pyridine donor *trans* to the neutral donor. As a qualitative trend, we found increased deshielding in ^15^N shifts with an increase in donor properties of the neutral donor as described by the calculated Tolman electronic parameters, which decrease with increased donor properties.^17^

**Figure 2:**
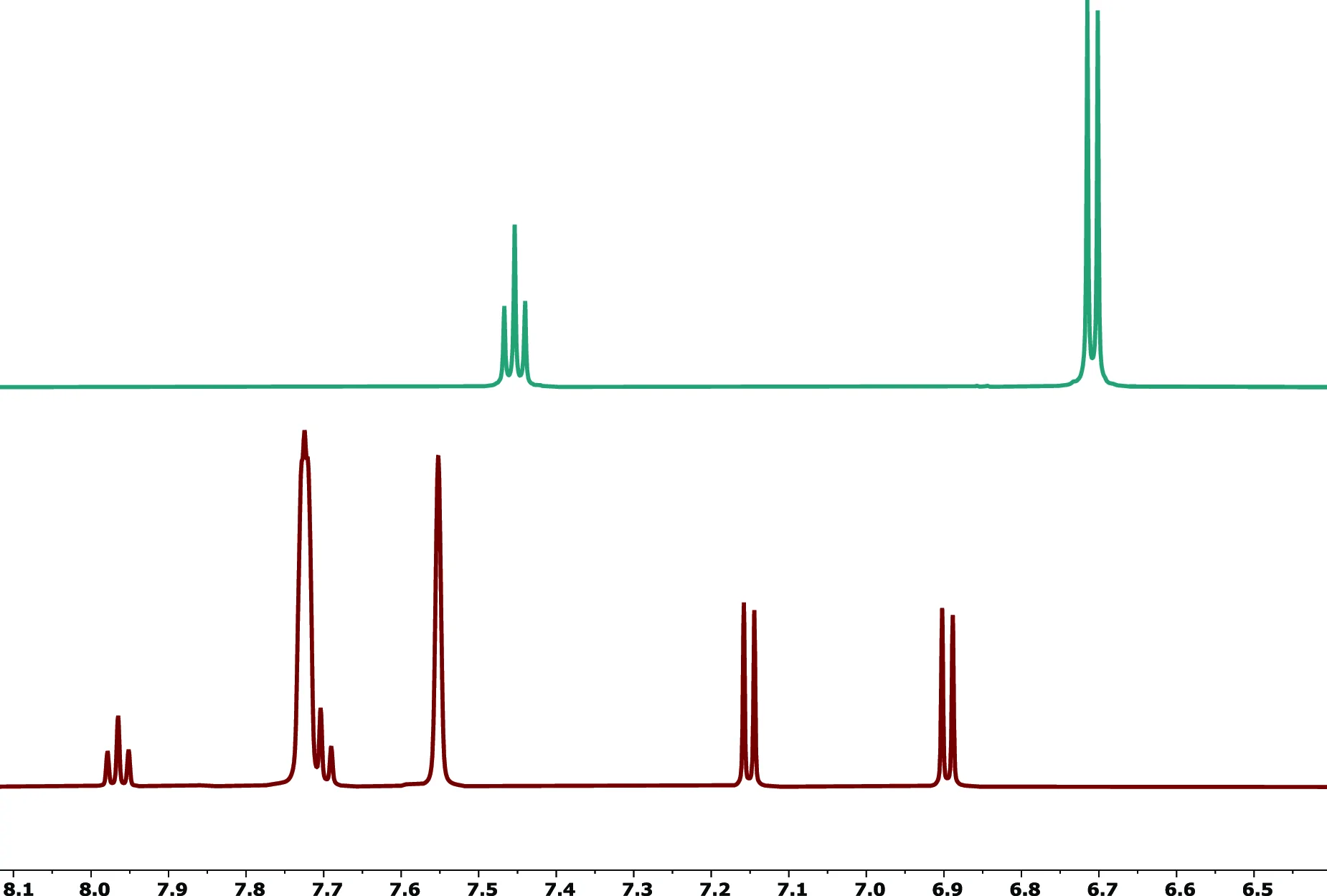
Aromatic region of ^1^H NMR (600 MHz, 25 °C) for complexes **1-Br** (top) and **3-tBuNC** (bottom, resonances at 7.72 and 7.55 ppm represent BArF_24_ counterion).

**Table 2:** ^15^N NMR Shifts and Calculated Hirshfeld Charges as well as Tolman Electronic Parameters (TEP) for Complexes **1**, **3**, and **4**

Compound	^15^N shift *trans* halide [ppm]	^15^N shift *trans* L [ppm]	Hirshfeld Charge on Fe	TEP [cm^–1^]
**1-Cl**	205.4	-	–0.065	-
**1-Br**	215.2	-	–0.089	-
**1-I**	221.7	-	–0.086	-
**3-ACN**	208.1	195.3	–0.116	2118
**3-Pyr**	202.6	199.1	–0.116	2110
**3-PPh**_**3**_	215.3	205.3	–0.113	2107
**3-PMe**_**3**_	218.2	200.5	–0.111	2105
**3-tBuNC**	215.4	204.1	–0.113	2112
**3-CO**	215.6	188.6	–0.114	2165
**4**	-	189.5	0.008	2118

At last, we examined the catalytic activity of all complexes in sp^2^–sp^3^ Kumada-type cross-coupling reactions since the seesaw ligand may stabilize low-valent iron intermediates.^18^ As a model reaction, we chose cyclohexyl bromide as an electrophile and phenyl magnesium bromide as a nucleophile (see SI, ). Employing 3 mol% iron complex resulted in good yields for complexes **1**, **2**, and **4**.

Complexes **3** were also found to be competent catalysts for this transformation (58–70% yield); however, **3-CO** and **3-tBuNC** showed substantially lower productivity.

The scope and limitations of the system were established with optimized conditions, employing **1-Cl** (1 mol%) as catalyst in Et_2_O as solvent (Table 3). Regarding electrophiles, good productivity was observed for primary and secondary alkyl bromides, while *tert*-butyl bromide yielded only traces of product. Negligible productivity was observed for alkyl chlorides. We also employed (bromomethyl)­cyclopropane as a radical-clock substrate. Hereby, we exclusively observed the formation of the ring-opened alkene product in low yield consistent with the involvement of radicals throughout catalysis. High reactivity could be obtained with *p*-substituted aryl nucleophiles. Only a trace of product could be detected when employing mesityl magnesium bromide as a sterically demanding coupling partner. Similarly, no reactivity was found in attempted sp^2^–sp^2^ cross-coupling reactions.

**Table 3:**
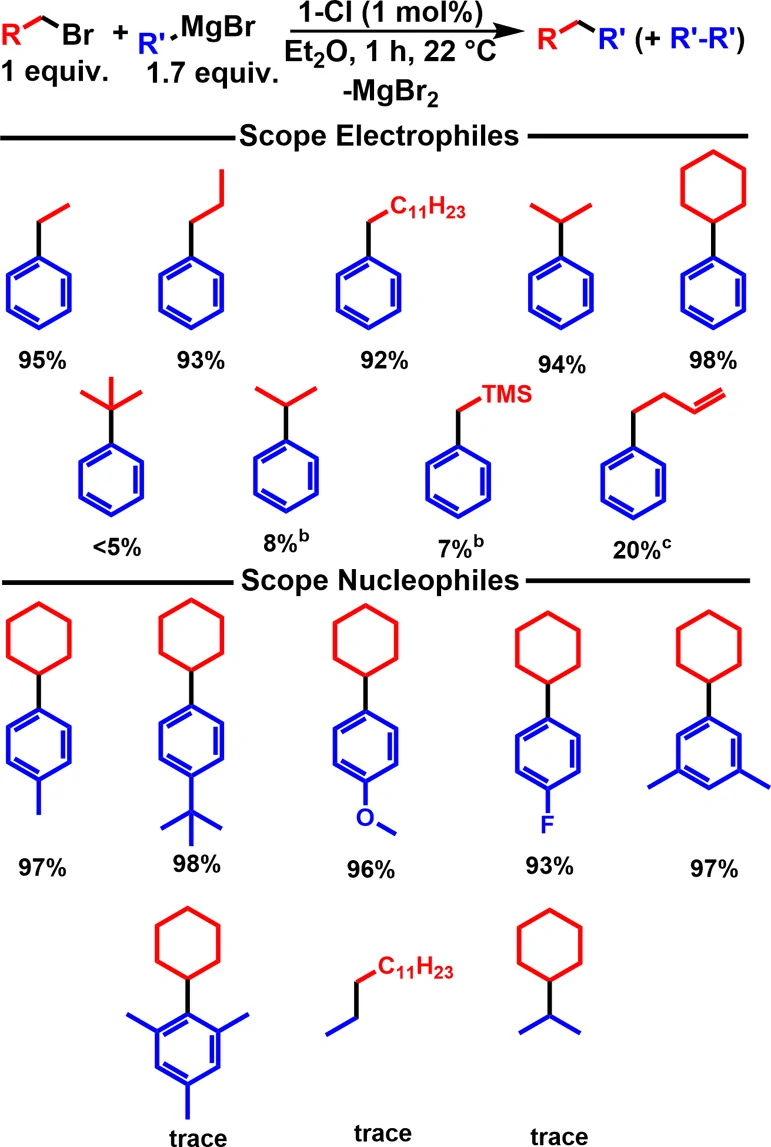
Scope and Limitation of sp^2^–sp^3^ Kumada Cross-Coupling Reaction Catalyzed by **1-Cl** ^a^

a Alkyl chloride instead of alkyl bromide.

b (Bromomethyl)­cyclopropane as an electrophile.

c Reaction conditions: R-Br (0.1 mmol, 1 equiv), R′-MgBr (0.17 mmol, 1.7 equiv), **1-Cl** (0.001 mmol, 1 mol %), Et_2_O (0.1 M), 1 h, 22 °C; yield determined by GC/MS employing *n*-butylbenzene as internal standard.

In sum, we have shown that Mirica’s rigid PNPN seesaw ligand forms well-defined octahedral iron­(II) complexes but also supports 5-fold coordination. Notably, dihalide complexes **1** are low spin and adopt the anticipated *C*_2*v*_-symmetry in the solid-state and solution. Halide abstraction from **1** yielded cationic complexes **2**. Coordinatively unsaturated compounds **2** serve as valuable precursors to give cationic complexes **3**, supported by π-acidic coligands. This leads to a loss of *C*_2*v*_-symmetry as elucidated by structural analysis in the solid-state and in solution. We studied the impact of the ^15^N NMR shifts of ligand substitution on the pyridine donors of the PNPN ligand. Furthermore, the here employed PNPN ligand supports the formation of dicationic complex **4**, when utilizing Fe­(OTf)_2_ as precursor. Finally, the reactivity of all complexes in sp^2^–sp^3^ Kumada cross-coupling was studied. Good productivity was observed for an array of alkyl bromides and aryl bromides, showing similar reactivity compared to typical procedures as introduced by Nakamura^18b^ or Fürstner.^18c^ The potential of the here presented seesaw iron complexes for other cross-coupling reactions is currently under investigation in our laboratories.

## Supplementary Material





## Notes

Optimized geometries (XYZ)
Synthetic procedures, ^1^H, ^13^C­{^1^H}, ^15^N, and ^31^P­{^1^H} NMR spectra, and computational methods (PDF)

## Notes

This article is dedicated to Prof. Karl Kirchner, a pioneer in organometallic chemistry in Austria, on the occasion of his 65^th^ birthday and his approaching retirement.

## References

[1] Jasat A., Dolphin D. (1997). Expanded Porphyrins and Their Heterologs. Chem. Rev..

[2] Mahammed A., Gross Z. (2023). Milestones and Most
Recent Advances in Corrole’s Science and
Technology. J. Am. Chem. Soc..

[3] Che C.-M., Li Z.-Y., Wong K.-Y., Poon C.-K., Mak T. C. W., Peng S.-M. (1994). A simple synthetic
route to N,N′-dialkyl-2,11-diaza[3.3]­(2,6)-pyridinophanes.
Crystal structures of N,N′-di-tert-butyl-2,11-diaza[3.3]­(2,6)­pyridinophane
and its copper­(II) complex. Polyhedron.

[4] Shin S., Choe J., Park Y., Jeong D., Song H., You Y., Seo D., Cho J. (2019). Artificial
Control of Cell Signaling Using a Photocleavable Cobalt­(III)–Nitrosyl
Complex. Angew. Chem., Int. Ed..

[5] Noh H., Jeong D., Ohta T., Ogura T., Valentine J. S., Cho J. (2017). Distinct Reactivity
of a Mononuclear
Peroxocobalt­(III) Species toward Activation of Nitriles. J. Am. Chem. Soc..

[6] Khusnutdinova J. R., Rath N. P., Mirica L. M. (2010). Stable
Mononuclear Organometallic Pd­(III) Complexes and Their C–C
Bond Formation Reactivity. J. Am. Chem. Soc..

[7] Fryzuk M. D., Love J. B., Rettig S. J., Young V. G. (1997). Transformation of
Coordinated Dinitrogen by Reaction with Dihydrogen and Primary Silanes. Science.

[8] Fryzuk M. D., Johnson S. A., Rettig S. J. (1999). Synthesis and Structure
of the Tantalum
Trimethyl Complex [P_2_N_2_]­TaMe_3_ and
Its Conversion to the Tantalum Methylidene Species [P_2_N_2_]­Ta=CH_2_(Me) ([P_2_N_2_] = PhP­(CH_2_SiMe_2_NSiMe_2_CH_2_)_2_PPh). Organometallics.

[9] Ekici S., Nieger M., Glaum R., Niecke E. (2003). A Strategy for the
Synthesis of Phosphorus-Containing Macrocycles - Ligands for Exceptional
Coordination Geometries. Angew. Chem., Int.
Ed..

[10] Fuchigami K., Watson M. B., Tran G. N., Rath N. P., Mirica L. M. (2021). Synthesis
and Reactivity of (N_2_P_2_)Ni Complexes Stabilized
by a Diphosphonite Pyridinophane Ligand. Organometallics.

[11] Bigler R., Otth E., Mezzetti A. (2014). Chiral Macrocyclic
N_2_P_2_ Ligands and Iron­(II): A Marriage of Interest. Organometallics.

[12] Weber S., Pavelic A. M., Peters J. C. (2026). Iron Complexes
Supported by a Dual
Dearomatized PNPN Ligand. Inorg. Chem..

[13] Staroverov V. N., Scuseria G. E., Tao J., Perdew J. P. (2003). Comparative assessment
of a new nonempirical density functional: Molecules and hydrogen-bonded
complexes. J. Chem. Phys..

[14] Tao J., Perdew J. P., Staroverov V. N., Scuseria G. E. (2003). Climbing the Density Functional Ladder: Nonempirical
Meta-Generalized Gradient Approximation Designed for Molecules and
Solids. Phys. Rev. Lett..

[15] Caldeweyher E., Ehlert S., Hansen A., Neugebauer H., Spicher S., Bannwarth C., Grimme S. (2019). A generally applicable
atomic-charge dependent London dispersion correction. J. Chem. Phys..

[16] Hirshfeld F. L. (1977). Bonded-atom
fragments for describing molecular charge densities. Theor. Chim. Acta.

[17] Mathew J., Suresh C. H. (2010). Use of Molecular Electrostatic Potential at the Carbene
Carbon as a Simple and Efficient Electronic Parameter of N-heterocyclic
Carbenes. Inorg. Chem..

[18] Kharasch M. S., Fields E. K. (1941). Factors Determining the Course and
Mechanisms of Grignard Reactions. IV. The Effect of Metallic Halides
on the Reaction of Aryl Grignard Reagents and Organic Halides. J. Am. Chem. Soc..

[19] Weber S., Stöger B., Podewitz M. (2026). Iron Complexes Supported
by a Rigid
Macrocyclic PNPN Ligand. ChemRxiv.

