# Supplementary material for: Iron Complexes Supported by a Rigid Macrocyclic PNPN Ligand
Source: Inorg Chem. 2026 Aug 26;65(35):20171–5. doi: 10.1021/acs.inorgchem.6c02531 (PMC13556124; doi:10.1021/acs.inorgchem.6c02531)
Supplement: Supplementary file 1 [file ic6c02531_si_002.pdf]

Supporting Information for

Iron Complexes Supported by a Rigid Macrocyclic PNPN Ligand

Stefan Weber,[a,*] Berthold Stöger,[b] and Maren Podewitz[c]

Corresponding Author Email: stefan.e163.weber@tuwien.ac.at

[a]Institute of Applied Synthetic Chemistry, TU Wien, Getreidemarkt 9, A-1060 Vienna, AUSTRIA

[b]X-Ray Center, TU Wien, Getreidemarkt 9, A-1060 Vienna, AUSTRIA

[c]Institute of Materials Chemistry, TU Wien, Getreidemarkt 9, A-1060 Vienna, AUSTRIA

Contents:

1 General Information 2
2 Synthetic Procedures 3
3 Catalytic Experiments 8
3.1 General Procedure for Kumada Cross Coupling Reactions 8
4 Computational Modeling and UV/Vis Analysis 9
4.1 Modeling of Complexes 9
4.2 UV/Vis Analysis of Complexes **1** and **2** 9
4.3 Correlations of ^{15}N-shifts with Structural Parameters 12
5 NMR Spectra 17
6 References 46

1 General Information

General Information

All reactions were performed under an inert atmosphere of argon by using Schlenk techniques or in a MBraun inert-gas glovebox. The solvents were purified according to standard procedures. The deuterated solvents were purchased from Euroisotope and dried over 3 Å molecular sieves. PNPN ligand was synthesized according to literature.[1]

NMR spectroscopy

NMR measurements were obtained on 600 MHz Bruker Avance III HD with Prodigy cyroprobe. ^{1}H-NMR and ^{13}C-NMR chemical shifts are reported in ppm relative to tetramethylsilane, using residual protio solvent as an internal standard. ^{15}N-NMR chemical shifts were externally referenced to NH_3 in H_2O. ^{31}P-NMR chemical shifts were externally referenced to H_3PO_4 in H_2O. Solution phase magnetic measurements were performed by the method of Evans utilizing the shift of solvent signal in the solution containing the analyte vs. pure solvent.[2]

Optical and Infrared Spectroscopy

UV/Vis measurements were conducted on a Jasco V-670 spectrophotometer. The light covered the range of 200–800 nm employing tetrahydrofuran or dichloromethane as solvent.
IR measurements were conducted on solid samples via ATR technique on a Perkin Elmer Spectrum 400.

Computational Methods

As initial starting structures x-ray crystal structures were taken whenever possible. In case where no crystal structure was available, respective structures were obtained by modification of available experimental ones. All structures were fully optimized using the hybrid density functional TPSSh[3] which has 10 % of Hartree-Fock exchange and gives good results for relative spin state splittings of Fe-complexes.[4] The triple-zeta basis set with extra polarization functions, def2-TZVPP,[5] was used in conjunction with dispersion corrections of the D4 type to ensure accurate results.[6]
By analysis of the total spin expectation value <S*S> it was ensured that spin contamination was small (below 2.1% deviation from ideal behavior for all complexes **2**). To assess the electronic structure, Hirshfeld partial charges were calculated, which is obtained by partitioning the total electron density based on atomic weights, that is, how much each atomic density contributes in real space here.[7]
All calculations were carried out using version 6.0 of the ORCA package structures were visualized with ChemCraft.[8]
To calculate the Tolman Electronic Parameters (TEP) the corresponding $Ni(CO)_3L$ complexes were modeled employing the level of theory described above whereas the highest CO stretch band represents the TEP.
For the calculation of UV/Vis spectra time-dependent Density Functional Theory was used, employing the CAM-B3YLP[9] density functional in conjunction with the def2-TZVPP basis set and D4 corrections. CAM-B3LYP is a range-separated hybrid functional widely employed to yield accurate excitation energies.[10]

Crystallographic Details

X-ray diffraction data of **1-Br** (CSD 2552058) and **3-tBuNC** (CSD 2552059) were collected at $T = 100$ K in a dry stream of nitrogen on a STOE STADIVARI diffractometer system equipped with a Dectris Eiger CdTe hybrid photon counting detector using Mo-*K*α radiation (λ = 0.71073 Å, **1-Br**) or Cu-*K*α (λ = 1.54186 Å, **3-tBuNC**) radiation. Data were reduced with X-Area and an absorption correction was applied with the multi-scan approach implemented in LANA.[11] The crystal structures were solved by the dual-space approach implemented in SHELXT[12] and refined against F^2 with SHELXL.[13] Non-H atoms were refined with anisotropic atomic displacement parameters. H atoms connected to C were placed in calculated positions and refined as riding on the parent atom. Molecular graphics were generated with the program MERCURY.[14]

2 Synthetic Procedures

(PNPN)$FeCl_2$ (1-Cl). PNPN ligand (197 mg, 0.5 mmol, 1.05 equiv.) and $FeCl_2$ (60 mg, 0.48mmol, 1 equiv.) were combined as solids. THF (20 mL) was added and the obtained red suspension was stirred for 16 hours. The suspension was concentrated to approx. 3 mL, Et_2O (20 mL) was added. The supernatant was decanted and the red solid washed with Et_2O (3 x 15 mL). The residue was extracted with DCM (5 x 20 mL), filtered and the solvent was removed, yielding **1-Cl** as red solid (234 mg, 93%).

*Note: Despite rigorous drying, approx. one equivalent of DCM remained in the final product presumably as stable solvate. We could observe that complexes **1** are "bench stable" as solids over weeks.*

^{1}H NMR (600 MHz, *MeOD-d$_4$*, 25 °C): δ = 7.38 (t, *J* = 8.2 Hz, 2H, C3 pyr), 6.64 (d, *J* = 7.0 Hz, 4H, C2/C5 pyr), 1.84 (s, 18H, ^{t}Bu) ppm.

^{13}C{^{1}H} NMR (150 MHz, *MeOD-d$_4$*, 25 °C): δ = 165.5 (C3 pyr), 144.2 (C1/C5 pyr), 105.1 (C2/C5 pyr), 41.1 (broad, ^{t}Bu (C_q)), 24.6 (^{t}Bu (CH_3)) ppm.

^{31}P{^{1}H} NMR (241 MHz, *MeOD-d$_4$*, 25 °C): δ = 243.5 ppm.

^{15}N NMR (61 MHz, *MeOD-d$_4$*, 25 °C): δ = 205.4 ppm.

Elemental analysis: Anal. Calcd. for $C_{18}H_{24}Cl_2FeN_2O_4P_2 \cdot CH_2Cl_2$: C, 38.87; H, 4.24; N, 4.53. Found: C, 38.98; H, 4.42; N, 4.39.

(PNPN)$FeBr_2$ (1-Br). PNPN ligand (197 mg, 0.5 mmol, 1.05 equiv.) and $FeBr_2$ (104 mg, 0.48mmol, 1 equiv.) were combined as solids. THF (20 mL) was added and the obtained red suspension was stirred for 16 hours. The suspension was concentrated to approx. 2 mL, Et_2O (20 mL) was added. The supernatant was decanted and the red solid washed with Et_2O (3 x 5 mL). The residue was extracted with DCM (2 x 10 mL), filtered and the solvent was removed, yielding **1-Br** as red solid (287 mg, 98%). Single crystals were grown by layering a concentrated DCM solution of **1-Br** with *n*-heptane.

^{1}H NMR (600 MHz, *DCM-d$_2$*, 25 °C): δ = 7.45 (t, *J* = 8.1 Hz, 2H, C3 pyr), 6.71 (d, *J* = 8.1 Hz, 4H, C2/C5 pyr), 2.14 (s, 18H, ^{t}Bu) ppm.

^{13}C{^{1}H} NMR (150 MHz, *DCM-d$_2$*, 25 °C): δ = 165.5 (C3 pyr), 142.2 (C1/C5 pyr), 104.4 (C2/C5 pyr), 41.8 (t, J = 8.5 Hz, ^{t}Bu (C_q)), 26.0 (^{t}Bu (CH_3)) ppm.

^{31}P{^{1}H} NMR (241 MHz, *DCM-d$_2$*, 25 °C): δ = 249.0 ppm.

^{15}N NMR (61 MHz, *DCM-d$_2$*, 25 °C): δ = 215.2 ppm.

Elemental analysis: Anal. Calcd. for $C_{18}H_{24}Br_2FeN_2O_4P_2$: C, 35.44; H, 3.97; N, 4.59. Found: C, 35.61; H, 4.14; N, 4.72.

(PNPN)FeI_2 (1-I). PNPN ligand (197 mg, 0.5 mmol, 1.05 equiv.) and FeI_2 (148 mg, 0.48mmol, 1 equiv.) were combined as solids. THF (20 mL) was added and the obtained red suspension was stirred for 16 hours. The solvent was removed and the residue washed with *n*-pentane/Et_2O (1:1 3 x 10 mL) The residue was extracted with DCM (2 x 5 mL), filtered and the solvent was removed, yielding **1-I** as red solid (297 mg, 88%).

^{1}H NMR (600 MHz, *DCM-d$_2$*, 25 °C): δ = 7.45 (t, *J* = 8.1 Hz, 2H, C3 pyr), 6.72 (d, *J* = 8.1 Hz, 4H, C2/C4 pyr), 2.22 (t, *J* = 8.4 Hz, 18H, ^{t}Bu) ppm.

^{13}C{^{1}H} NMR (150 MHz, *DCM-d$_2$*, 25 °C): δ = 165.0 (t = 4.0 Hz, C3 pyr), 141.9 (C1/C5 pyr), 104.3 (C2/C4 pyr), 43.8 (t, J = 13.8 Hz, ^{t}Bu (C_q)), 27.6 (^{t}Bu (CH_3)) ppm.

^{31}P{^{1}H} NMR (241 MHz, *DCM-d$_2$*, 25 °C): δ = 258.1 ppm.

^{15}N NMR (61 MHz, *DCM-d$_2$*, 25 °C): δ = 221.7 ppm.

Elemental analysis: Anal. Calcd. for $C_{18}H_{24}I_2FeN_2O_4P_2$: C, 30.71; H, 3.44; N, 3.98. Found: C, 31.01; H, 3.57; N, 3.82.

[(PNPN) FeCl] BArF$_{24}$ (2-Cl). 1-Cl (100 mg, 0.19 mmol, 1 equiv.) and NaBArF$_{24}$ (170 mg, 0.19 mmol, 1equiv.) were suspended in DCM (5 mL) and stirred for 2 hours, yielding a red solution and formation of a white solid. The suspension was filtered and the solvent was removed. The red residue was taken up in DCM (2 mL), filtered and the solvent was removed, yielding **2-Cl** as red solid (234 mg, 91%).

*Note: **2-Cl** is paramagnetic, but we could still detect and assign the resonances in ^{1}H- and ^{31}P-NMR.*

^{1}H NMR (600 MHz, *DCM-d$_2$*, 25 °C): δ = 13.63 (t, *J* = 9.0 Hz, 2H, C3 pyr), 13.39 (d, *J* = 7.9 Hz, 4H, C2/C4 pyr), 7.66 (s, 8H, BArF$_{24}$), 7.46 (s, 4H, BArF$_{24}$), -13.22 (broad, 18H, ^{t}Bu) ppm.

^{31}P{^{1}H} NMR (241 MHz, *DCM-d$_2$*, 25 °C): δ = 160.3 ppm.

μ_{eff}(Evans' Method, CD_2Cl_2, 25°C) = 2.9 μ_B

μ_{eff}(Evans' Method, CD_2Cl_2, 37°C) = 2.8 μ_B

Elemental analysis: Anal. Calcd. for $C_{50}H_{36}BClF_{24}FeN_2O_4P_2$: C, 44.52; H, 2.69; N, 2.08. Found: C, 44.83; H, 2.62; N, 1.80.

[(PNPN) FeBr] BArF$_{24}$ (2-Br). 1-Br (100 mg, 0.16 mmol, 1 equiv.) and NaBArF$_{24}$ (145 mg, 0.16 mmol, 1equiv.) were dissolved in DCM (5 mL) and stirred for 2 hours, yielding a red solution and formation of a white solid. The suspension was filtered and the solvent was removed. The red residue was taken up in DCM (2 mL), filtered and the solvent was removed, yielding **2-Br** as red solid (219 mg, 96%).

μ_{eff}(Evans' Method, CD_2Cl_2, 25°C) = 1.6 μ_B

μ_{eff}(Evans' Method, CD_2Cl_2, 37°C) = 1.5 μ_B

μ_{eff}(Evans' Method, $CDCl_3$, 23°C) = 2.0 μ_B

μ_{eff}(Evans' Method, $CDCl_3$, 40°C) = 1.5 μ_B

μ_{eff}(Evans' Method, $CDCl_3$, 50°C) = 1.4 μ_B

μ_{eff}(Evans' Method, $CDCl_3$, 60°C) = 1.2 μ_B

Elemental analysis: Anal. Calcd. for $C_{50}H_{36}BBrF_{24}FeN_2O_4P_2$: C, 43.10; H, 2.60; N, 2.01. Found: C, 43.32; H, 2.77; N, 1.84.

[(PNPN) FeI] BArF$_{24}$ (2-I). 1-I (100 mg, 0.14 mmol, 1 equiv.) and NaBArF$_{24}$ (126 mg, 0.14 mmol, 1equiv.) were dissolved in DCM (5 mL) and stirred for 2 hours, yielding a red solution and formation of a white solid. The suspension was filtered and the solvent was removed. The red residue was taken up in DCM (2 mL), filtered and the solvent was removed, yielding **2-I** as orange-brown solid (199 mg, 97%).

μ_{eff}(Evans' Method, CD_2Cl_2, 25°C) = 1.3 μ_B

μ_{eff}(Evans' Method, CD_2Cl_2, 37°C) = 1.2 μ_B

μ_{eff}(Evans' Method, $CDCl_3$, 23°C) = 1.0 μ_B

μ_{eff}(Evans' Method, $CDCl_3$, 40°C) = 1.0 μ_B

μ_{eff}(Evans' Method, $CDCl_3$, 50°C) = 1.1 μ_B

μ_{eff}(Evans' Method, $CDCl_3$, 60°C) = 1.2 μ_B

Elemental analysis: Anal. Calcd. for $C_{50}H_{36}BIF_{24}FeN_2O_4P_2$: C, 41.70; H, 2.52; N, 1.94. Found: C, 41.34; H, 2.79; N, 1.71.

[(PNPN)(MeCN) FeBr] $BArF_{24}$ (3-MeCN). A freshly prepared, filtered DCM solution (2 mL) of **2-Br** (from 0.1 mmol **1-Br** and 0.1 mmol Na $BArF_{24}$) was treated with acetonitrile (21 mg, 26 µL, 0.5 mmol, 5 equiv.). An instant color change from red to yellow-orange was observed and the solution was stirred for one hour. Upon removal of the solvent **3-MeCN** was obtained as yellow solid (142 mg, 99%).

*Note: Complexes **3** can also be prepared from isolated **2-Br**.*

NMR Nomenclature for complexes 3

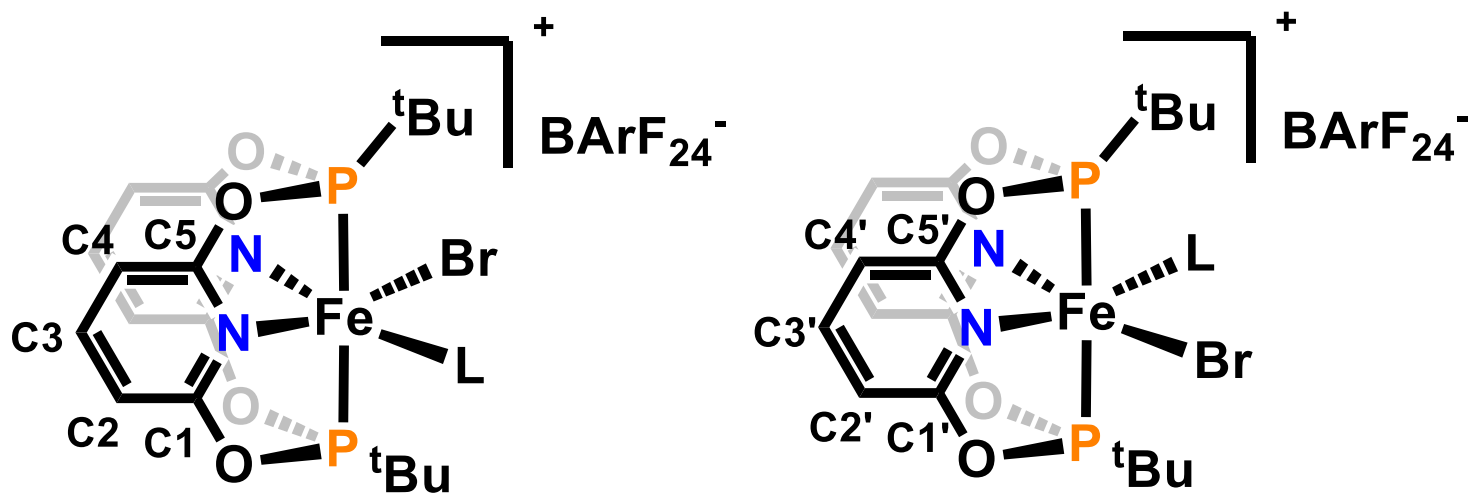

pyridine donor *trans* bromide ligand | **pyridine donor *trans* neutral ligand**

^{1}H NMR (600 MHz, *DCM-d$_2$*, 25 °C): δ = 7.72 (s, 8H, $BArF_{24}$), 7.66 (t, *J* = 8.1 Hz, 1H, C3'), 7.58 (t, *J* = 8.1 Hz, 1H, C3), 7.55 (s, 4H, $BArF_{24}$), 6.87 (d, *J* = 8.1 Hz, 2H, C2/C4), 6.82 (d, *J* = 8.1 Hz, 2H, C2'/C4'), 2.17 (s, 3H, MeCN), 2.02 (t, *J* = 9.1 Hz, 18H) ppm.

^{13}C{^{1}H} NMR (150 MHz, *DCM-d$_2$*, 25 °C): δ = 164.7 (C3'), 164.1 (C3), 162.2 (q, J = 75.5 Hz, $BArF_{24}$) 144.8 (C1/C5+C1'/C5', 136.2 (ACN(q)), 135.2 ($BArF_{24}$), 129.4 (q , J = 43.8 Hz, $BArF_{24}$), 124.3 (q, J = 275.1 Hz, $BArF_{24}$), 117.9 ($BArF_{24}$), 106.3 (C2/C4), 106.2 (C2'/C4'), 40.7 (t, J = 14.1 Hz, ^{t}Bu (C_q)), 25.0(^{t}Bu (CH_3)), 5.19 (MeCN, CH_3) ppm.

^{31}P{^{1}H} NMR (241 MHz, *DCM-d$_2$*, 25 °C): δ = 251.3 ppm.

^{15}N NMR (61 MHz, *DCM-d$_2$*, 25 °C): δ = 208.1 (N *trans* bromide), 195.3 (N *trans* MeCN) ppm.
Note: MeCN not observed.

Elemental analysis: Anal. Calcd. for $C_{52}H_{39}BBrF_{24}FeN_3O_4P_2$: C, 43.54; H, 2.74; N, 2.93. Found: C, 43.66; H, 2.85; N, 2.77.

[(PNPN)(Pyr) FeBr] $BArF_{24}$ (3-Pyr). A freshly prepared, filtered DCM solution (2 mL) of **2-Br** (from 0.1 mmol **1-Br** and 0.1 mmol Na $BArF_{24}$) was treated with pyridine (40 mg, 40 µL, 0.5 mmol, 5 equiv.). An instant color change from red to orange was observed and the solution was stirred for one hour. Upon removal of the solvent **3-Pyr** was obtained as yellow-brown solid (146 mg, 99%).

^{1}H NMR (600 MHz, *DCM-d$_2$*, 25 °C): δ = 7.72 (s, 8H, $BArF_{24}$), 7.69 – 7.61 (m, 5H, C3+C3'+pyr monodentate), 7.55 (s, 4H, $BArF_{24}$), 7.05 (br, 2H pyr monodentate), 6.93 (d, *J* = 8.1 Hz, 2H, C2/C4), 6.82 (d, *J* = 7.3 Hz, 2H, C2'/C4'), 1.78 (t, *J* = 7.9 Hz, 18H, ^{t}Bu) ppm.

^{13}C{^{1}H} NMR (150 MHz, *DCM-d$_2$*, 25 °C): δ = 165.2 (C3'), 164.1 (C3), 162.2 (q, J = 75.5 Hz, $BArF_{24}$) 145.1 (C1'/C5'), 144.7(C1/C5), 138.6 (pyr monodentate), 135.2 ($BArF_{24}$), 127.7(pyr, monodentate) 129.4 (q, J = 43.8 Hz, $BArF_{24}$), 125.7 (pyr, monodentate), 124.3 (q, J = 275.1 Hz, $BArF_{24}$), 117.9 ($BArF_{24}$), 106.6 (C2/C4), 106.1 (C2'/C4'), 40.5 (t, J = 12.6 Hz, ^{t}Bu (C_q)), 24.9(^{t}Bu (CH_3)) ppm.

^{31}P{^{1}H} NMR (241 MHz, *DCM-d$_2$*, 25 °C): δ = 249.0 ppm.

^{15}N NMR (61 MHz, *DCM-d$_2$*, 25 °C): δ = 202.6 (N *trans* bromide), 199.1 (N *trans* pyr) ppm.
Note: pyridine monodentate not observed.

Elemental analysis: Anal. Calcd. for $C_{55}H_{41}BBrF_{24}FeN_3O_4P_2$: C, 44.87; H, 2.81; N, 2.85. Found: C, 44.66; H, 2.98; N, 2.67.

[(PNPN)(PPh$_3$) FeBr] BArF$_{24}$ (3-PPh$_3$). A freshly prepared, filtered DCM solution (2 mL) of **2-Br** (from 0.01 mmol **1-Br** and 0.01 mmol Na BArF$_{24}$) was treated with triphenylphosphine (52 mg, 0.2 mmol, 2 equiv.). An instant color change from red to brown was observed and the solution was stirred for one hour. The solvent was removed and the residue washed with *n*-pentane (7 x 1 mL), yielding **3-PPh$_3$** as brown solid (123 mg, 74%).

^{1}H NMR (600 MHz, *DCM-d$_2$*, 25 °C): δ = 7.82 (t, J = 9.4 Hz, 1H, C3'), 7.72 (s, 8H, BArF$_{24}$), 7.55 (s, 4H, BArF$_{24}$), 7.39 – 7.19 (m, 15H, PPh$_3$), 7.15 (t, J = 8.2 Hz, 1H, C3), 7.00 (d, *J* = 8.5 Hz, 2H, C2/C4), 6.25 (d, *J* = 7.9 Hz, 2H, C2'/C4'), 1.52 (t, *J* = 9.0 Hz, 18H, ^{t}Bu) ppm.

^{13}C{^{1}H} NMR (150 MHz, *DCM-d$_2$*, 25 °C): δ = 163.0 (C3'), 162.3 (C3), 162.2 (q, J = 75.5 Hz, BArF$_{24}$) 145.1 (C1'/C5'), 143.4(C1/C5), 135.2 (BArF$_{24}$), 132.0-131.1 (m, PPh$_3$), 129.8- 129.3 (m, PPh$_3$), 129.4 (q, J = 43.8 Hz, BArF$_{24}$), 124.3 (q, J = 275.1 Hz, BArF$_{24}$), 117.9 (BArF$_{24}$), 106.0 (C2/C4), 105.9 (C2'/C4'), 42.8 (t, J = 13.3 Hz, ^{t}Bu (C$_q$)), 25.1(^{t}Bu (CH$_3$)) ppm.

^{31}P{^{1}H} NMR (241 MHz, *DCM-d$_2$*, 25 °C): δ = 240.9 (d, J = 42.3 Hz, 2P, PNPN), 37.0 (t, J = 42.3 Hz, 1P, PPh$_3$) ppm.

^{15}N NMR (61 MHz, *DCM-d$_2$*, 25 °C): δ = 215.8 (N *trans* bromide), 205.3 (N *trans* PPh$_3$) ppm.

Elemental analysis: Anal. Calcd. for $C_{68}H_{52}BBrF_{24}FeN_2O_4P_3$: C, 49.30; H, 3.16; N, 1.69. Found: C, 49.25; H, 3.32; N, 1.52.

[(PNPN)(PMe$_3$) FeBr] BArF$_{24}$ (3-PMe$_3$). A freshly prepared, filtered DCM solution (2 mL) of **2-Br** (from 0.1 mmol **1-Br** and 0.1 mmol Na BArF$_{24}$) was treated with PMe$_3$ (1M solution in THF, 0.2 mL, 0.2 mmol, 2 equiv.). An instant color change from red to dark red was observed and the solution was stirred for one hour. Upon removal of the solvent **3-PMe$_3$** was obtained as red solid (144 mg, 98%).

^{1}H NMR (600 MHz, *DCM-d$_2$*, 25 °C): δ = 7.85 (t, J = 7.9 Hz, 1H, C3') 7.72 (s, 8H, BArF$_{24}$), 7.56 (t, J = 8.5 Hz, 1H, C3), 7.55 (s, 4H, BArF$_{24}$), 7.06 (d, *J* = 8.5 Hz, 2H, C2/C4), 6.78 (d, *J* = 8.1 Hz, 2H, C2'/C4'), 1.86 (t, *J* = 8.4 Hz, 18H, ^{t}Bu), 1.42 (d, *J* = 9.5 Hz, 9H, PMe$_3$) ppm.

^{13}C{^{1}H} NMR (150 MHz, *DCM-d$_2$*, 25 °C): δ = 163.8 (C3'), 162.2 (q, J = 75.5 Hz, BArF$_{24}$), 162.1 (C3), 144.9 (C1'/C5'), 143.6(C1/C5), 135.2 (BArF$_{24}$), 129.4 (q, J = 43.8 Hz, BArF$_{24}$), 124.3 (q, J = 275.1 Hz, BArF$_{24}$), 117.9 (BArF$_{24}$), 106.0 (C2/C4), 105.9 (C2'/C4'), 42.1 (t, J = 14.0 Hz, ^{t}Bu (C$_q$)), 26.3(^{t}Bu (CH$_3$)), 22.5 (d, J = 29.2 Hz, PMe$_3$) ppm.

^{31}P{^{1}H} NMR (241 MHz, *DCM-d$_2$*, 25 °C): δ = 248.2 (d, J = 48.8 Hz, 2P, PNPN), 14.9 (t, J = 48.8 Hz, 1P, PMe$_3$) ppm.

^{15}N NMR (61 MHz, *DCM-d$_2$*, 25 °C): δ = 218.2 (N *trans* bromide), 200.5 (N *trans* PMe$_3$) ppm.

Elemental analysis: Anal. Calcd. for $C_{53}H_{45}BBrF_{24}FeN_2O_4P_3$: C, 43.32; H, 3.09; N, 1.91. Found: C, 43.43; H, 3.17; N, 1.82.

[(PNPN)(^{t}BuNC) FeBr] BArF$_{24}$ (3-^{t}BuNC). A freshly prepared, filtered DCM solution (2 mL) of **2-Br** (from 0.1 mmol **1-Br** and 0.1 mmol Na BArF$_{24}$) was treated with ^{t}BuNC (1M solution in THF, 0.2 mL, 0.2 mmol, 2 equiv.). An instant color change from red to pink was observed and the solution was stirred for one hour. Upon removal of the solvent **3-^{t}BuNC** was obtained as pink solid (147 mg, 99%). Single crystals were grown by layering a concentrated DCM solution of **1-Br** with *n*-heptane.

^{1}H NMR (600 MHz, *DCM-d$_2$*, 25 °C): δ = 7.84 (t, J = 8.1 Hz, 1H, C3') 7.72 (s, 8H, BArF$_{24}$), 7.63 (t, J = 7.8 Hz, 1H, C3), 7.55 (s, 4H, BArF$_{24}$), 7.07 (d, *J* = 7.8 Hz, 2H, C2/C4), 6.78 (d, *J* = 7.8 Hz, 2H, C2'/C4'), 1.91 (t, *J* = 9.2 Hz, 18H, ^{t}Bu), 1.37 (s, 9H, ^{t}BuNC(^{t}Bu)) ppm.

^{13}C{^{1}H} NMR (150 MHz, *DCM-d$_2$*, 25 °C): δ = 163.6 (C3'), 162.7 (C3), 162.2 (q, J = 75.5 Hz, BArF$_{24}$), 151.9 (^{t}BuNC (C$_q$ coordinated to Fe)), 145.4 (C1'/C5'), 144.4(C1/C5), 135.2 (BArF$_{24}$), 129.4 (q, J = 43.8 Hz, BArF$_{24}$), 124.3 (q, J = 275.1 Hz, BArF$_{24}$), 117.9 (BArF$_{24}$), 106.6 (C2/C4), 106.2 (C2'/C4'), 40.7 (t, J = 13.1 Hz, ^{t}Bu (C$_q$)), 30.6 (^{t}BuNC (^{t}Bu (C$_q$)), 30.5 (^{t}BuNC (^{t}Bu (CH$_3$)), 25.0(^{t}Bu (CH$_3$)) ppm.

^{31}P{^{1}H} NMR (241 MHz, *DCM-d$_2$*, 25 °C): δ = 247.9 ppm.

^{15}N NMR (61 MHz, *DCM-d_2*, 25 °C): δ = 215.4 (N *trans* bromide), 204.1 (N *trans* ^{t}BuNC) ppm.
Note: ^{t}BuNC not observed.

ATR-IR (solid): 2143 cm^{-1}.

Elemental analysis: Anal. Calcd. for $C_{55}H_{45}BBrF_{24}FeN_3O_4P_2$: C, 44.74; H, 3.07; N, 2.85. Found: C, 44.99; H, 3.01; N, 2.72.

[(PNPN)(CO) FeBr] $BArF_{24}$ (3-CO). A freshly prepared, filtered DCM solution (2 mL) of **2-Br** (from 0.1 mmol **1-Br** and 0.1 mmol Na $BArF_{24}$) was subjected to three pump-freeze-thaw cycles and then backfilled with CO gas. An instant color change from red to dark red was observed and the solution was stirred for one hour. Upon removal of the solvent **3-CO** was obtained as dark red solid (142 mg, 99%).

^{1}H NMR (600 MHz, *DCM-d_2*, 25 °C): δ = 7.96 (t, J = 8.6 Hz, 1H, C3') 7.72 (s, 8H, $BArF_{24}$), 7.70 (t, J = 9.2 Hz, 1H, C3), 7.55 (s, 4H, $BArF_{24}$), 7.18 (d, *J* = 8.4 Hz, 2H, C2/C4), 6.90 (d, *J* = 8.6 Hz, 2H, C2'/C4'), 1.88 (t, *J* = 9.8 Hz, 18H, ^{t}Bu) ppm.

$^{13}C\{^{1}H\}$ NMR (150 MHz, *DCM-d_2*, 25 °C): δ = 210 (t, J = 21.8 Hz, CO), 162.3 (C3'), 162.2 (q, J = 75.5 Hz, $BArF_{24}$), 161.8 (C3), 147.2 (C1'/C5'), 146.3(C1/C5), 135.2 ($BArF_{24}$), 129.4 (q, J = 43.8 Hz, $BArF_{24}$), 124.3 (q, J = 275.1 Hz, $BArF_{24}$), 118.0 ($BArF_{24}$), 107.7 (C2/C4), 107.4 (C2'/C4'), 40.9 (t, J = 16.6 Hz, ^{t}Bu (C_q)), 24.7(^{t}Bu (CH_3)) ppm.

$^{31}P\{^{1}H\}$ NMR (241 MHz, *DCM-d_2*, 25 °C): δ = 254.9 ppm.

^{15}N NMR (61 MHz, *DCM-d_2*, 25 °C): δ = 215.6 (N *trans* bromide), 188.6 (N *trans* CO) ppm.

ATR-IR (solid): 2046 cm^{-1}.

Elemental analysis: Anal. Calcd. for $C_{51}H_{36}BBrF_{24}FeN_2O_5P_2$: C, 43.10; H, 2.55; N, 1.97. Found: C, 43.32; H, 2.69; N, 1.78.

[(PNPN)$(MeCN)_2$ Fe] $(OTf)_2$ (4). PNPN ligand (150 mg, 0.38 mmol, 1.1 equiv.) and $Fe(OTf)_2$ (122 mg, 0.35 mmol, 1 equiv.) were combined as solids. MeCN (7 mL) were added and stirred for 3 hours. The solution was concentrated to approx. 0.5 mL and Et_2O (10 mL) leading to the formation of a yellow-orange solid. The solution was decanted, the solid washed with Et_2O (2x 10 mL) and, the solid was dried yielding **4** as yellow solid (240 mg, 84%).

^{1}H NMR (600 MHz, *DCM-d_2*, 25 °C): δ = 7.94 (t, *J* = 8.1 Hz, 2H, C3 pyr), 7.06 (d, *J* = 7.3 Hz, 4H, C2/C4 pyr), 2.34 (s, 6H, ACN), 1.99 (t, *J* = 9.9 Hz, 18H, ^{t}Bu) ppm.

$^{13}C\{^{1}H\}$ NMR (150 MHz, *DCM-d_2*, 25 °C): δ = 163.9 (t = 3.9 Hz, C3 pyr), 147.3 (C1/C5 pyr), 139.4 (MeCN (C_q)), 107.8 (C2/C4 pyr), 39.7 (t, J = 14.0 Hz, ^{t}Bu (C_q)), 24.1 (^{t}Bu (CH_3)), 5.4 (MeCN (CH_3)) ppm. *Note: CF_3 not observed.*

$^{31}P\{^{1}H\}$ NMR (241 MHz, *DCM-d_2*, 25 °C): δ = 251.3 ppm.

^{15}N NMR (61 MHz, *DCM-d_2*, 25 °C): δ = 189.5 (PNPN), 155.5 (MeCN) ppm.

Elemental analysis: Anal. Calcd. for $C_{24}H_{30}F_6FeN_4O_{10}P_2S_2$: C, 34.71; H, 3.64; N, 6.75. Found: C, 34.61; H, 3.32; N, 6.60.

3 Catalytic Experiments

3.1 General Procedure for Kumada Cross Coupling Reactions

An 8 mL screw cap vial was charged with iron complex (0.0005-0.003 mmol; 0.5-3 mol%), a stirring bar and solvent (0.5 mL) was added. For catalyst loading of 1 mol% and below, a THF stock solution was employed. Alkyl halide substrate (0.1 mmol, 1 equiv.) was added and the reaction was stirred for approx. 2 minutes. Grignard reagent was diluted with the employed solvent to give a 0.35M solution and 0.5 mL (0.17 mmol, 1.7 equiv.) of this solution was added dropwise to the reaction mixture over ~1 minute. The reaction mixture was stirred for one hour at 22 °C (room temperature). Butylbenzene (15.6 µL, 0.1 mmol, 1 equiv.) as internal standard was added, the reaction mixture was quenched with a saturated NH_4Cl solution (0.5 mL), and diluted with *n*-pentane (5 mL). A sample of the organic phase was taken, dried over Na_2SO_4 and analyzed by GC/MS. If found necessary, the products from catalytic experiments were compared with GC/MS traces of authentic samples (in order to distinguish between e.g., *n*-propyl vs. *iso*-propyl benzene).

Table S 1. Optimization reactions for the cross coupling of cyclohexyl bromide and phenyl magnesium bromide.

Complex (mol%)	Solvent	Conversion of Electrophile [%]	Yield [%]
**1-Cl** (3)	THF	>99	70
**1-Br** (3)	THF	>99	58
**1-I** (3)	THF	>99	59
**2-Cl** (3)	THF	>99	67
**2-Br** (3)	THF	>99	63
**2-I** (3)	THF	>99	64
**3-MeCN** (3)	THF	>99	59
**3-Pyr** (3)	THF	>99	56
**3-PPh_3** (3)	THF	>99	62
**3-PMe_3** (3)	THF	>99	58
**3-^{t}BuNC** (3)	THF	67	43
**3-CO** (3)	THF	49	27
**4** (3)	THF	>99	61
**1-Cl** (3)	Et_2O	>99	99
**1-Cl** (2)	Et_2O	>99	98
**1-Cl (1)**	**Et_2O**	**>99**	**98**
**1-Cl** (0.5)	Et_2O	59	48
$FeCl_2$ (salt) (3)	Et_2O	>99	36
$FeBr_2$ (salt) (3)	Et_2O	>99	32
FeI_2 (salt) (3)	Et_2O	>99	35
No Fe	Et_2O	<5	trace

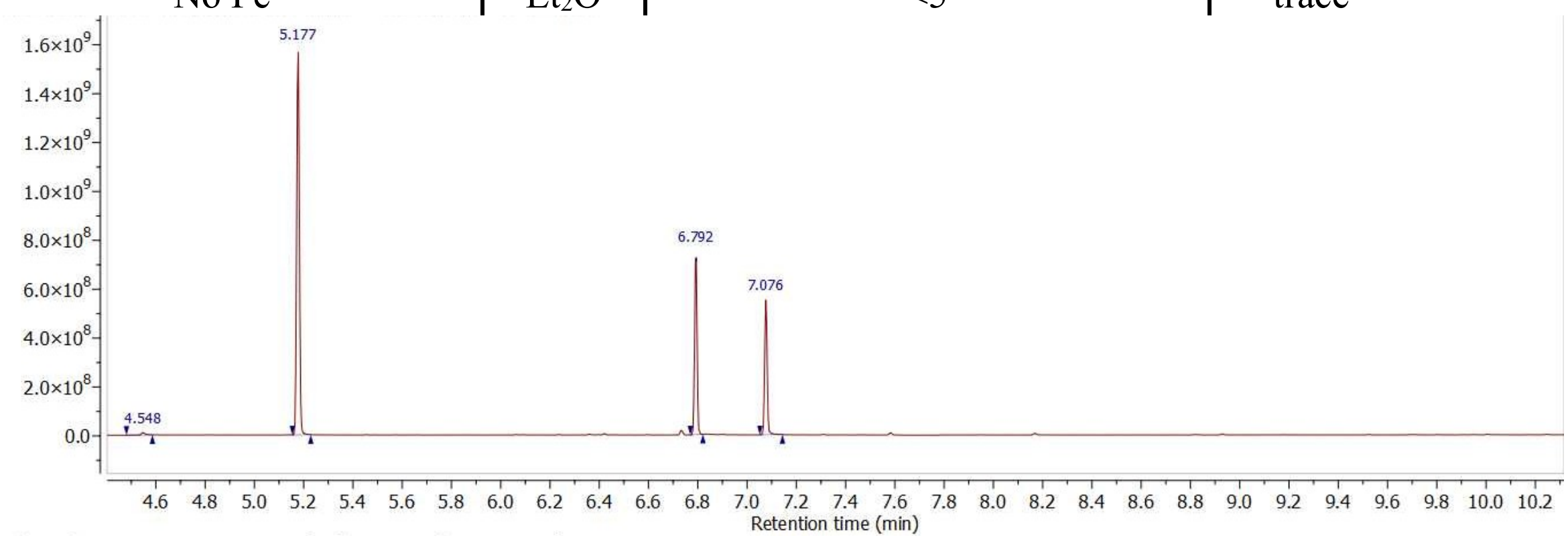

Figure S 1. Representative GC spectrum of Kumada sp^2-sp^3 cross coupling of cyclohexyl bromide (RT = 4.55 min) with PhMgBr, yielding phenyl cyclohexane (RT = 6.79 min) as product and biphenyl (RT = 7.07 min) as side product. Yield determined against n-butyl benzene (RT = 5.18 min).

4 Computational Modeling and UV/Vis Analysis

4.1 Modeling of Complexes

*Table S 2. Calculated Single Point Energies for Complexes **1, 3,** and **4**.*

Complex	Single Point Energy [a.u.]
**1-Cl**	-3978.532941422170
**1-Br**	-8206.332839004404
**1-I**	-3653.412772190142
**3-MeCN**	-5764.872960371172
**3-Pyr**	-5880.471684288646
**3-PPh$_3$**	-6668.775783955152
**3-PMe$_3$**	-6093.269253584988
**3-tBuNC**	-5882.870471689169
**3-CO**	-5745.428305179773
**4**	-3322.550982686781

*Table S 3. Calculated Energies for **2-Cl** and experimentally determined μ_{eff} at room temperature.*

Spin State	Relative Energy [kcal/mol]	Experimental μ_{eff}
Singlet	1.8	2.9
**Triplet**	**0**	
Quintet	4.9	

*Table S 4. Calculated Energies for **2-Br** and experimentally determined μ_{eff} at room temperature.*

Spin State	Relative Energy [kcal/mol]	Experimental μ_{eff}
Singlet	2.5	1.6
**Triplet**	**0**	
Quintet	6.4	

*Table S 5. Calculated Energies for **2-I** and experimentally determined μ_{eff} at room temperature.*

Spin State	Relative Energy [kcal/mol]	Experimental μ_{eff}
Singlet	5.4	1.3
**Triplet**	**0**	
Quintet	8.4	

4.2 UV/Vis Analysis of Complexes 1 and 2

Time-Dependent DFT (TD-DFT) was employed to model the UV/Vis spectra of complexes **1** and **2** on a CAM-B3LYP/def2-TZVPP/D4 level using 100 roots without modeling the $BArF_{24}$ counterion or solvent interactions. The calculated spectra were broadened by employing Lorentzian line broadening (factor 20 at half height). The experimental data for all complexes are in good agreement with the calculated spectra and are consistent with the assumption of the population of multiple spin states within the series of complexes **2**.

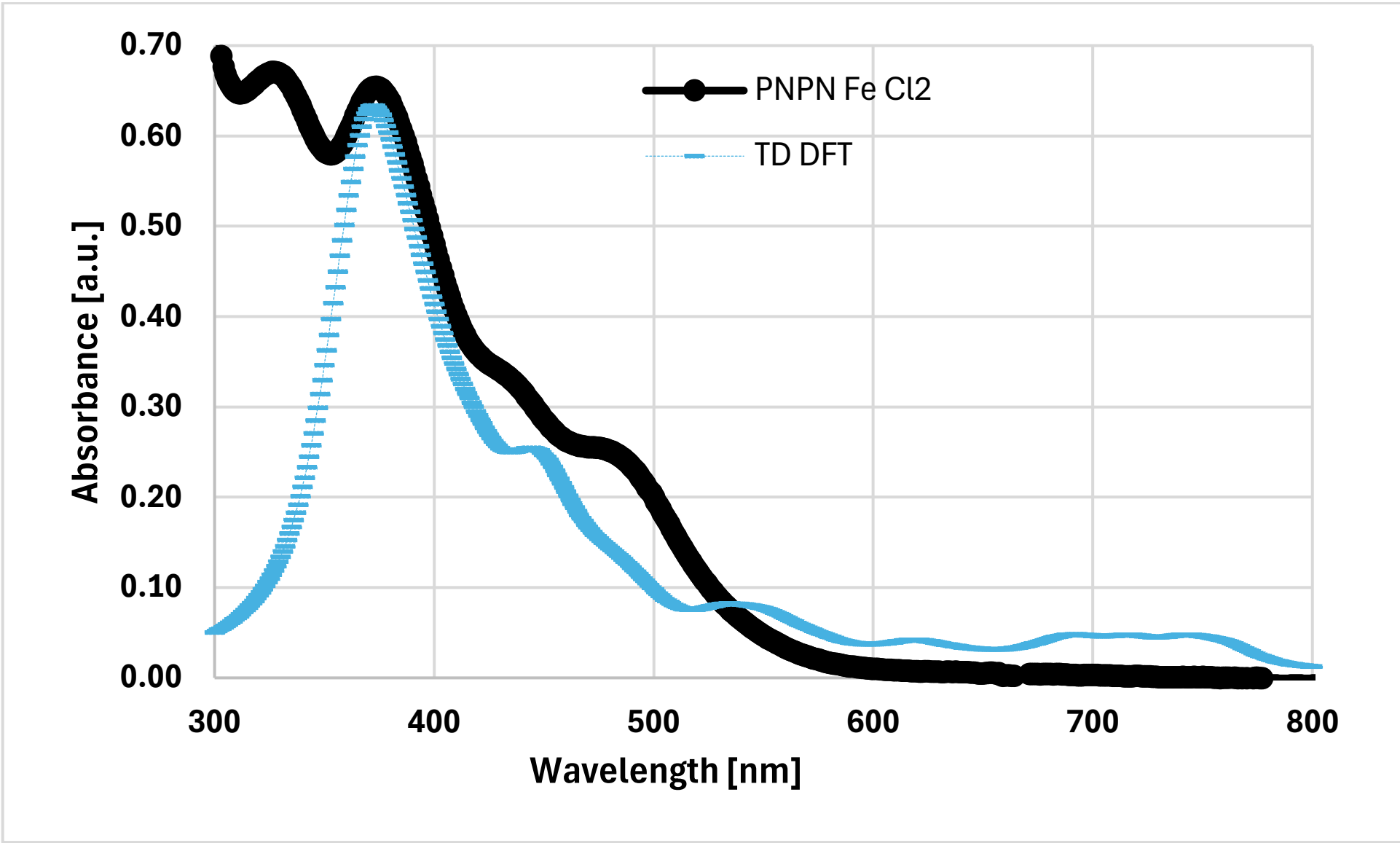

Figure S 2. UV/Vis spectrum of 1-Cl in THF (ca. 0.1 mM) at 23°C (black) and TD-DFT spectrum (blue).

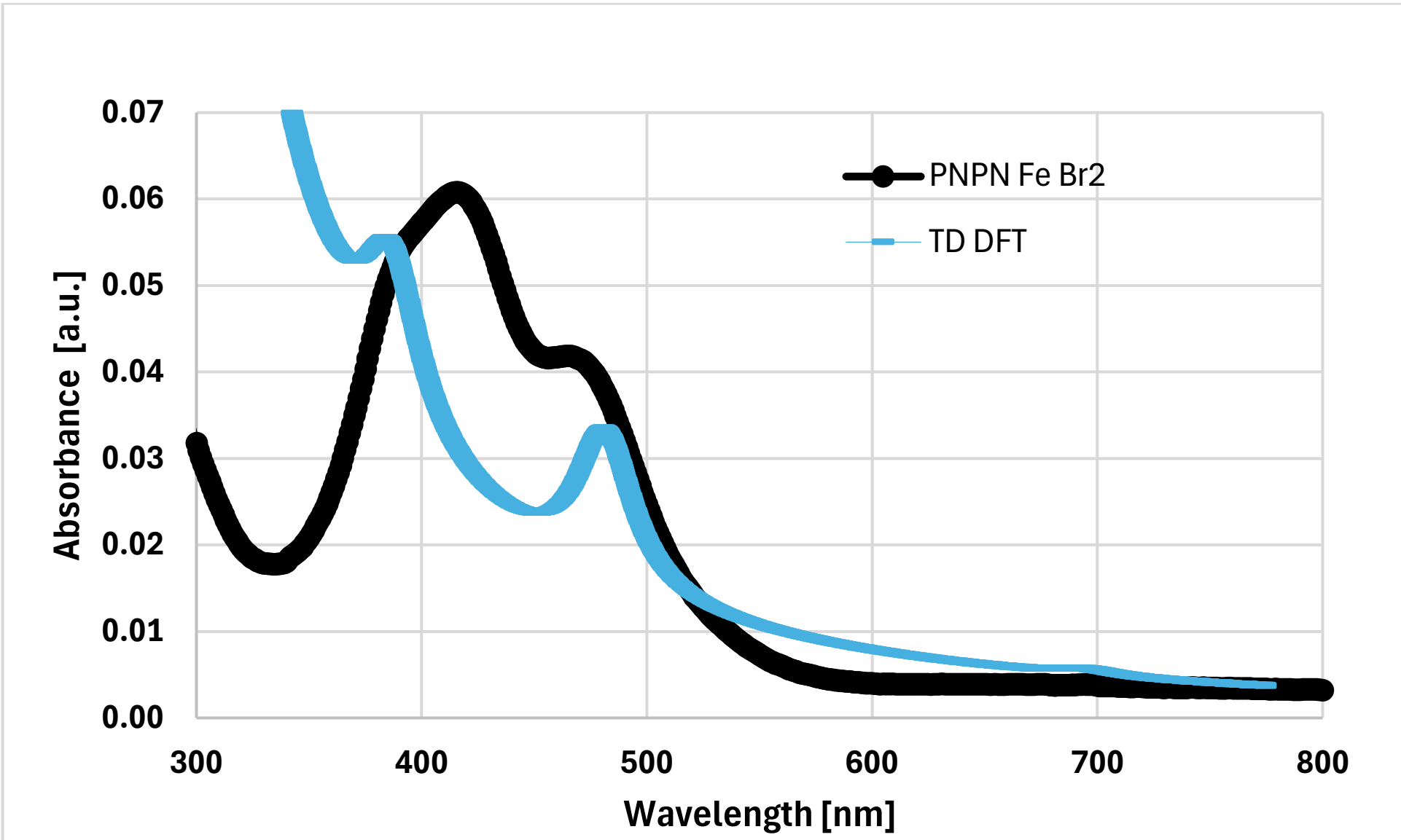

Figure S 3. UV/Vis spectrum of 1-Br in THF (ca. 0.1 mM) at 23°C (black) and TD-DFT spectrum (blue).

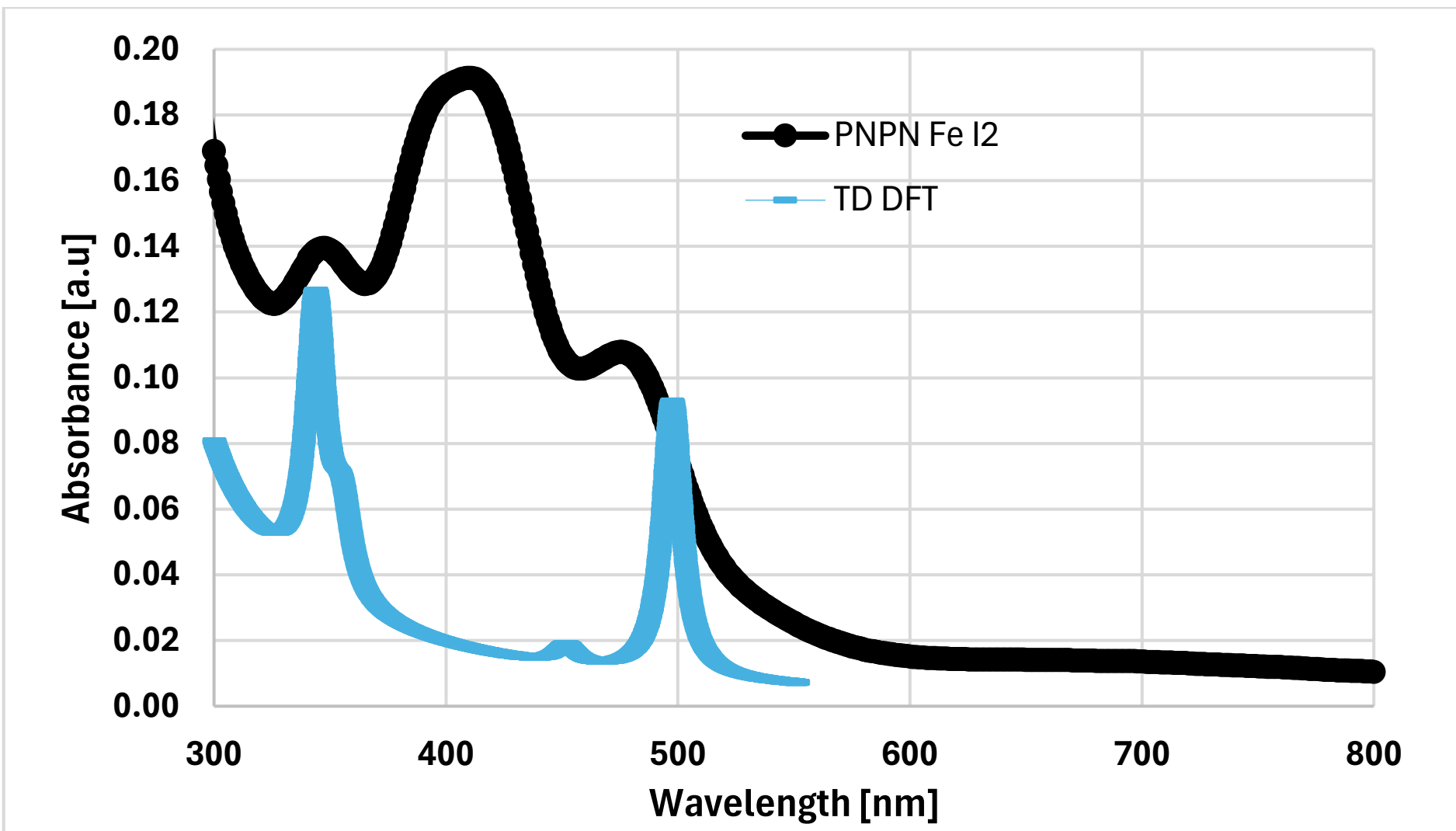

Figure S 4. UV/Vis spectrum of 1-I in THF (ca. 0.1 mM) at 23°C (black) and TD-DFT spectrum (blue).

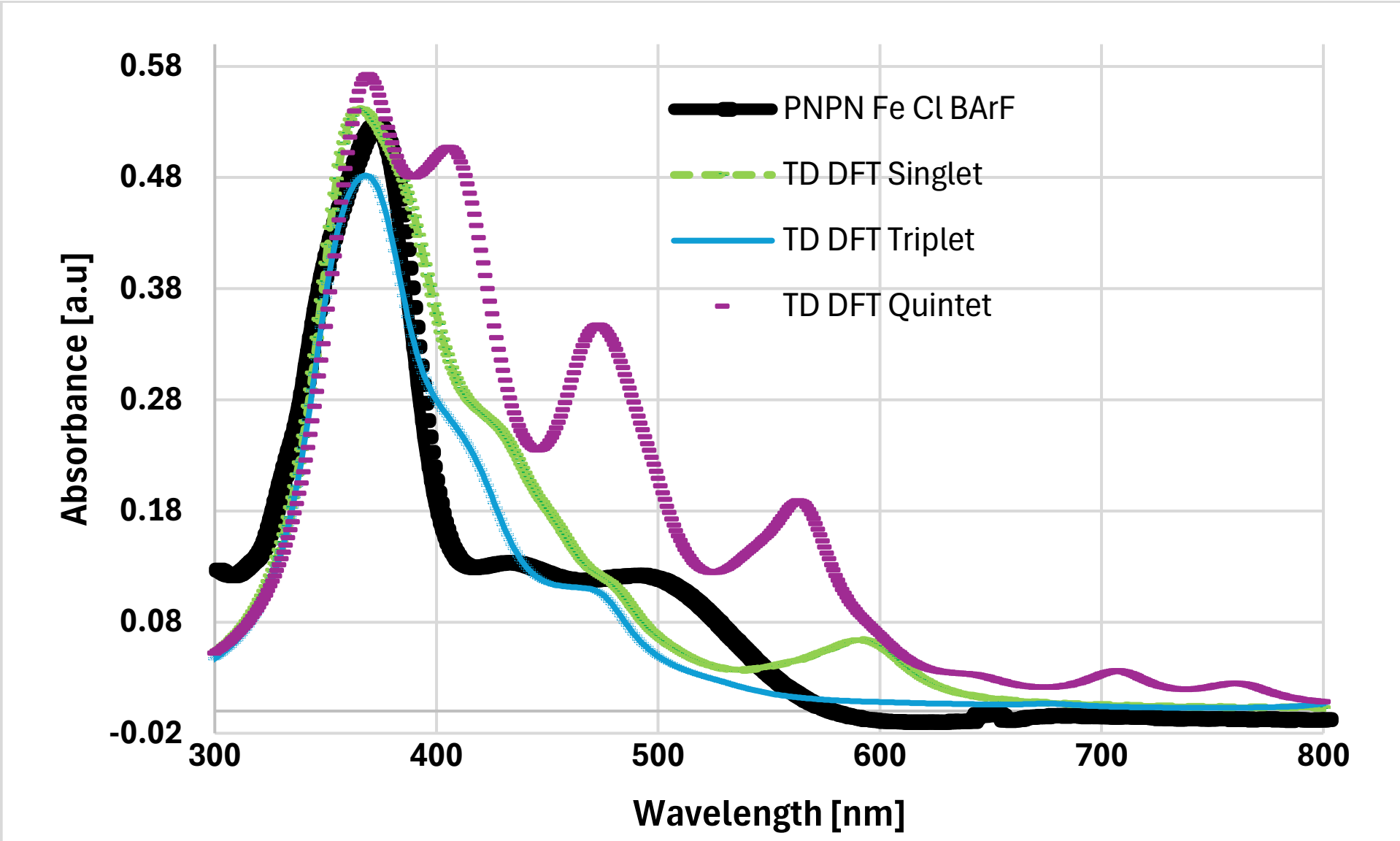

Figure S 5. UV/Vis spectrum of 2-Cl in THF (ca. 0.1 mM) at 23°C (black) and TD-DFT spectrum of the singlet species (green), TD-DFT spectrum of the triplet species (blue), and TD-DFT spectrum of the quintet species (purple).

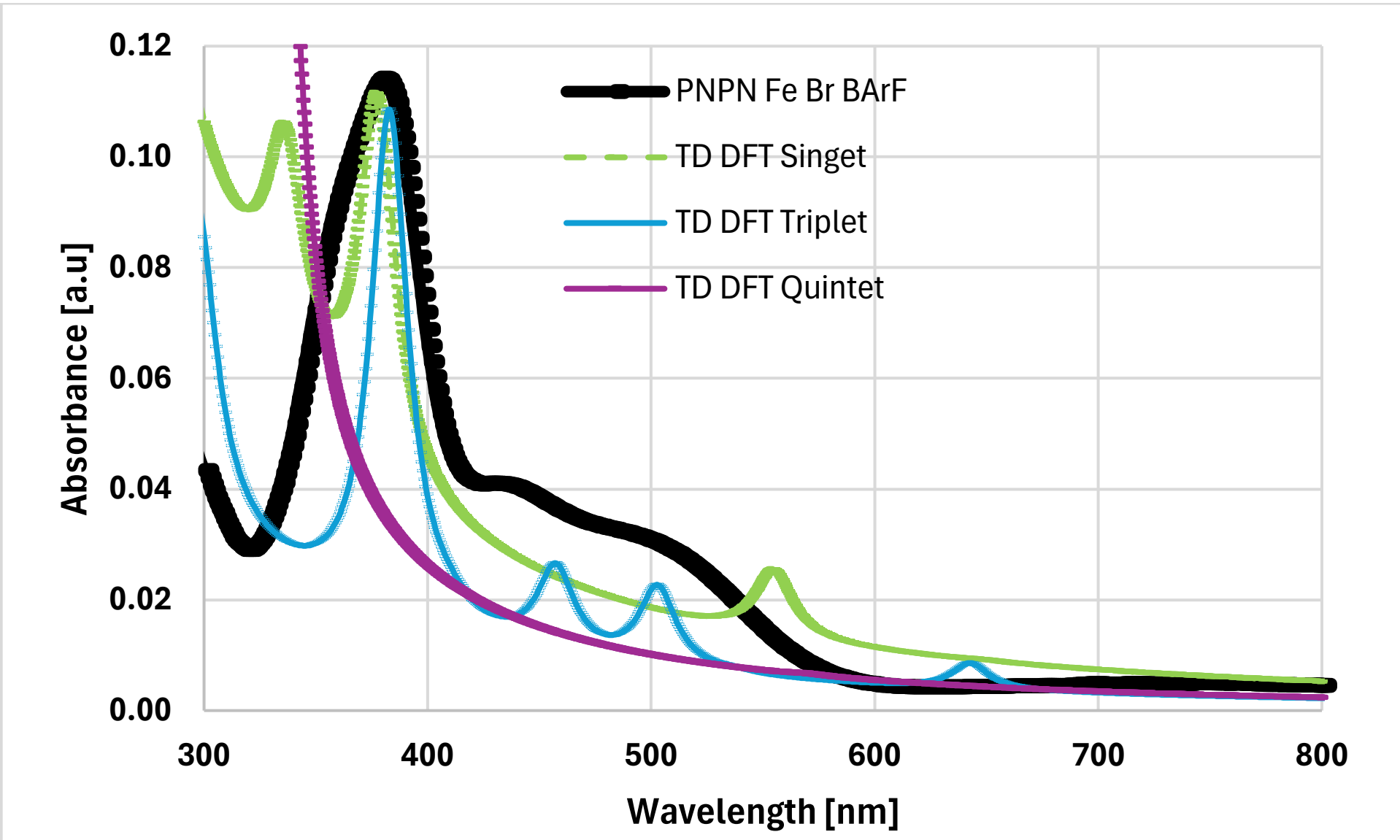

Figure S 6. UV/Vis spectrum of 2-Br in THF (ca. 0.1 mM) at 23°C (black) and TD-DFT spectrum of the singlet species (green), TD-DFT spectrum of the triplet species (blue), and TD-DFT spectrum of the quintet species (purple).

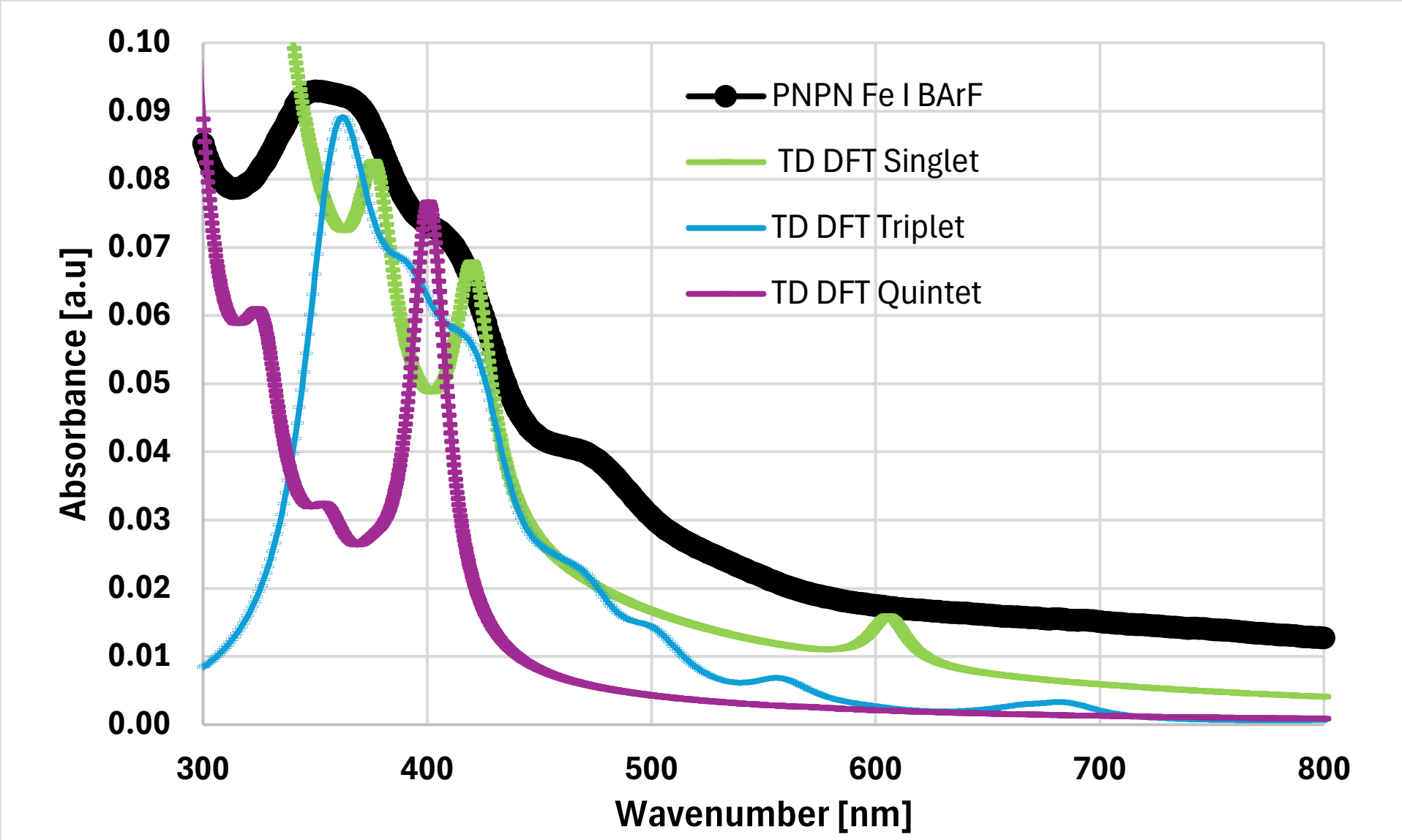

Figure S 7. UV/Vis spectrum of 2-I in DCM (ca. 0.1 mM) at 23°C (black) and TD-DFT spectrum of the singlet species (green), TD-DFT spectrum of the triplet species (blue), and TD-DFT spectrum of the quintet species (purple).

4.3 Correlations of ^{15}N-shifts with Structural Parameters

Table S6 contains calculated parameters and measured ^{15}N-shifts for complexes **1**, **3**, and **4**. We also plotted various combinations of calculated parameters with the detected ^{15}N-shifts. In fact, we could only find a reasonable correlation for Figure S10 (Hirshfeld Fe vs.^{15}N-shifts of pyridine *trans* Br) and Figure S15 (TEP vs. ^{15}N-shifts of pyridine donor *trans* L)

*Table S 6. ^{15}N-shifts of complexes **1**, **3**, and **4** and calculated Hirshfeld charges and TEP on TPSSh/ def2-TZVPP/D4 level.*

Compound	^{15}N shift trans halide [ppm]	^{15}N shift trans L [ppm]	Fe-X bond length [Å]	Fe-L bond length [Å]	Hirshfeld Charge Fe	Hirshfeld Charge N *trans* Br	Hirshfeld Charge N *trans* L	TEP [cm^{-1}]
**1-Cl**	205.4	-	1.917	1.917	-0.065	-0.127	-0.127	-
**1-Br**	215.2	-	1.975	1.975	-0.089	-0.109	-0.109	-
**1-I**	221.7	-	1.895	1.895	-0.086	-0.118	-0.118	-
**3-MeCN**	208.1	195.3	1.99	2.01	-0.116	-0.116	-0.110	2118
**3-Pyr**	202.6	199.1	1.96	1.982	-0.116	-0.116	-0.111	2110
**3-PPh$_3$**	215.3	205.3	1.966	1.999	-0.113	-0.113	-0.116	2107
**3-PMe$_3$**	218.2	200.5	2.017	2.129	-0.111	-0.111	-0.115	2105
**3-tBuNC**	215.4	204.1	1.978	1.997	-0.113	-0.113	-0.111	2112
**3-CO**	215.6	188.6	1.956	1.986	-0.114	-0.114	-0.112	2165
**4**	-	189.5	-	2.002	0.008	-	-0.126	2118

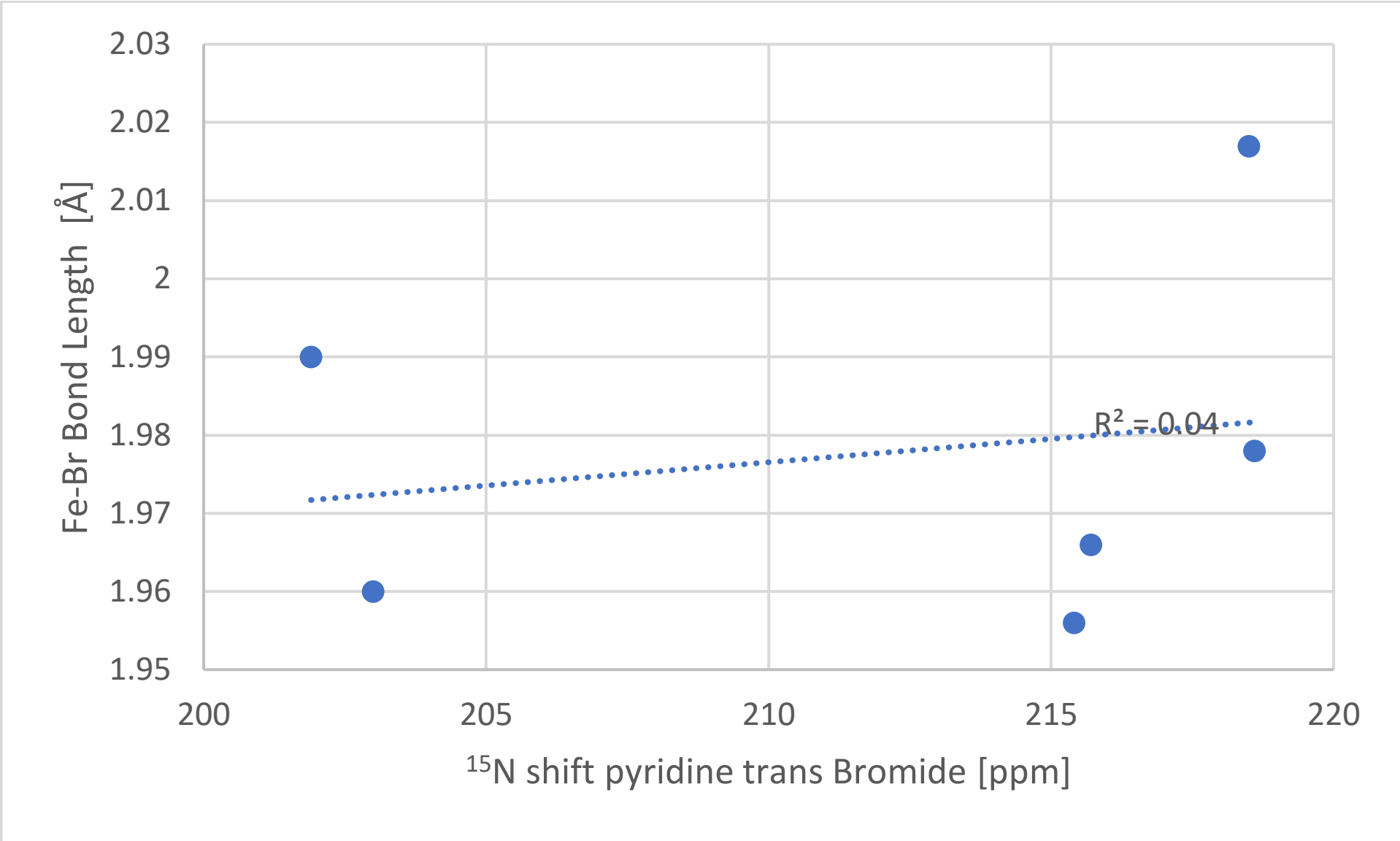

*Figure S 8. Depiction of Potential Correction of ^{15}N shift pyridine trans Bromide with Fe-Br Bond Length within Series of Complexes **3**.*

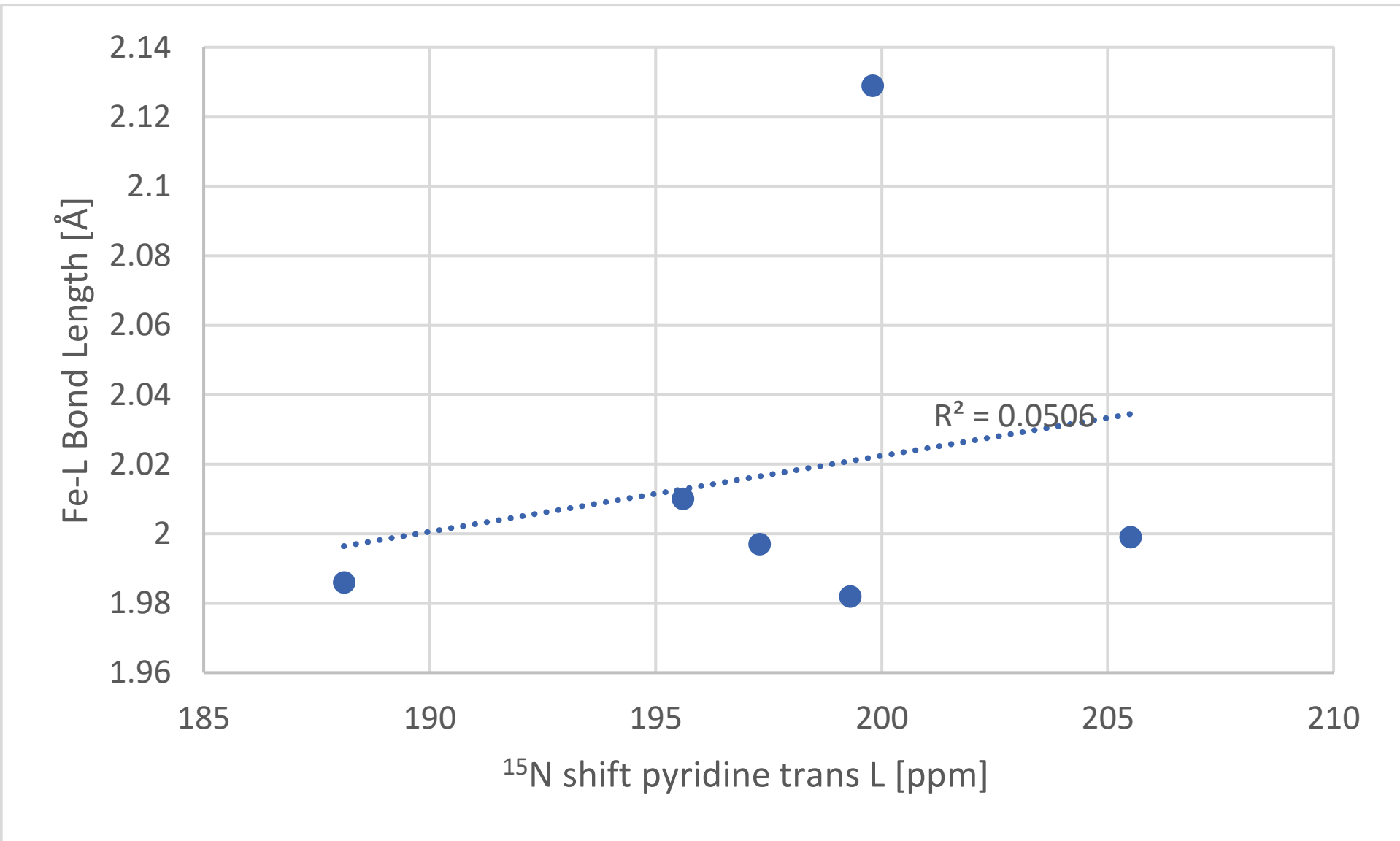

*Figure S 9. Depiction of Potential Correction of ^{15}N shift pyridine trans L with Fe-L Bond Length within Series of Complexes **3**.*

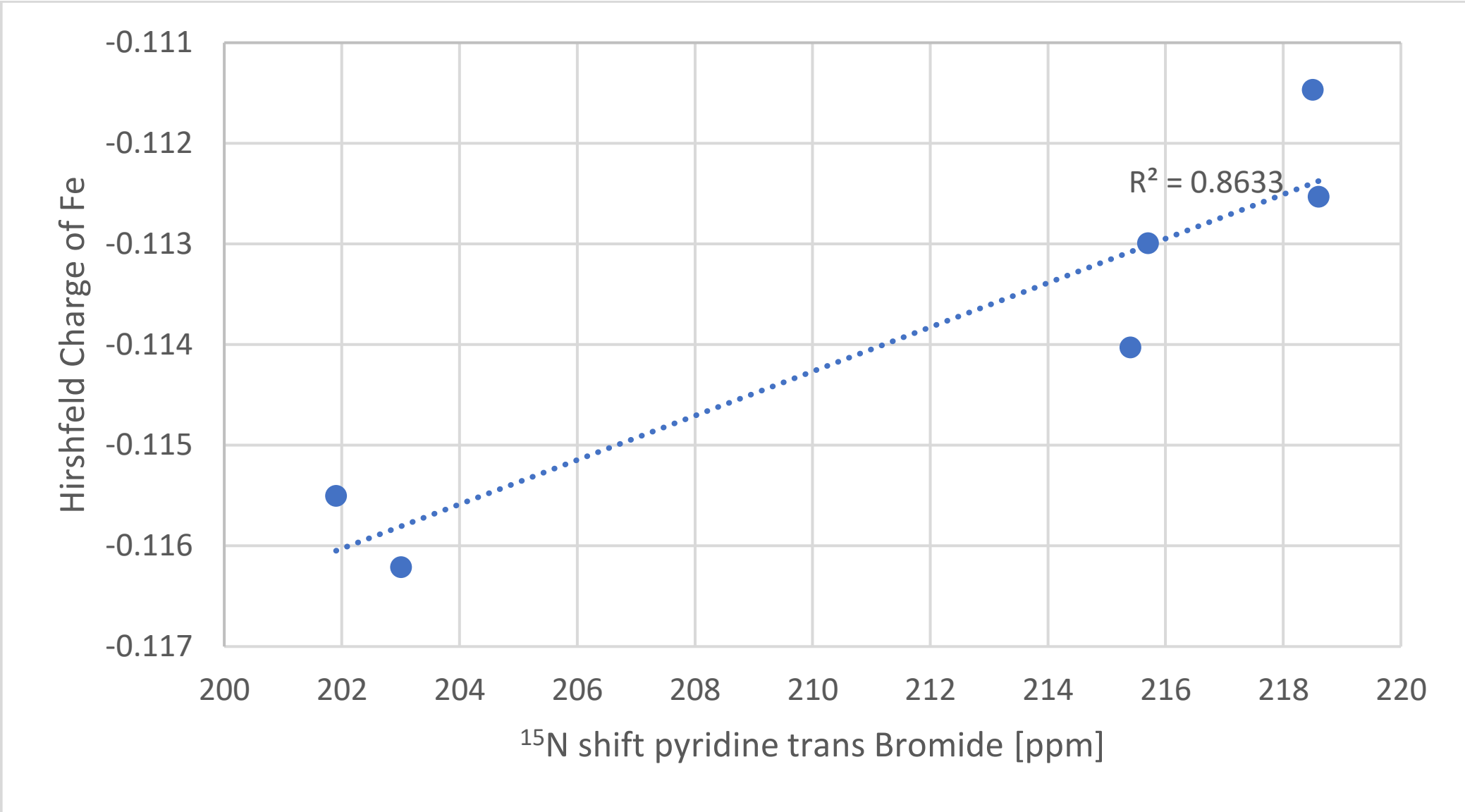

*Figure S 10. Depiction of Potential Correction of ^{15}N shift pyridine trans Bromide with Hirshfeld Charge on Fe within Series of Complexes **3**.*

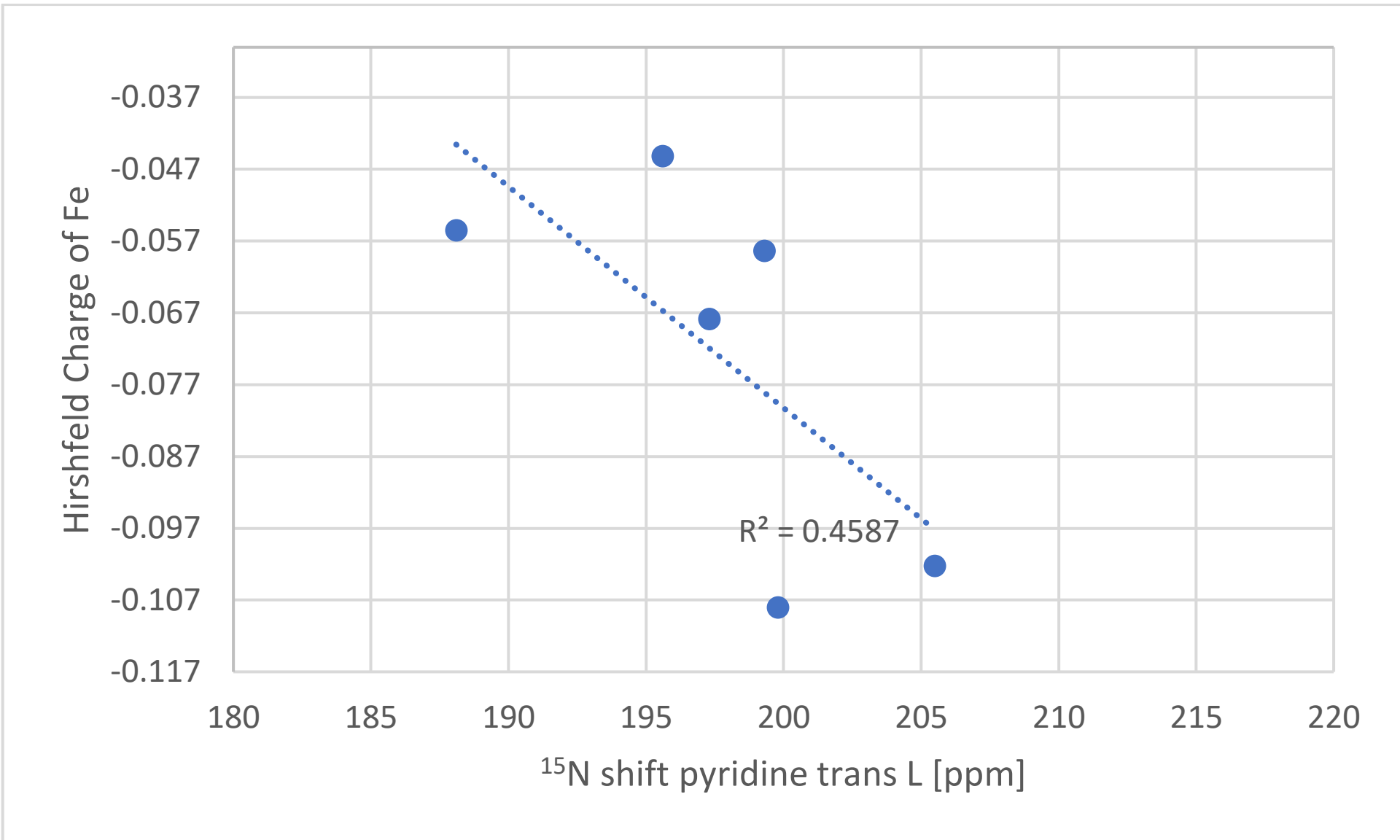

*Figure S 11. Depiction of Potential Correction of ^{15}N shift pyridine trans L with Hirshfeld Charge on Fe within Series of Complexes **3**.*

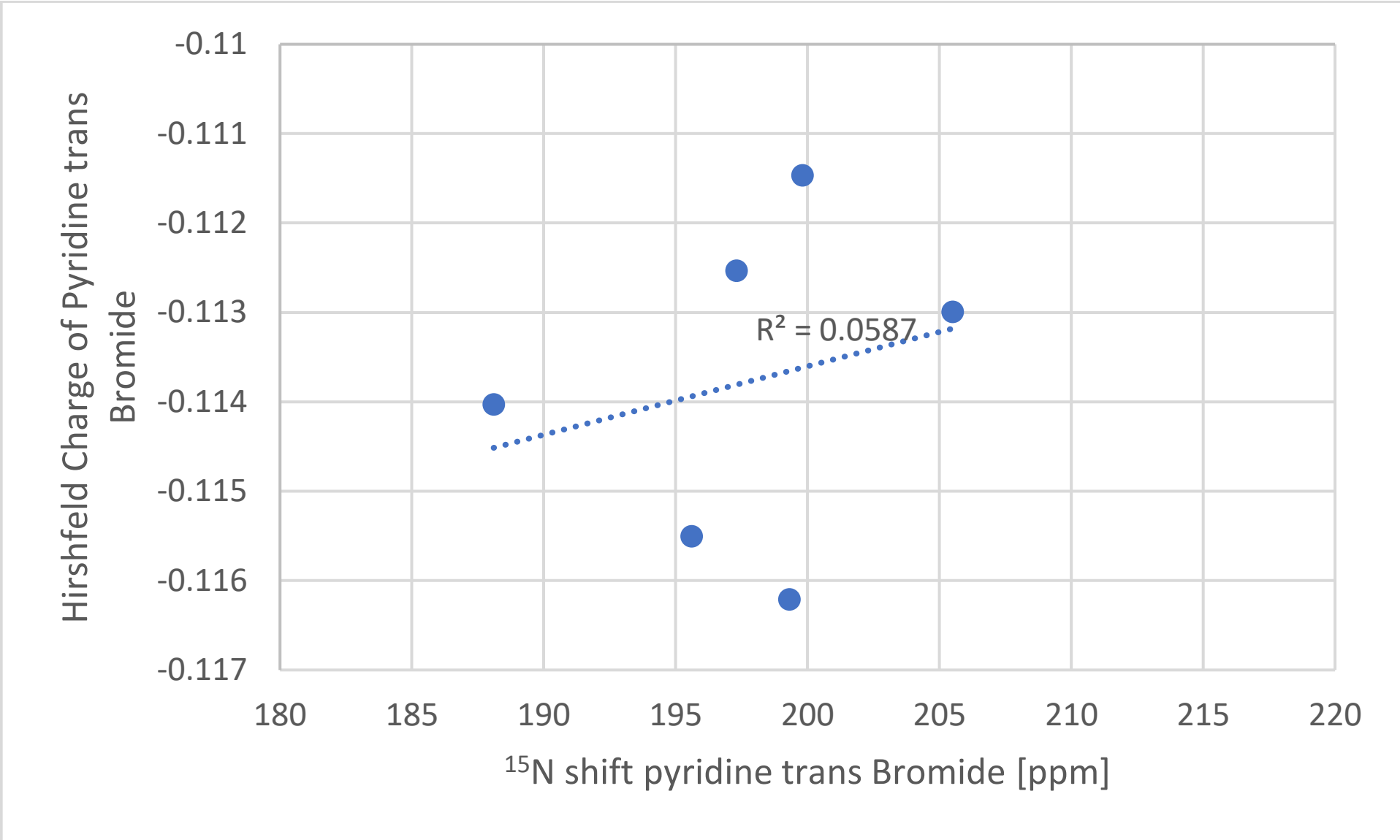

*Figure S 12. Depiction of Potential Correction of ^{15}N shift pyridine trans Bromide with Hirshfeld Charge on Pyridine trans Bromide within Series of Complexes **3**.*

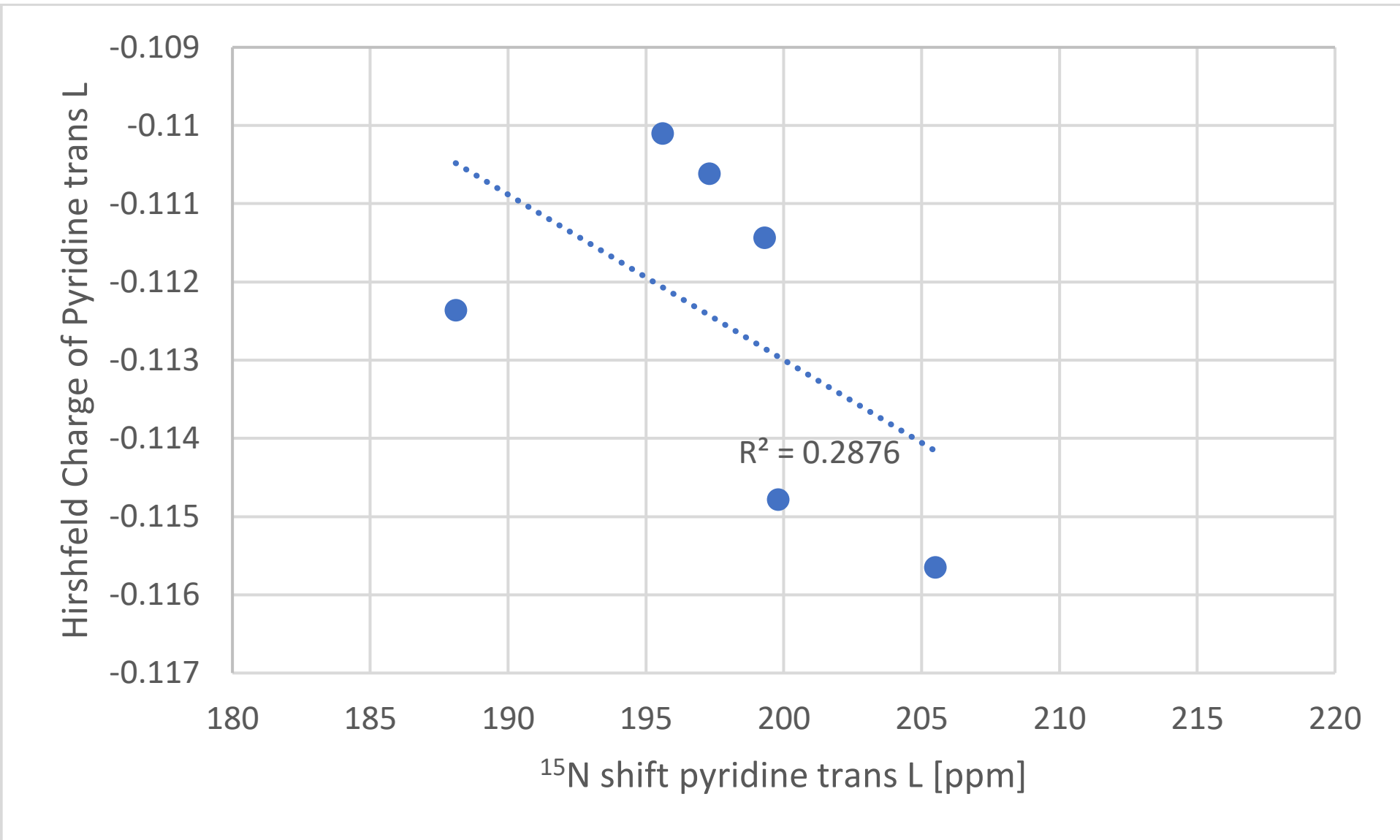

*Figure S 13. Depiction of Potential Correction of ^{15}N shift pyridine trans L with Hirshfeld Charge on Pyridine trans L within Series of Complexes **3**.*

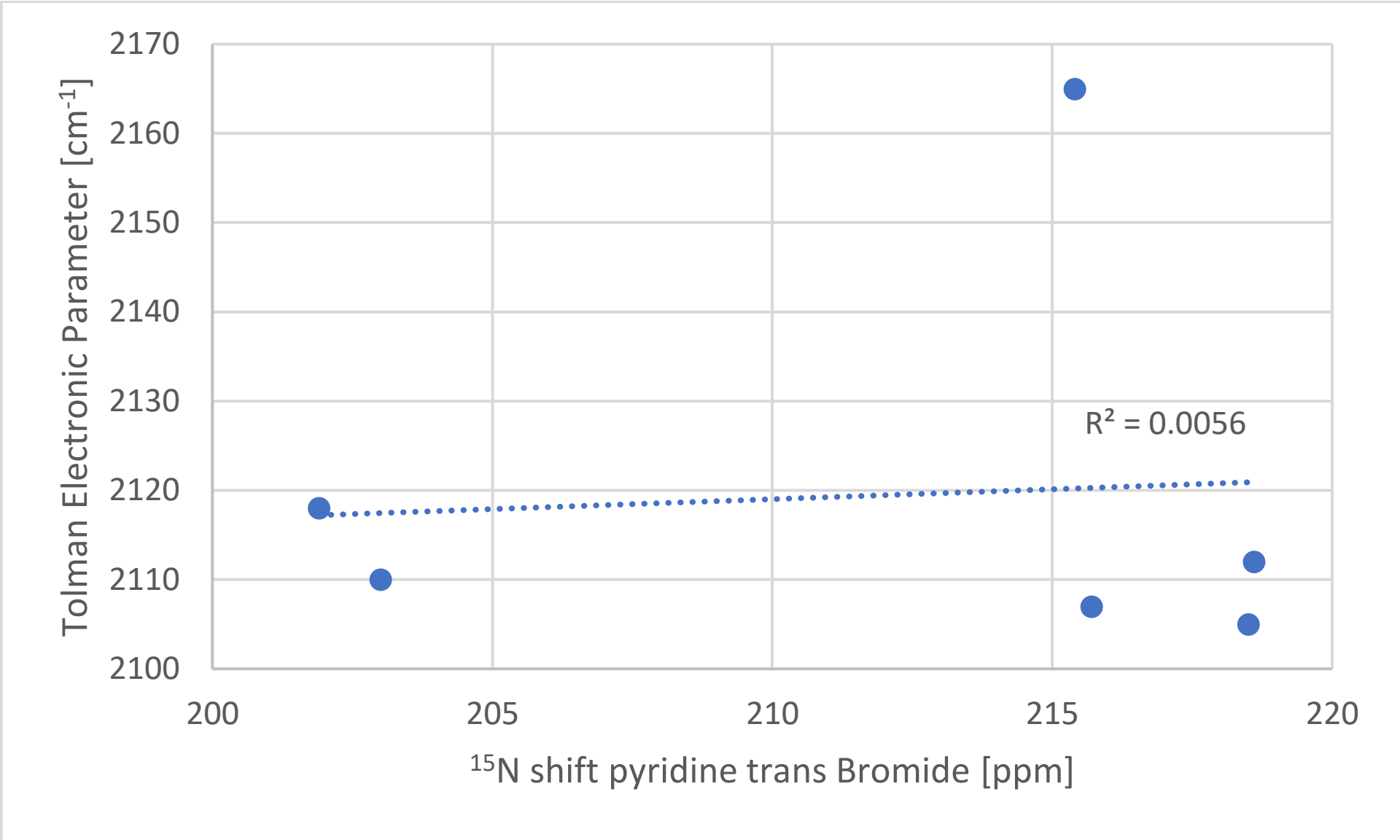

Figure S 14. Depiction of Potential Correction of ^{15}N shift pyridine trans Bromide with TEP of L within Series of Complexes ***3***.

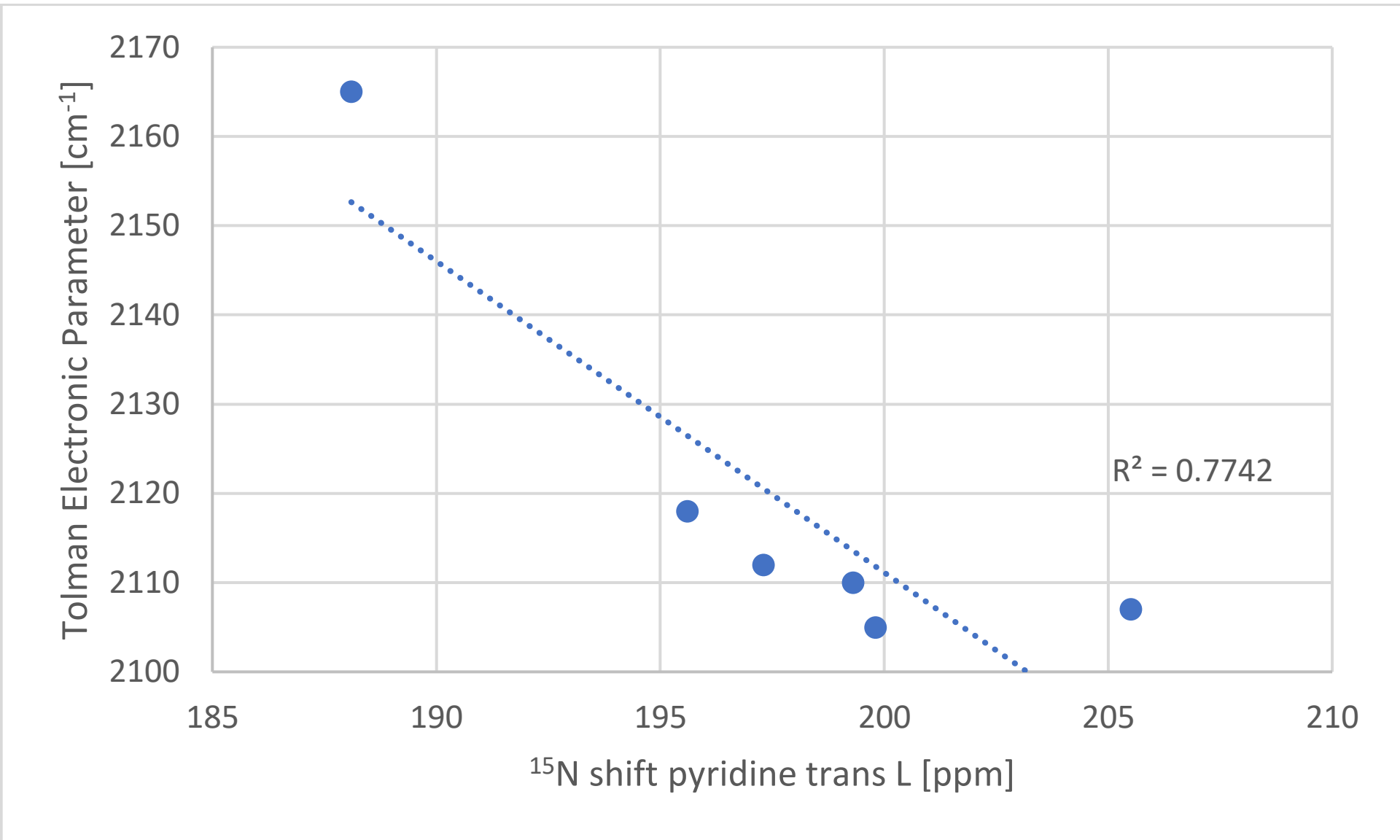

Figure S 15. Depiction of Potential Correction of ^{15}N shift pyridine trans L with TEP of L within Series of Complexes ***3***.

5 NMR Spectra

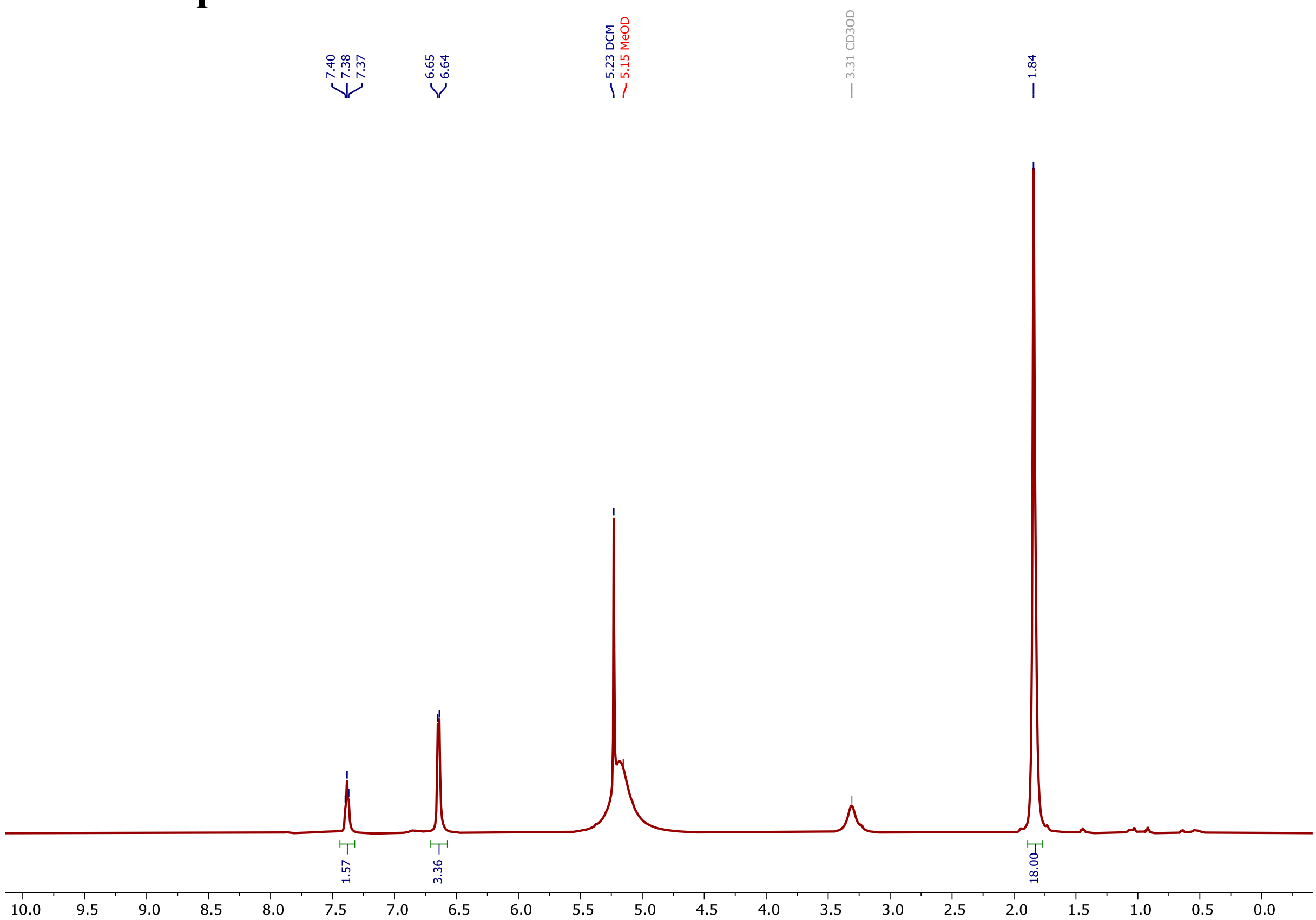

*Figure S 16. ^{1}H-NMR spectrum (23°C, 600 MHz) of **1-Cl** in MeOD-d$_4$.*

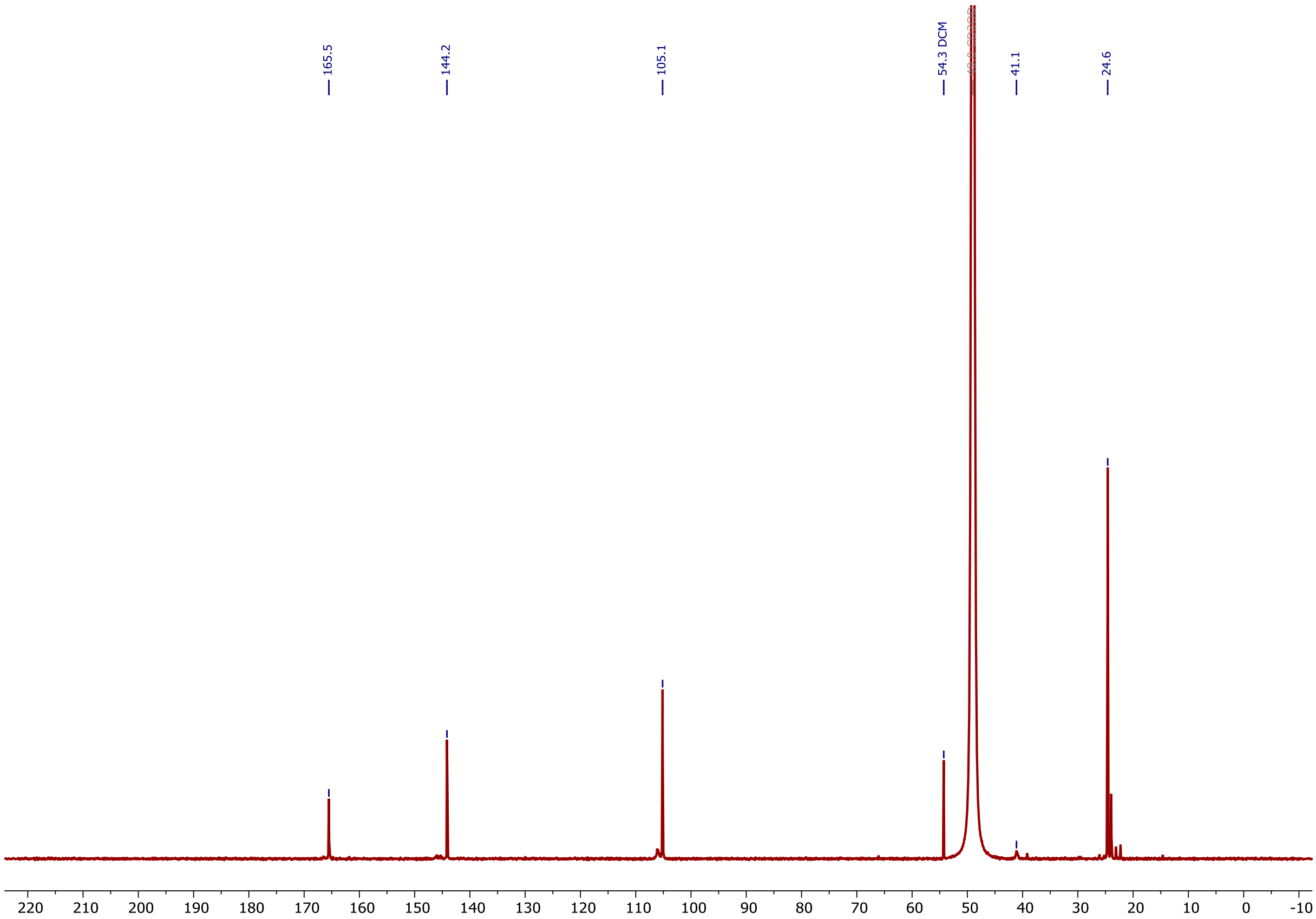

*Figure S 17. ^{13}C{^{1}H}-NMR spectrum (23°C, 150 MHz) of **1-Cl** in MeOD-d$_4$.*

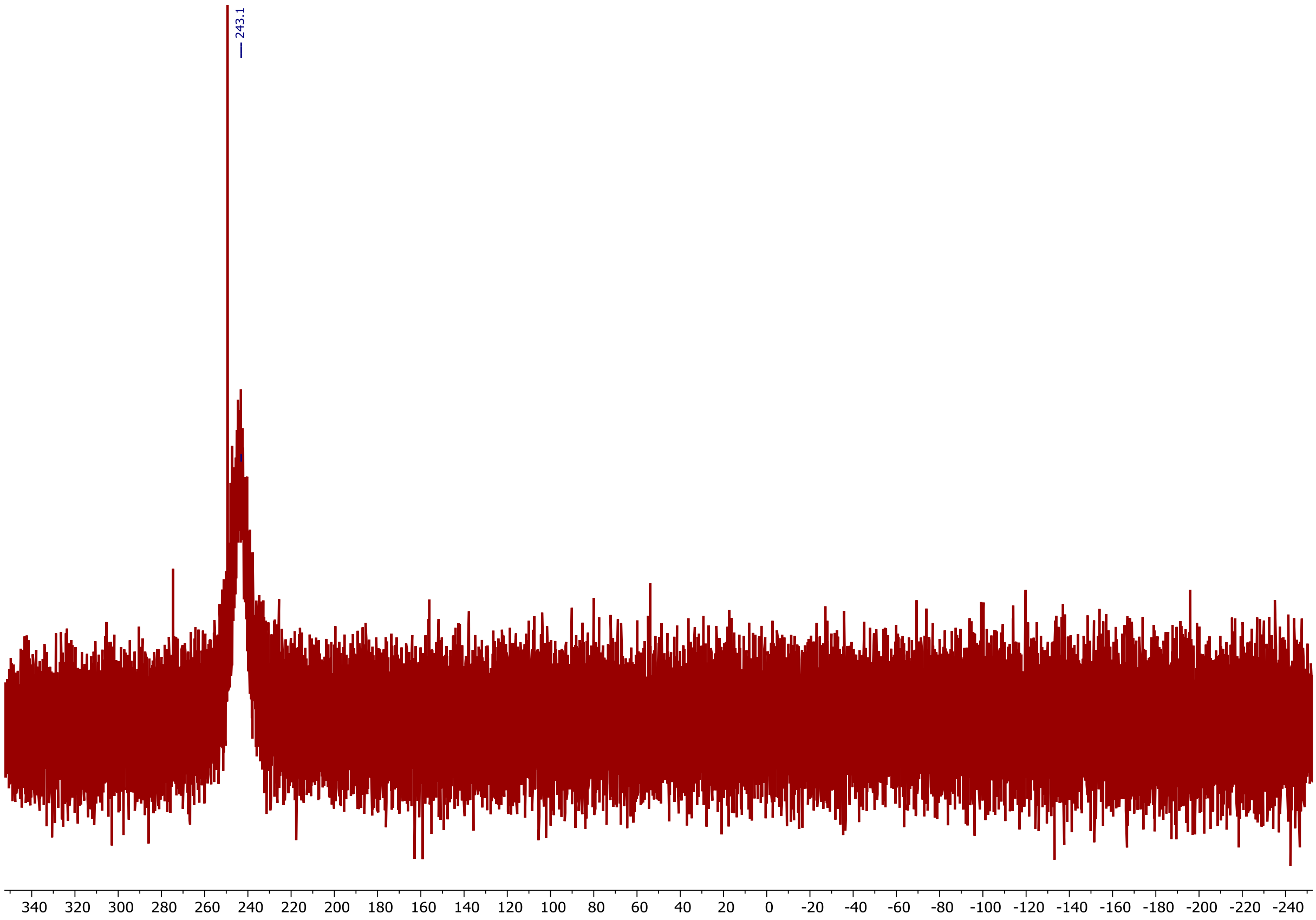

Figure S 18. $^{31}P\{^{1}H\}$*-NMR spectrum (23°C, 241 MHz) of* ***1-Cl*** *in MeOD-d*$_4$*.*

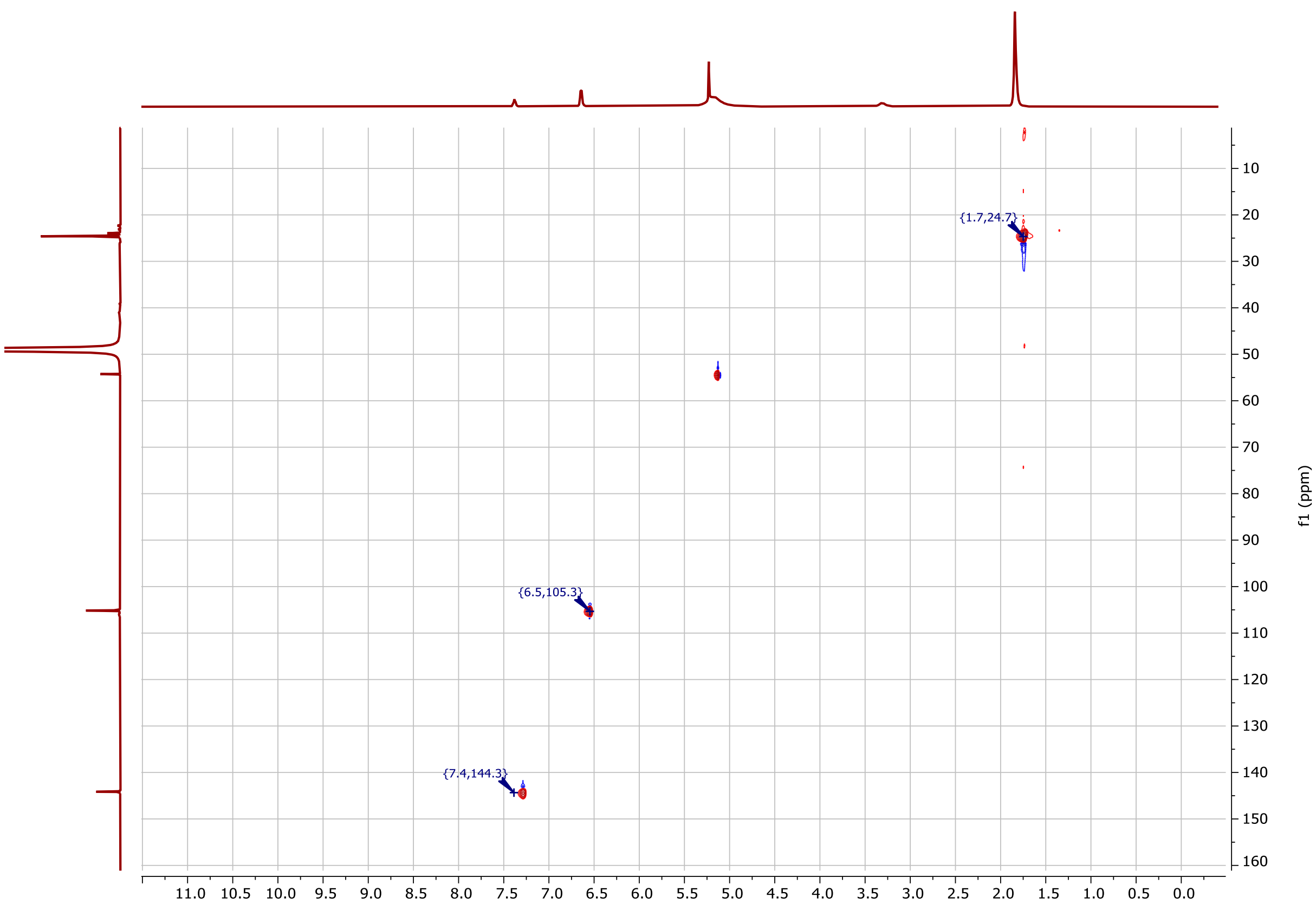

Figure S 19. $^{1}H/^{13}C$*-HSQC spectrum (23°C) of* ***1-Cl*** *in MeOD-d*$_4$*.*

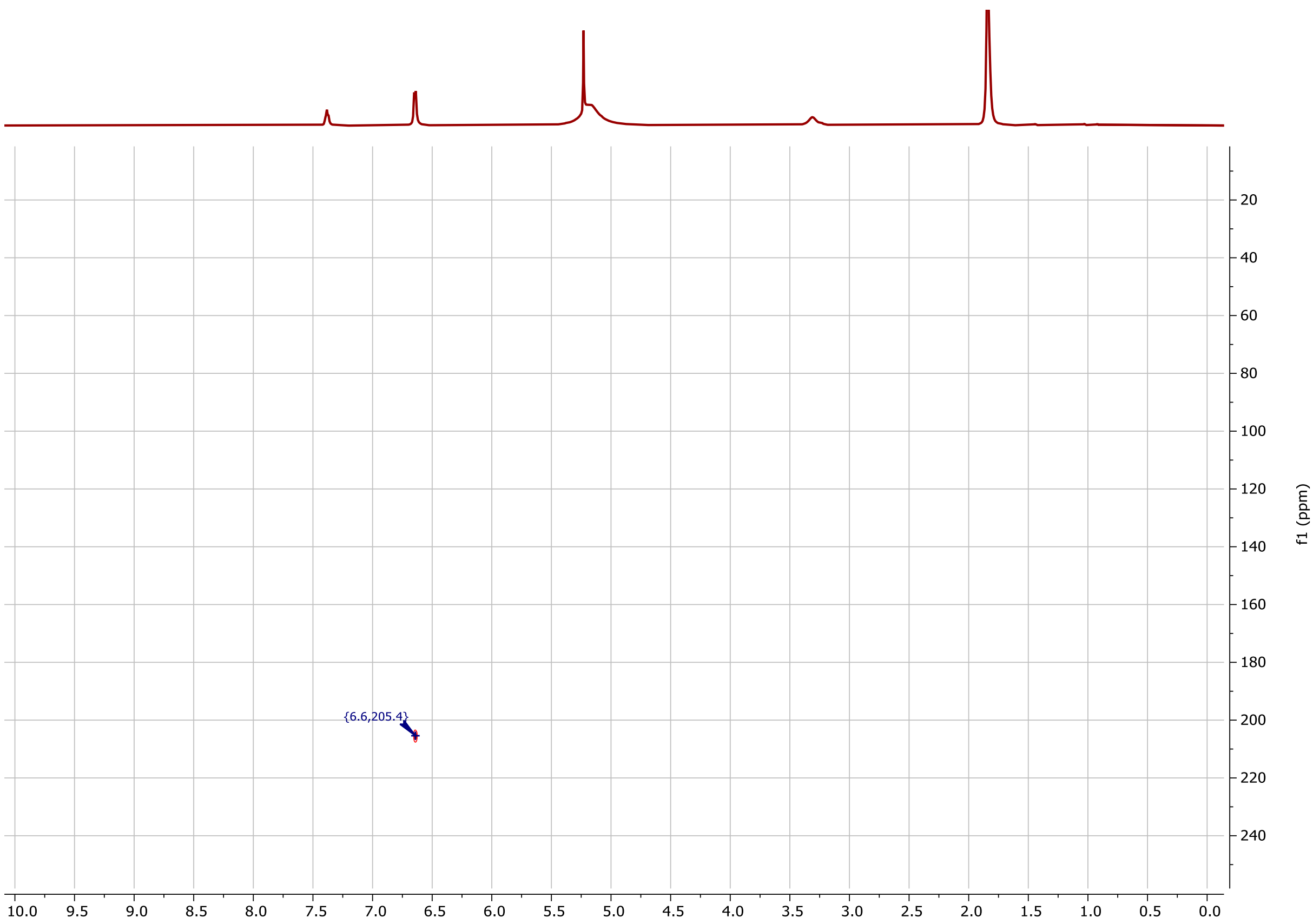

*Figure S 20. $^{1}H/^{15}N$-HMBC spectrum (23°C) of **1-Cl** in MeOD-d_4.*

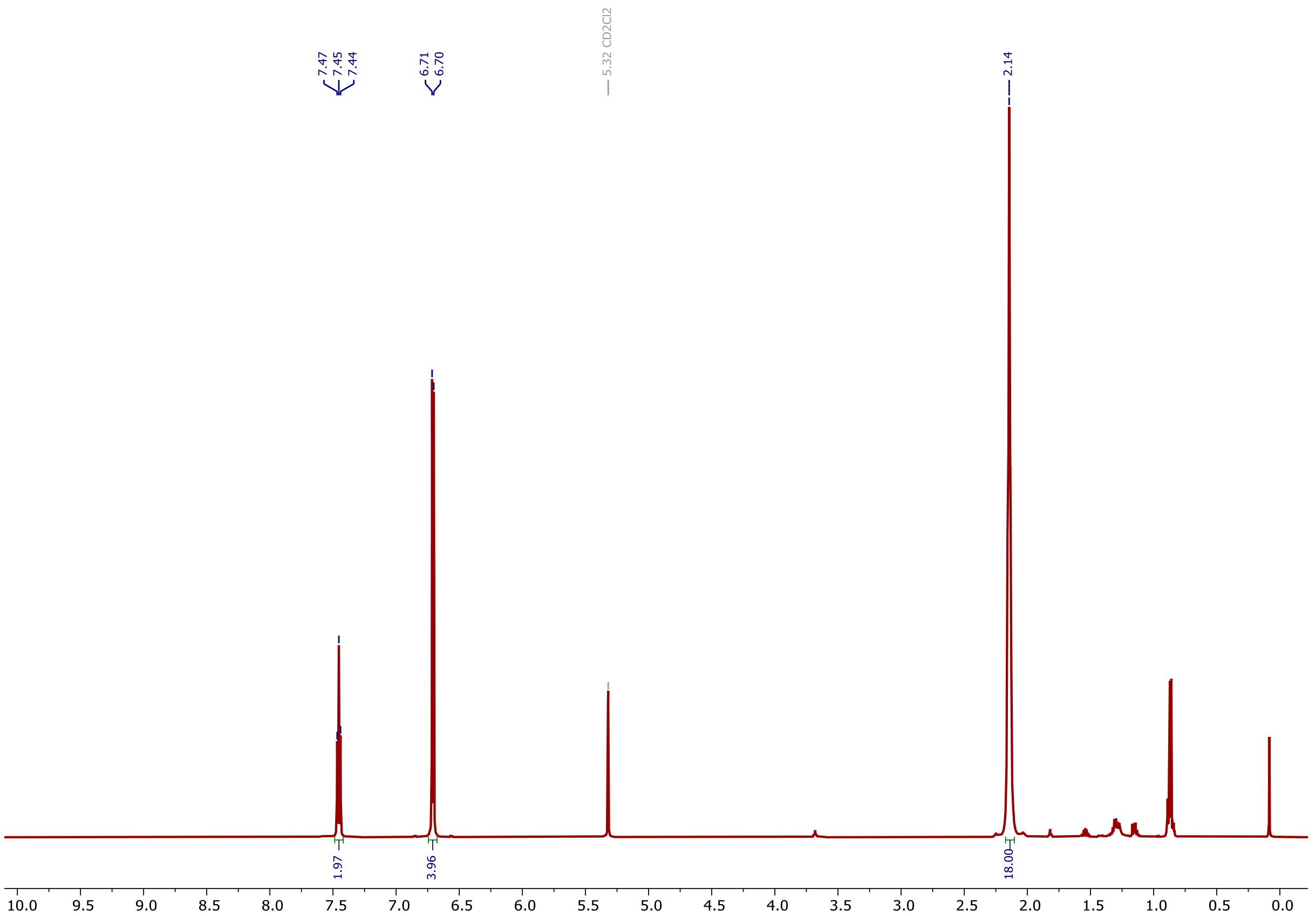

*Figure S 21. ^{1}H-NMR spectrum (23°C, 600 MHz) of **1-Br** in DCM-d_2.*

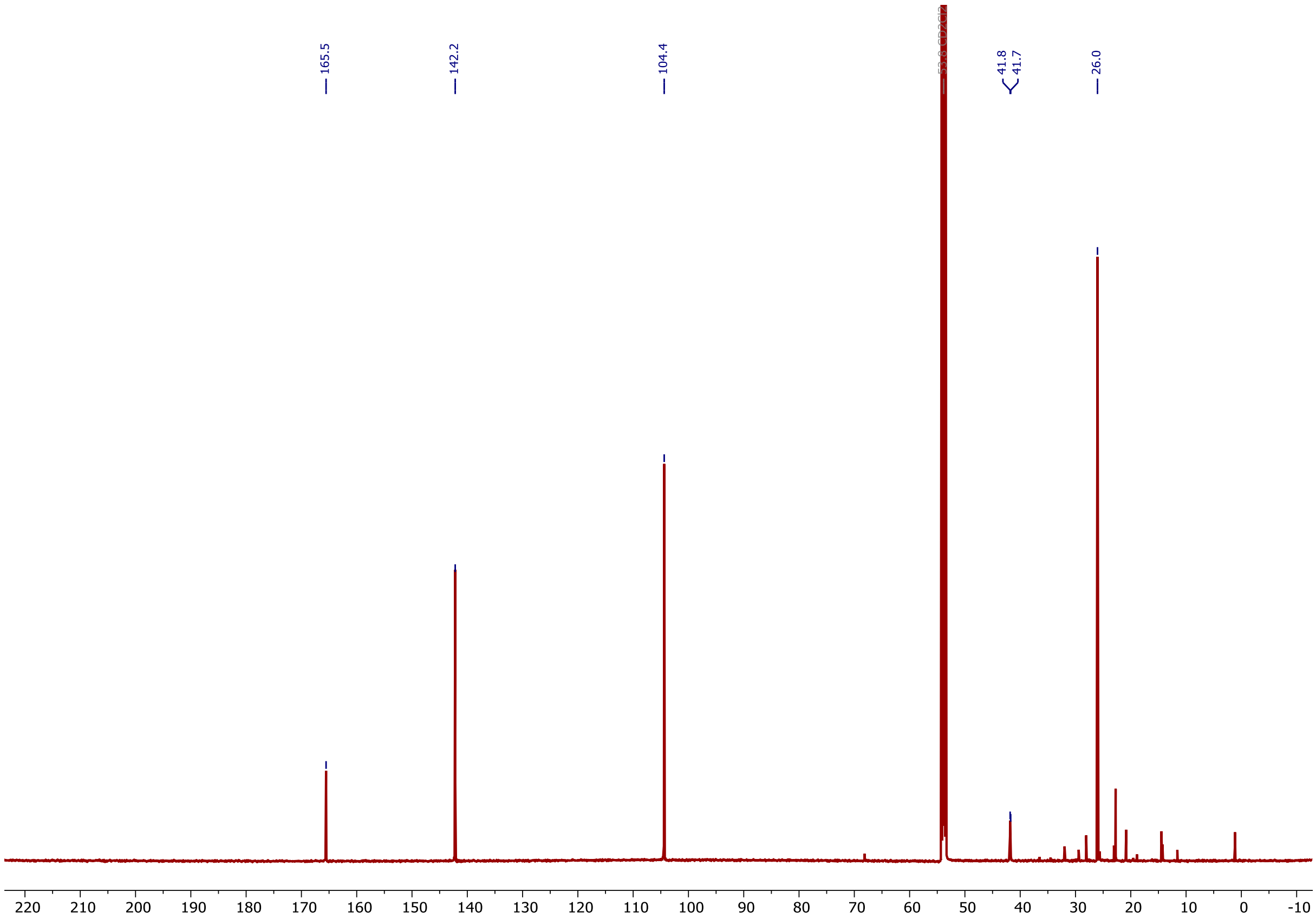

*Figure S 22. $^{13}C\{^{1}H\}$-NMR spectrum (23°C, 150 MHz) of **1-Cl** in DCM-d_2.*

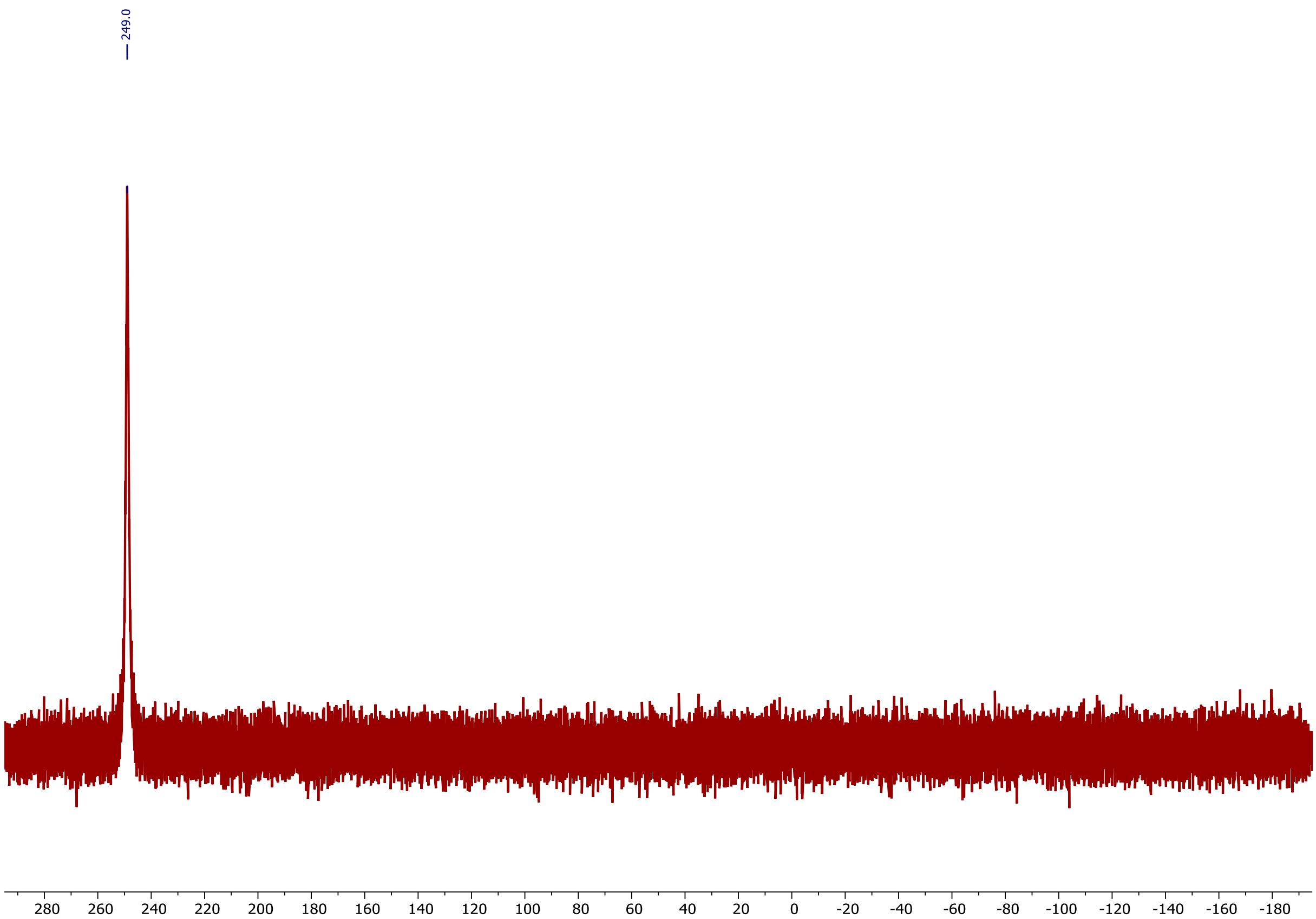

*Figure S 23. $^{31}P\{^{1}H\}$-NMR spectrum (23°C, 241 MHz) of **1-Br** in DCM-d_2.*

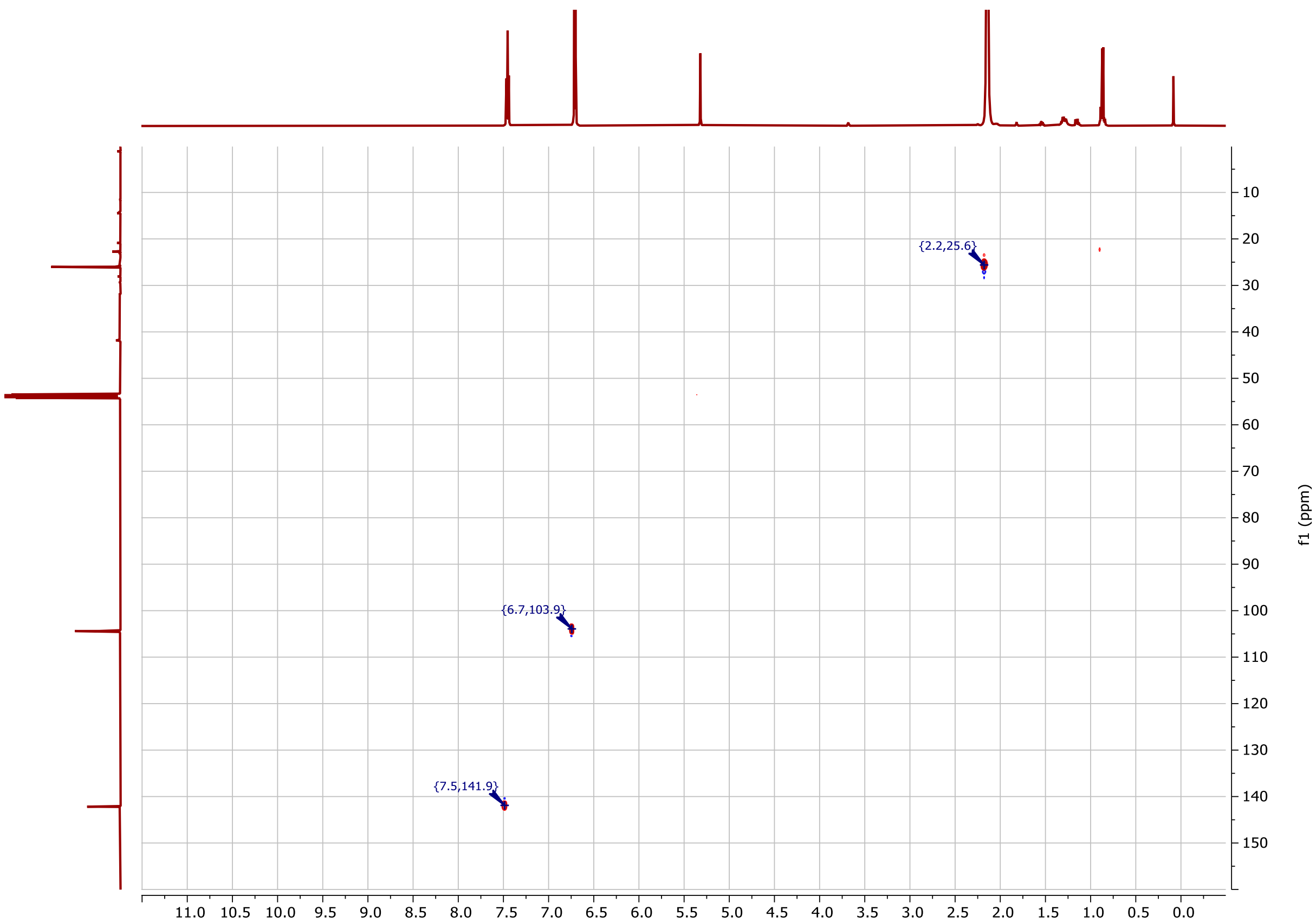

*Figure S 24. $^{1}H/^{13}C$-HSQC spectrum (23°C) of **1-Br** in DCM-d_2.*

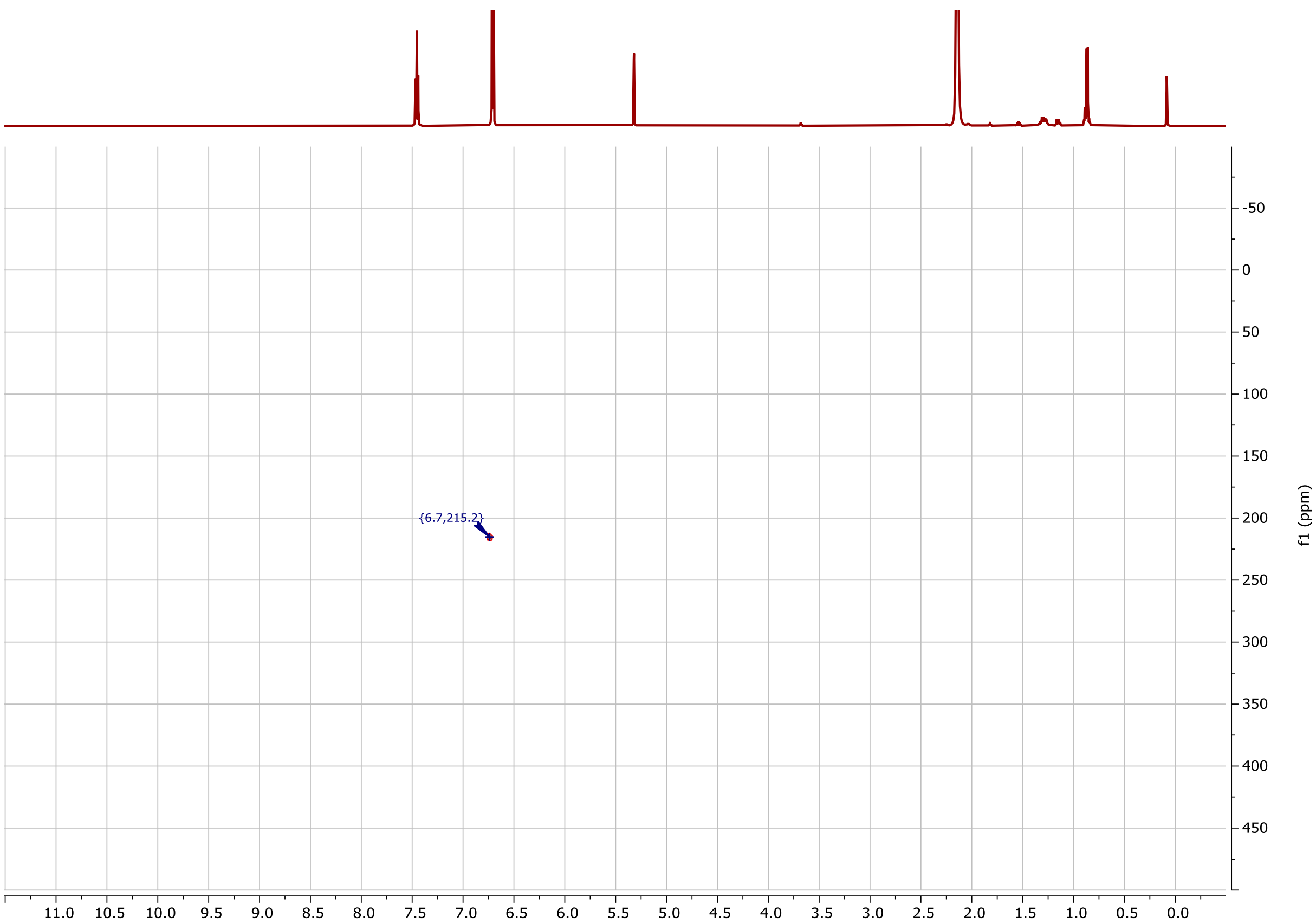

*Figure S 25. $^{1}H/^{15}N$-HMBC spectrum (23°C) of **1-Br** in DCM-$_{d2}$.*

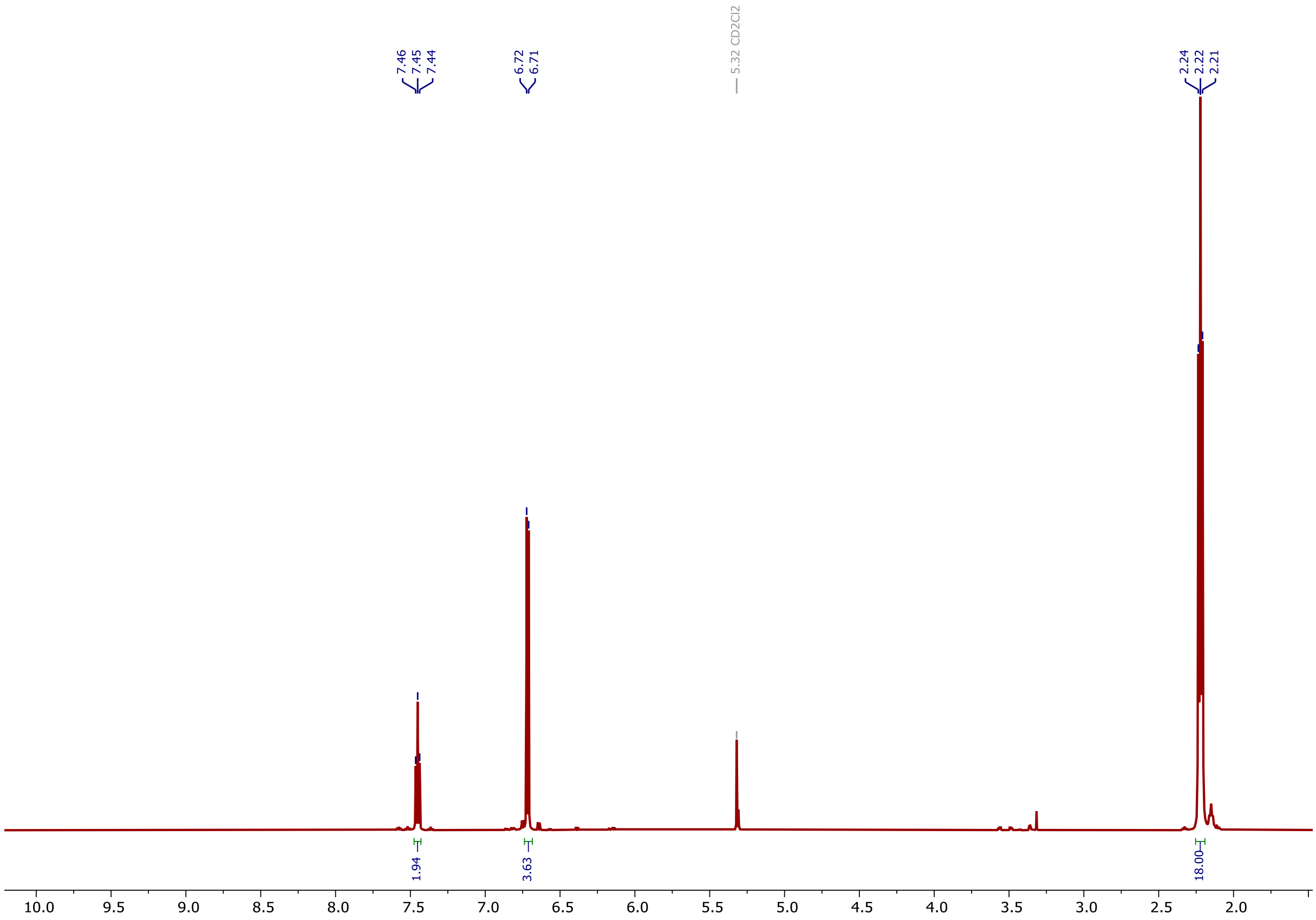

*Figure S 26. ^{1}H-NMR spectrum (23°C, 600 MHz) of **1-I** in DCM-d_2.*

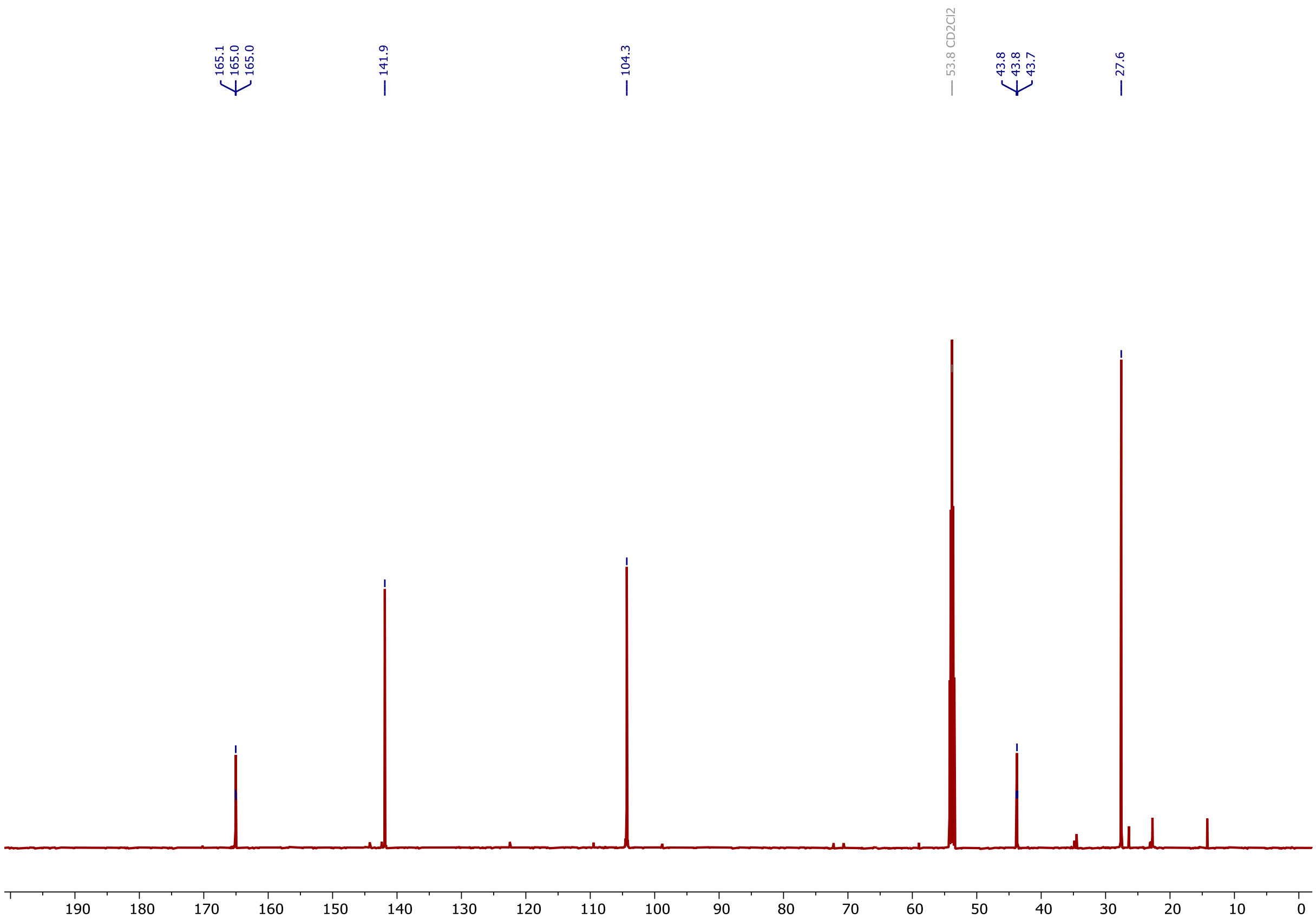

*Figure S 27. ^{13}C{^{1}H}-NMR spectrum (23°C, 150 MHz) of **1-I** in DCM-d_2.*

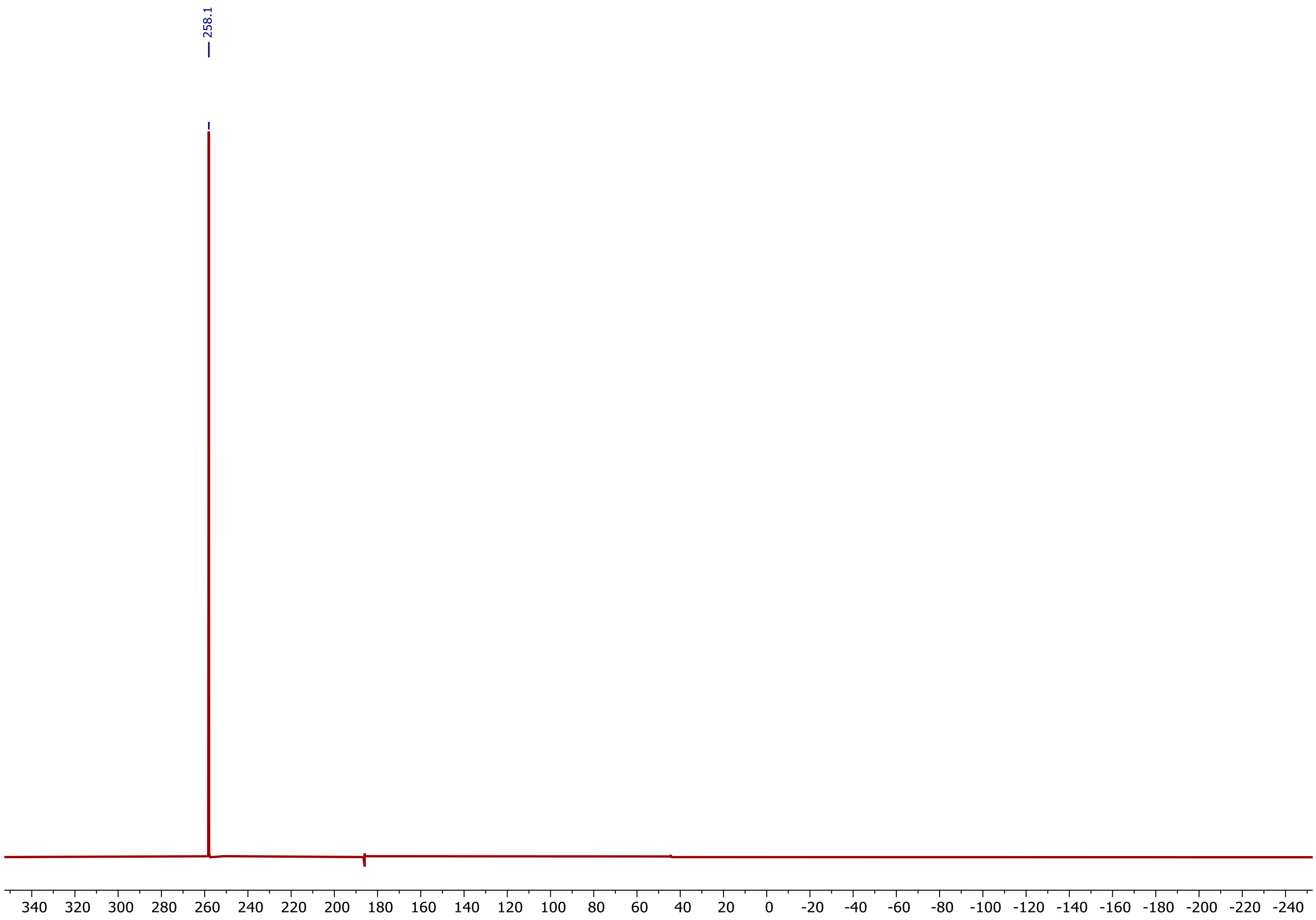

*Figure S 28. $^{31}P\{^{1}H\}$-NMR spectrum (23°C, 241 MHz) of **1-I** in DCM-d$_2$.*

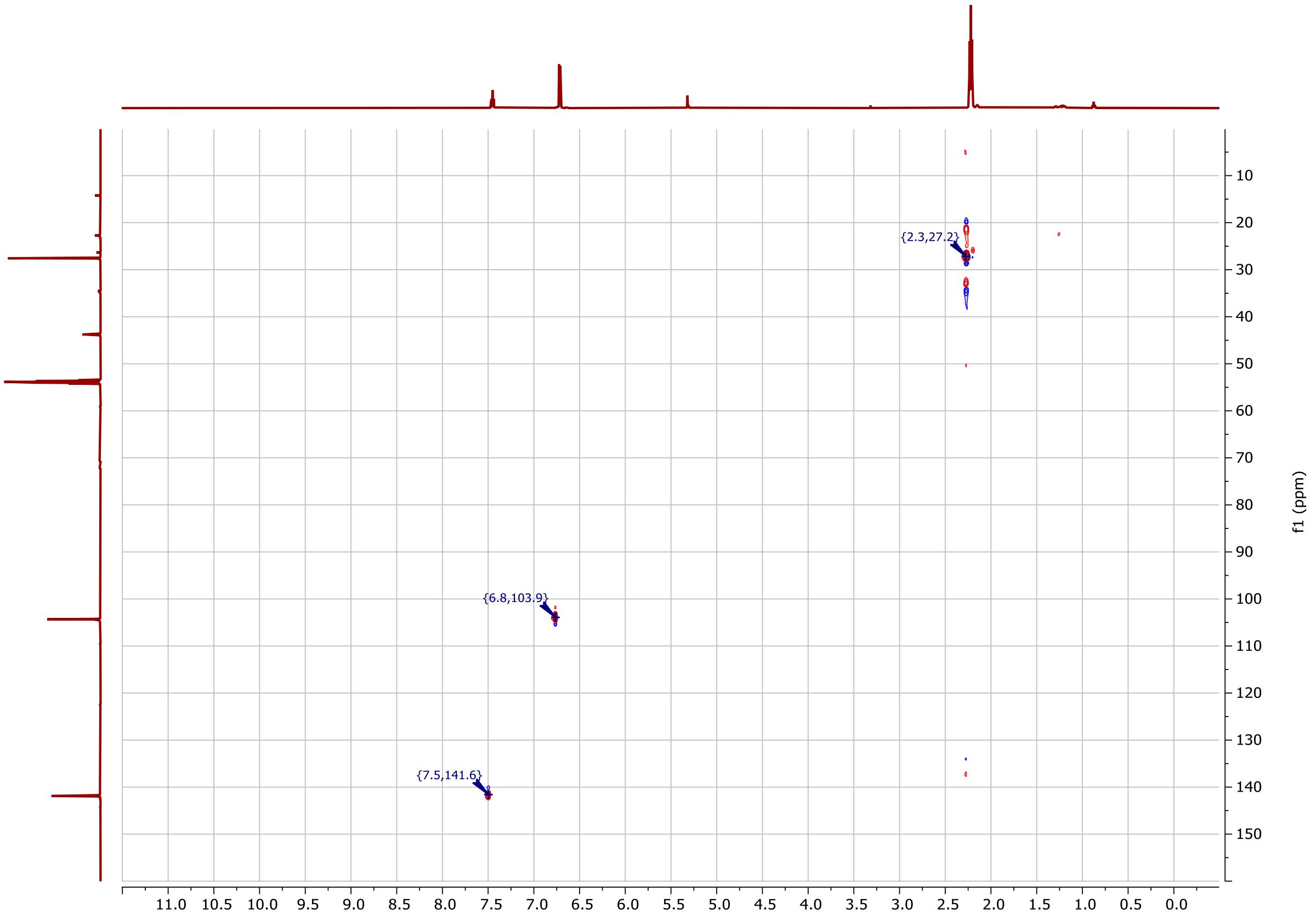

*Figure S 29. $^{1}H/^{13}C$-HSQC spectrum (23°C) of **1-I** in DCM-d$_2$.*

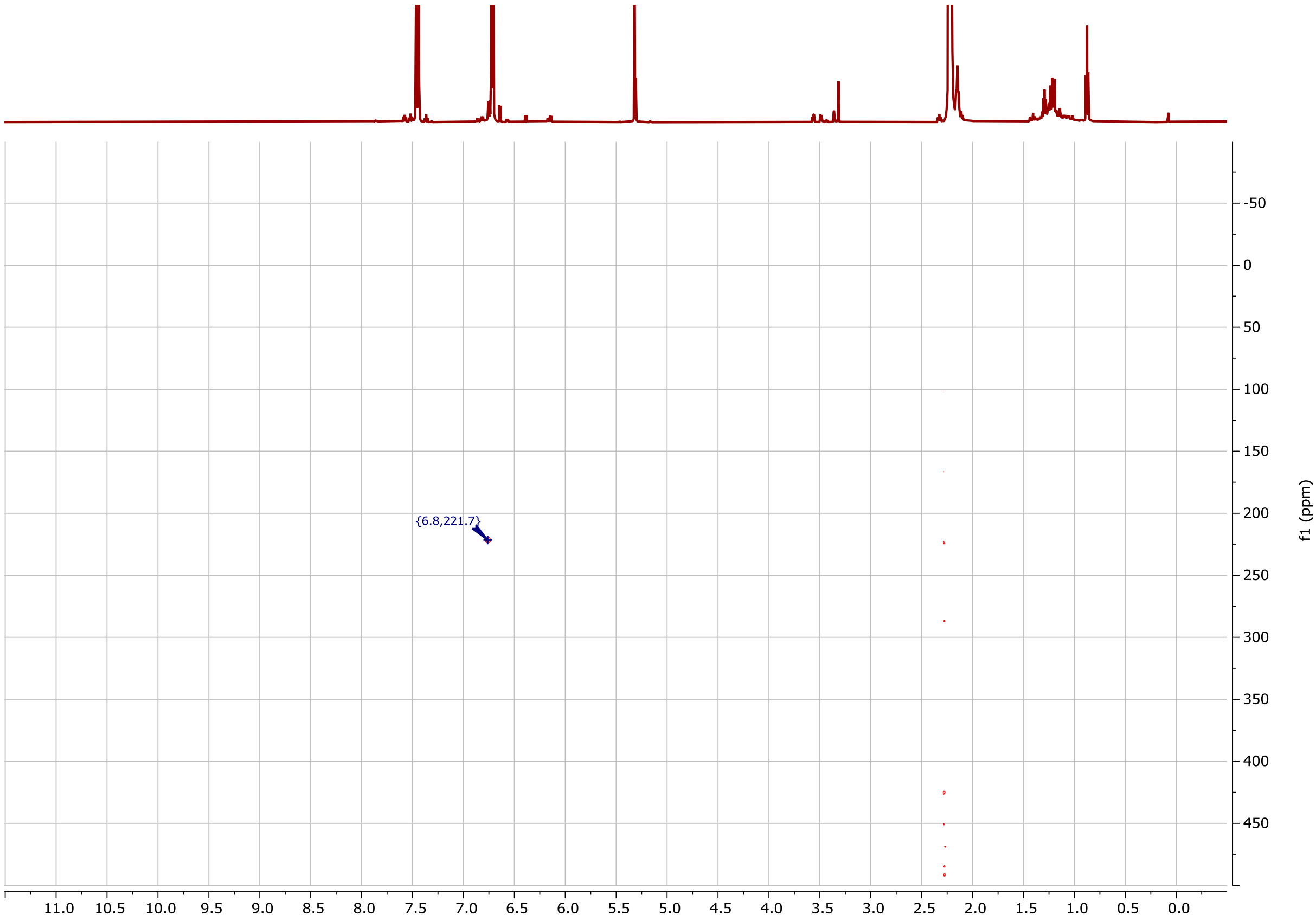

Figure S 30. $^{1}H/^{15}N$-HMBC spectrum (23°C) of ***1-I*** *in DCM-$_{d2}$.*

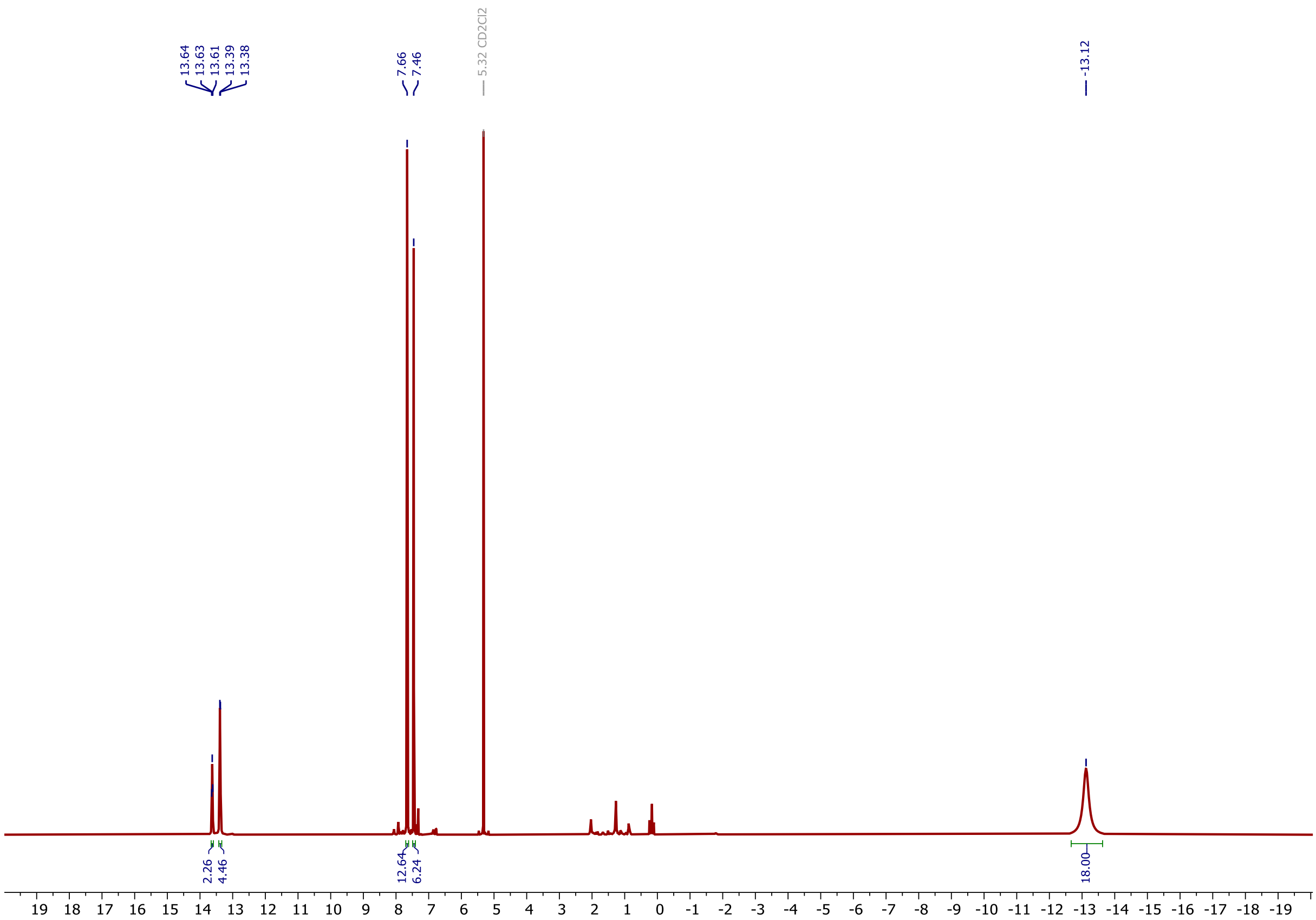

Figure S 31. ^{1}H-NMR spectrum (23°C, 600 MHz) of ***2-Cl*** *in DCM-d_2.*

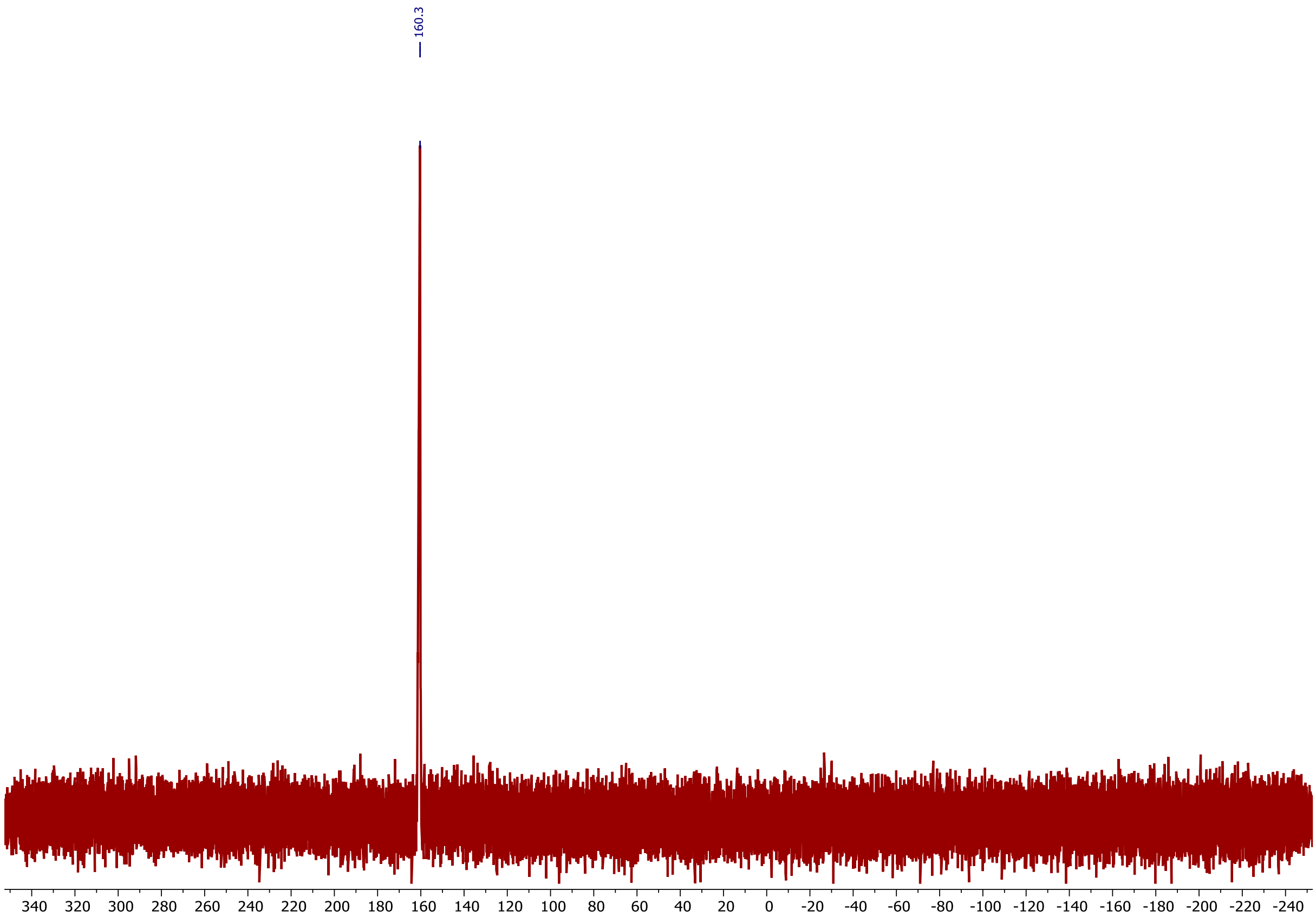

*Figure S 32. $^{31}P\{^{1}H\}$-NMR spectrum (23°C, 241 MHz) of **2Cl** in DCM-d$_2$.*

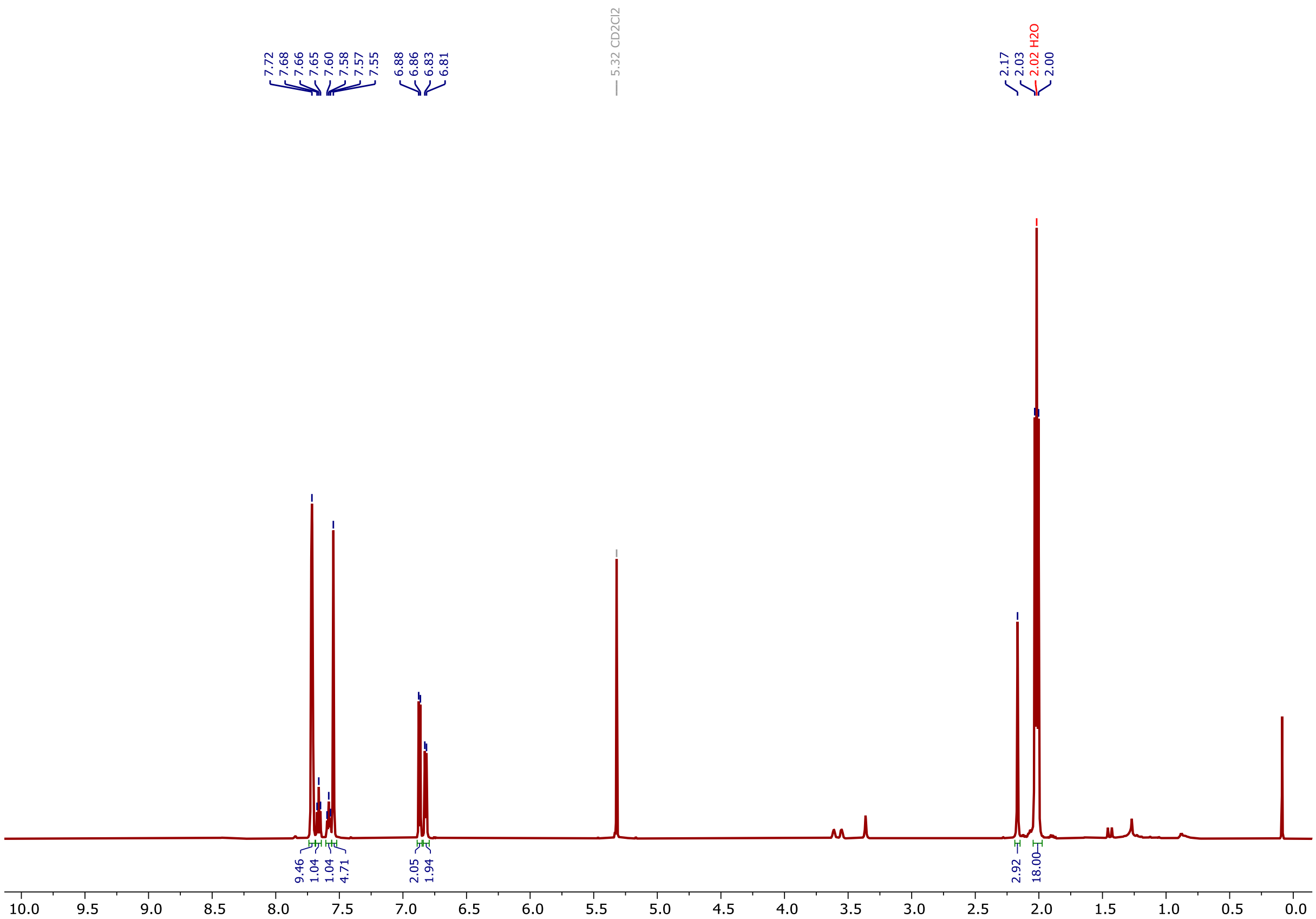

*Figure S 33. ^{1}H-NMR spectrum (23°C, 600 MHz) of **3-MeCN** in DCM-d$_2$.*

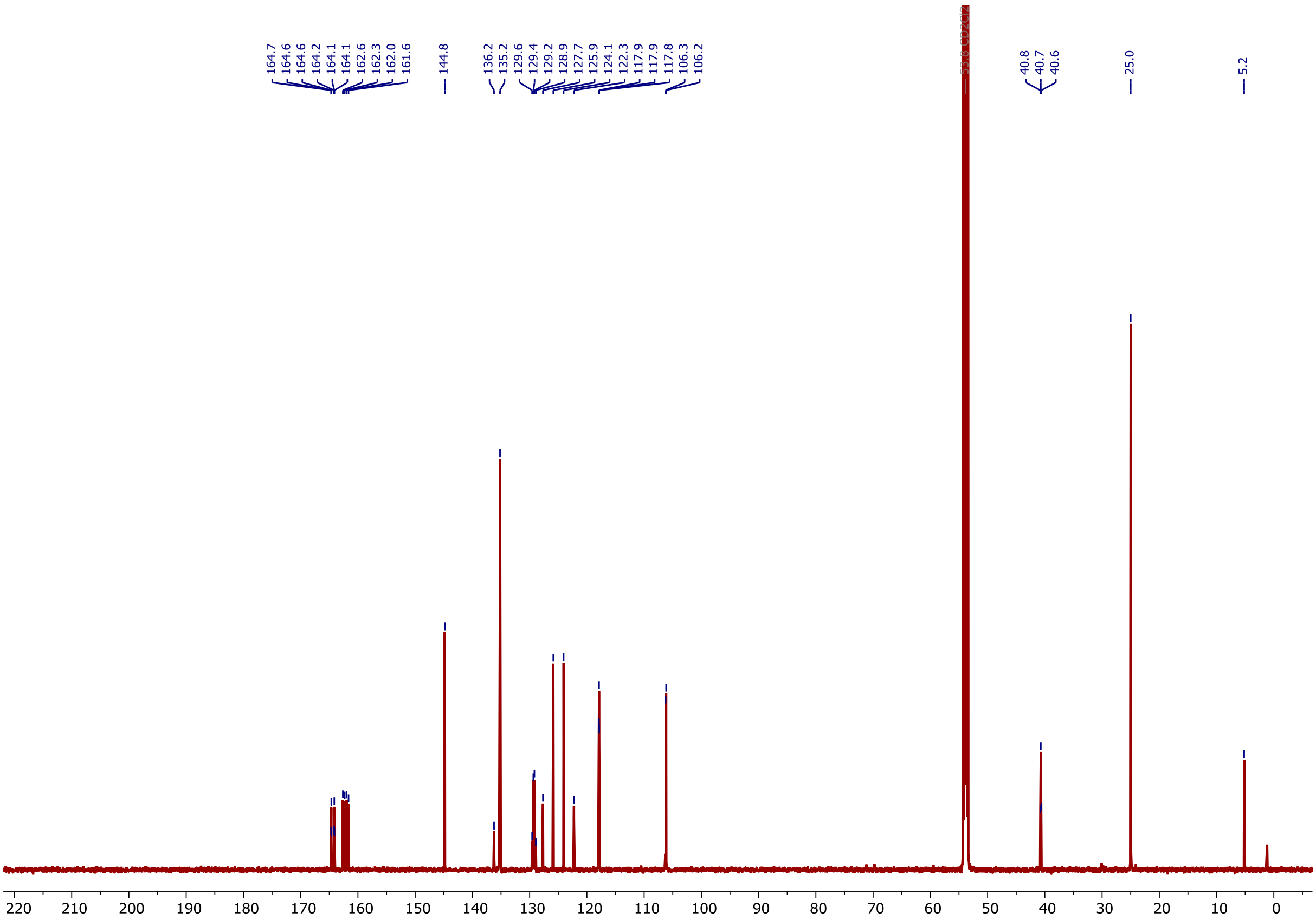

Figure S 34. ${}^{13}C\{{}^{1}H\}$-NMR spectrum (23°C, 150 MHz) of ***3-MeCN*** *in DCM-d_2.*

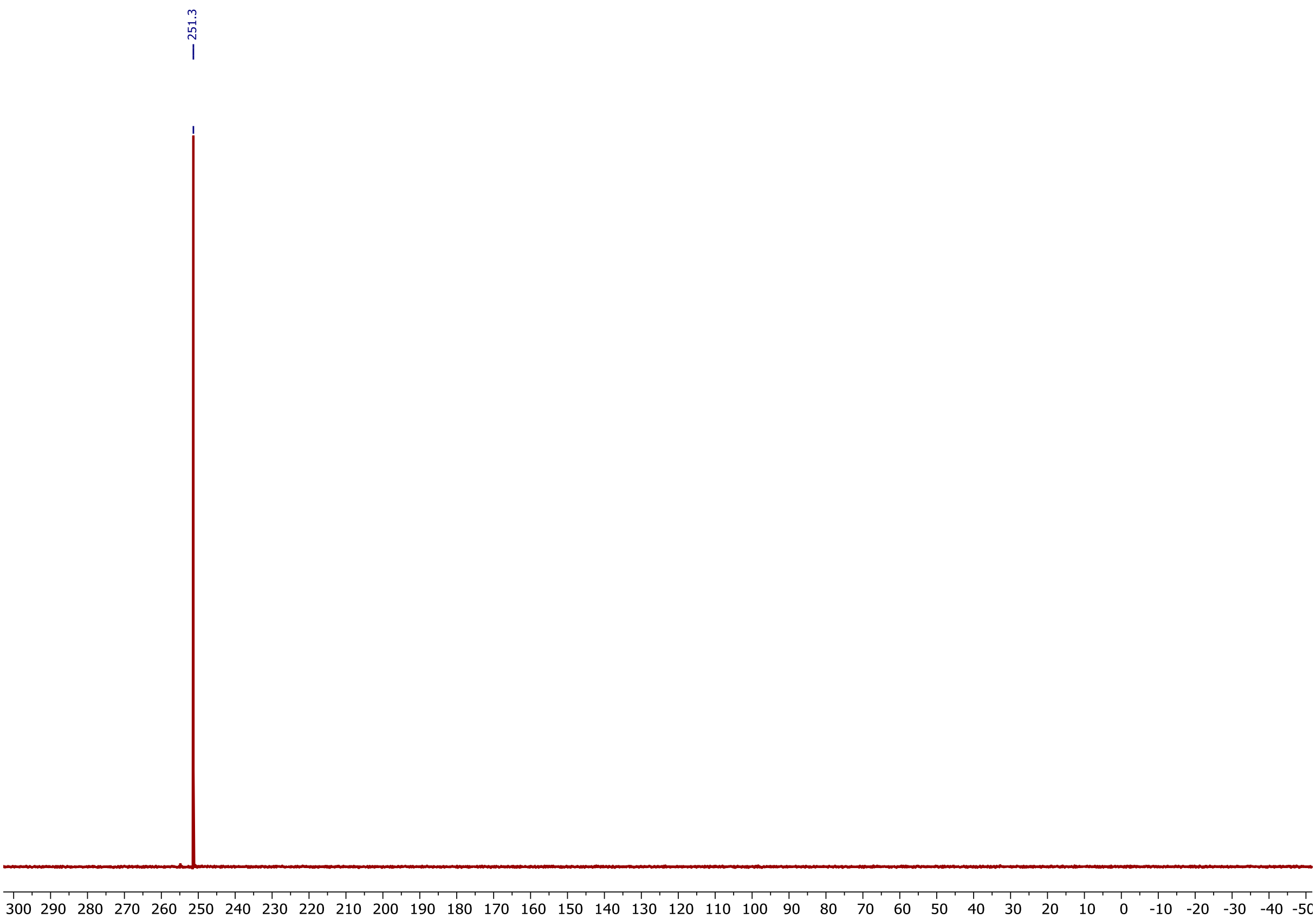

Figure S 35. ${}^{31}P\{{}^{1}H\}$-NMR spectrum (23°C, 241 MHz) of ***3-MeCN*** *in DCM-d_2.*

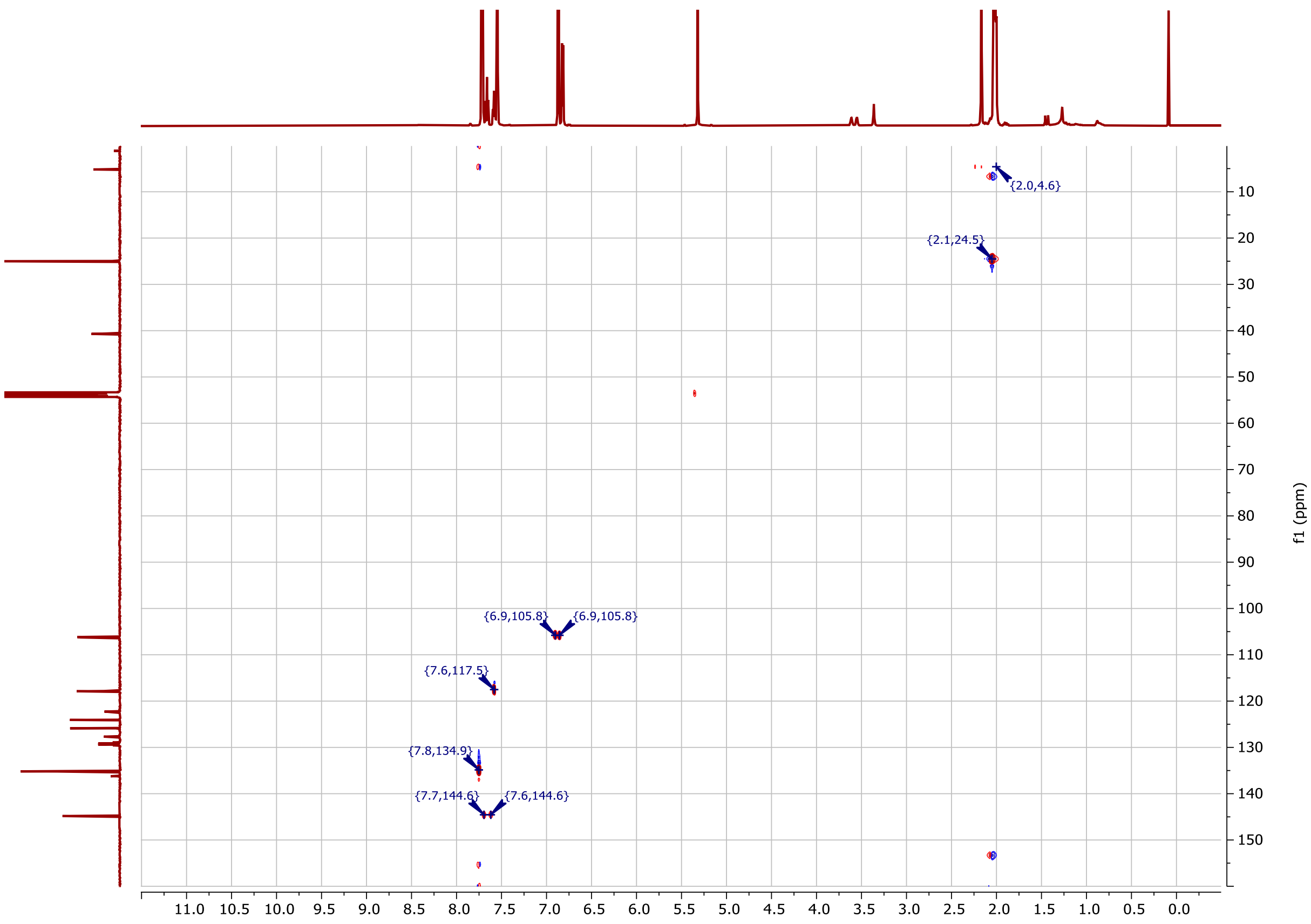

Figure S 36. $^{1}H/^{13}C$*-HSQC spectrum (23°C) of* ***3-MeCN*** *in DCM-*d_2*.*

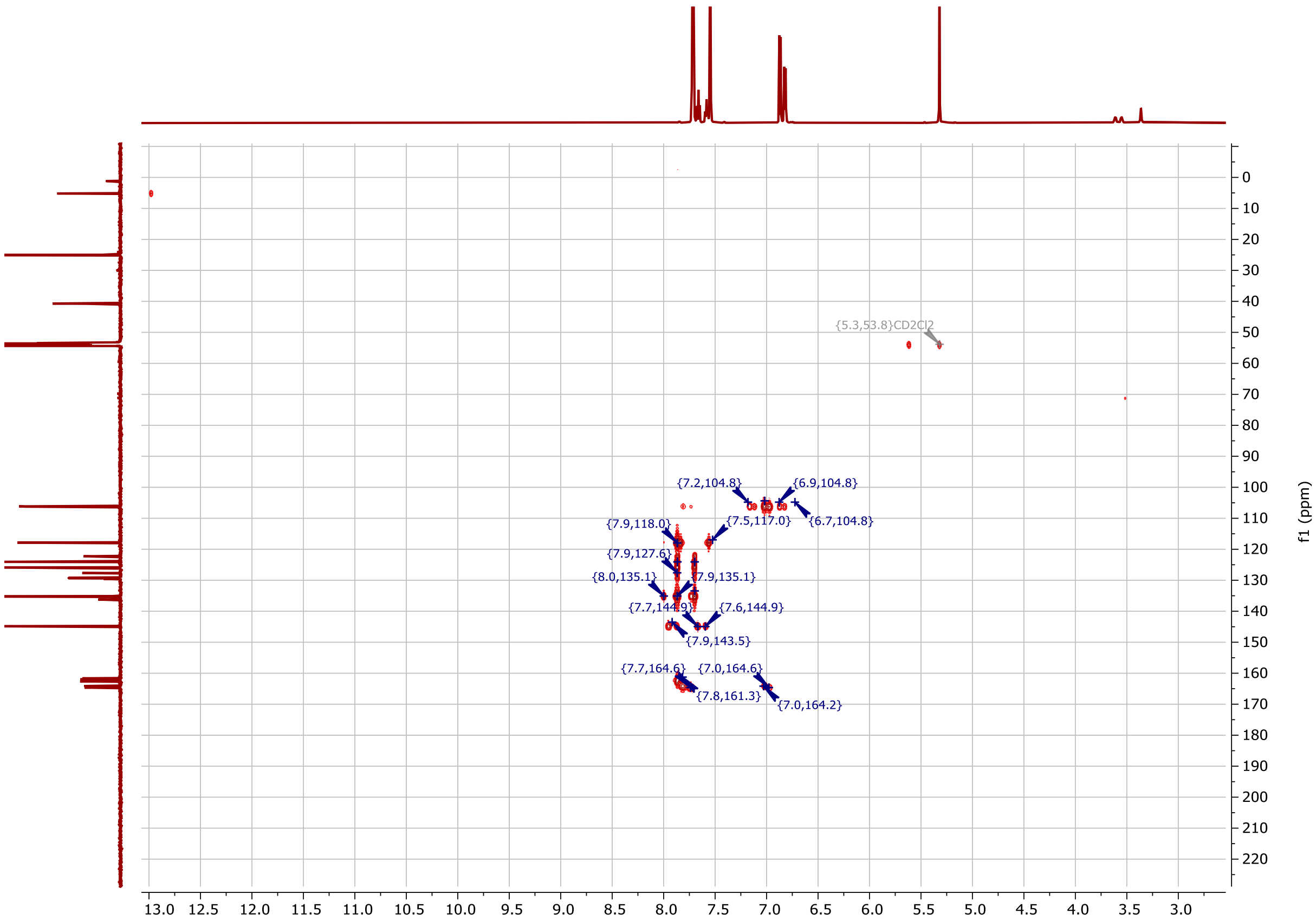

Figure S 37. $^{1}H/^{13}C$*-HMBC spectrum (23°C) of* ***3-MeCN*** *in DCM-*d_2*.*

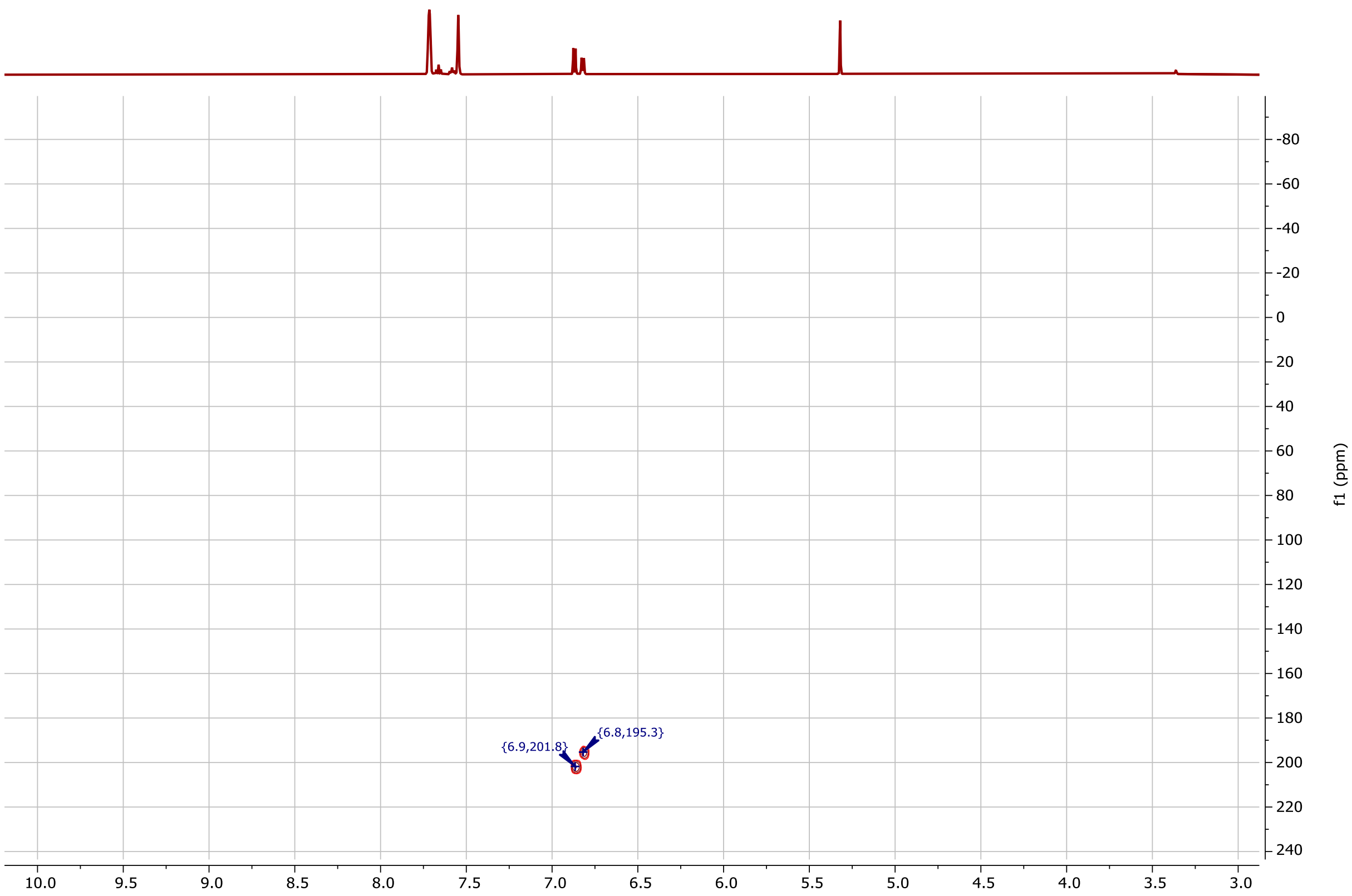

Figure S 38. $^{1}H/^{15}N$*-HMBC spectrum (23°C) of* ***3-MeCN*** *in DCM-*d_2*.*

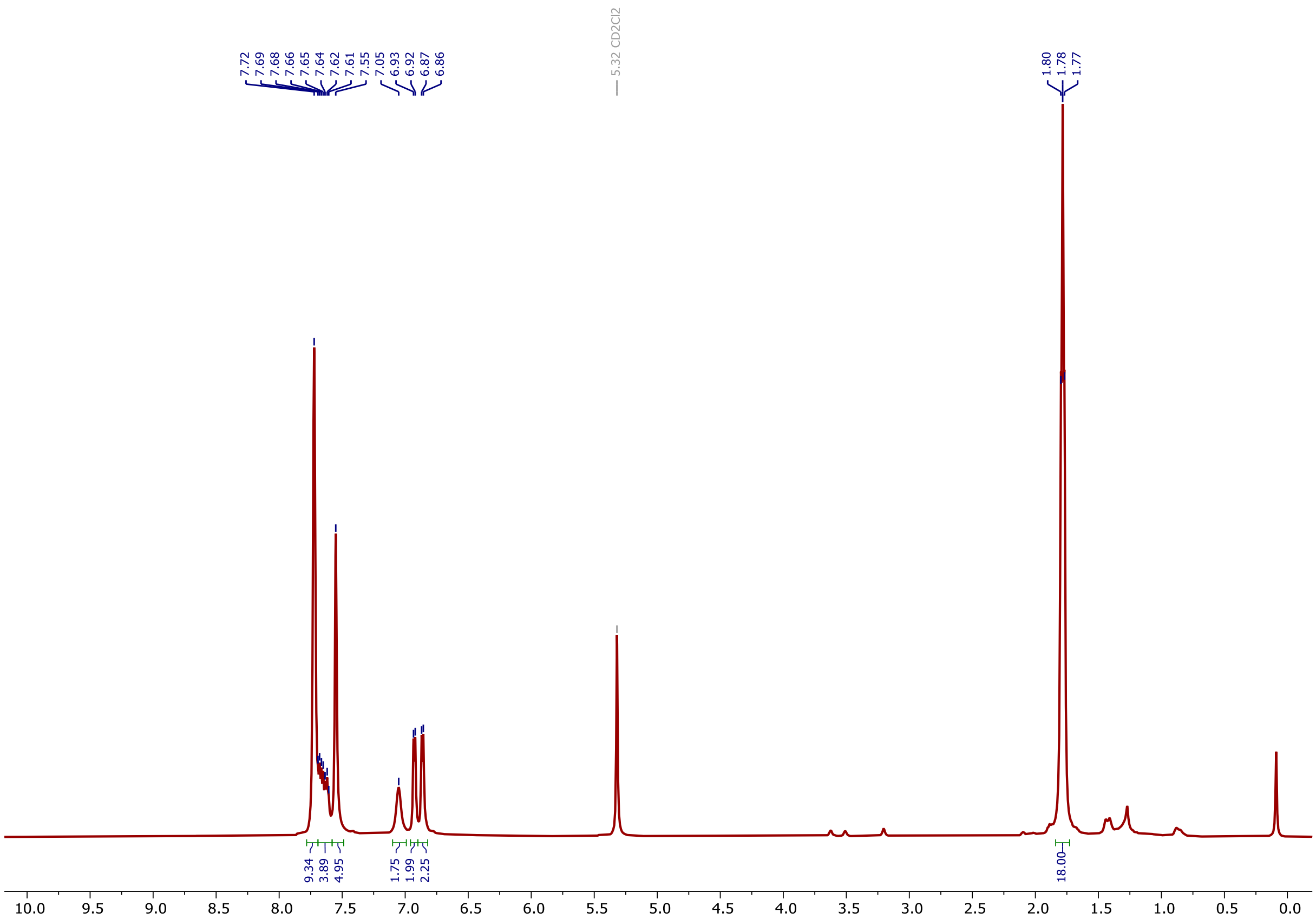

Figure S 39. ^{1}H*-NMR spectrum (23°C, 600 MHz) of* ***3-Pyr*** *in DCM-*d_2*.*

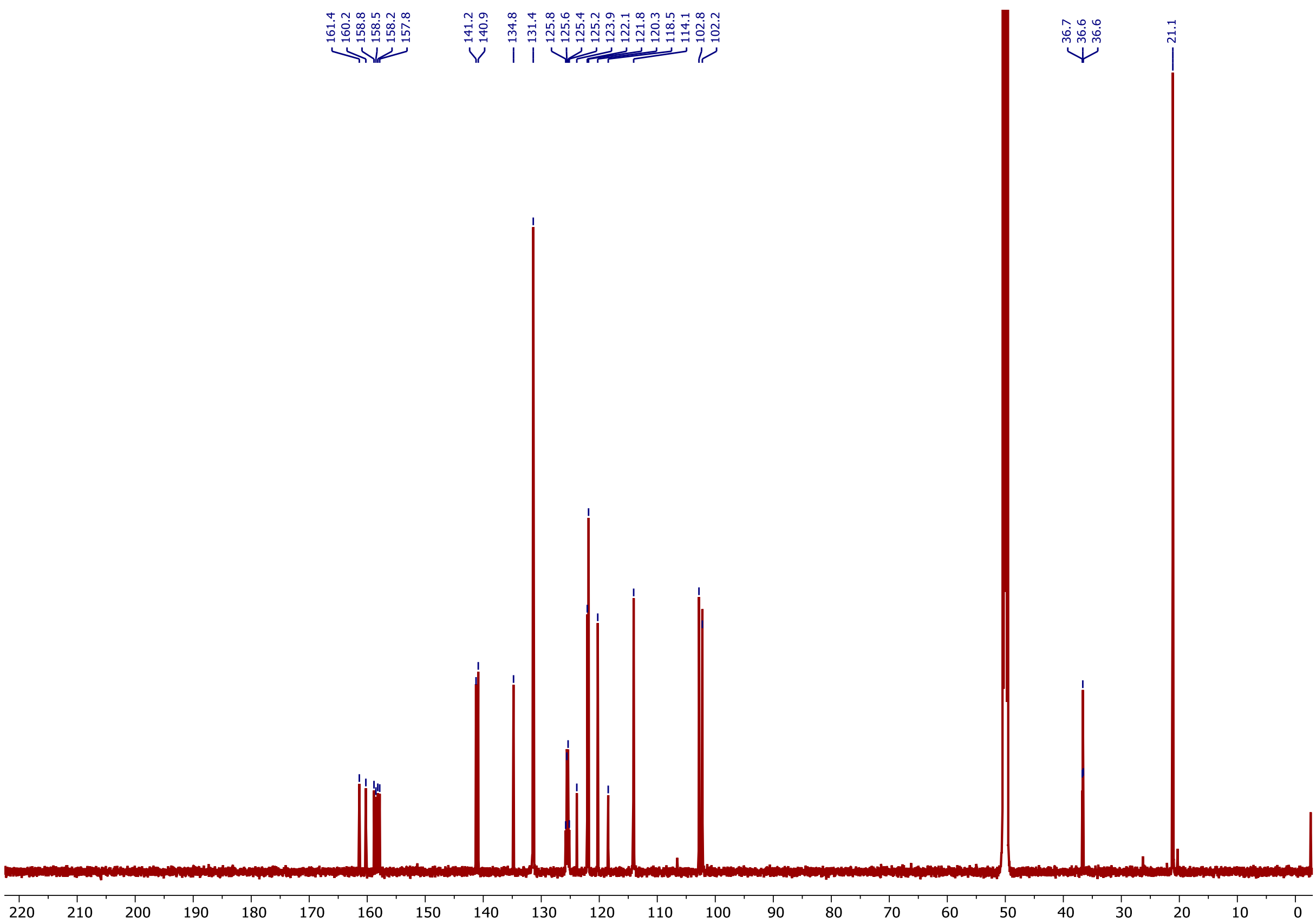

*Figure S 40. $^{13}C\{^{1}H\}$-NMR spectrum (23°C, 150 MHz) of **3-Pyr** in DCM-d_2.*

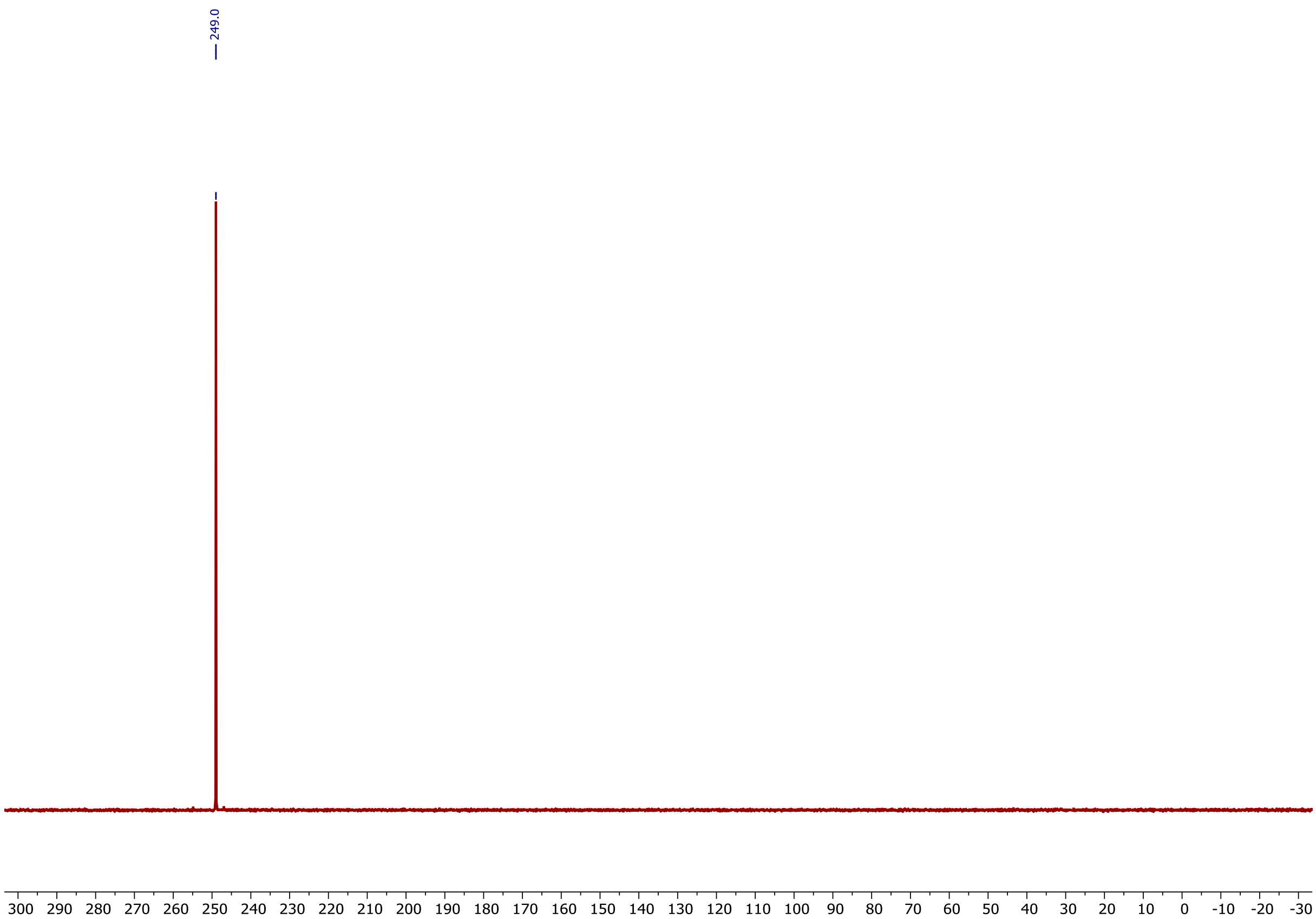

*Figure S 41. $^{31}P\{^{1}H\}$-NMR spectrum (23°C, 240 MHz) of **3-Pyr** in DCM-d_2.*

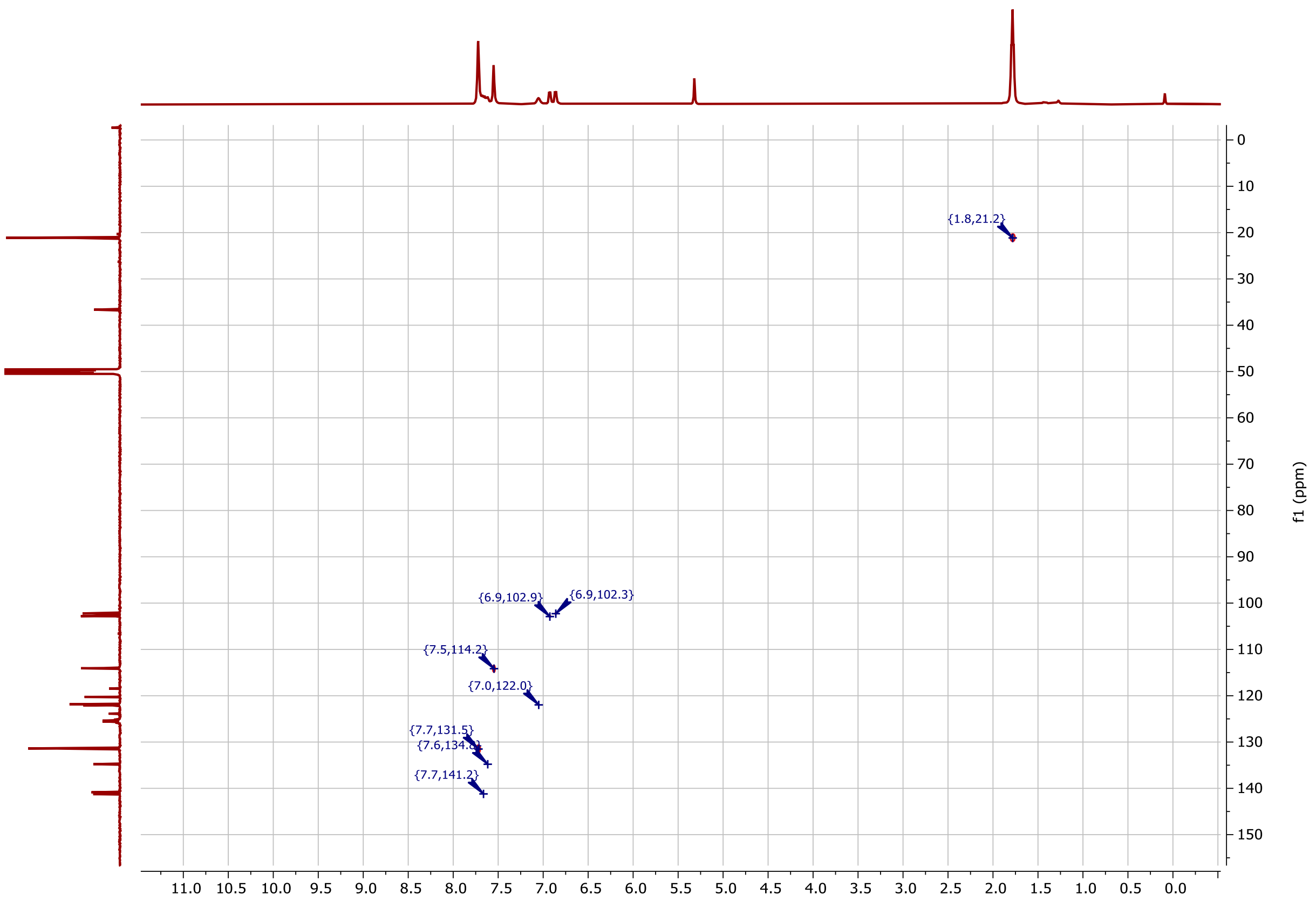

Figure S 42. $^{1}H/^{13}C$*-HSQC spectrum (23°C) of* ***3-Pyr*** *in DCM-*d_2*.*

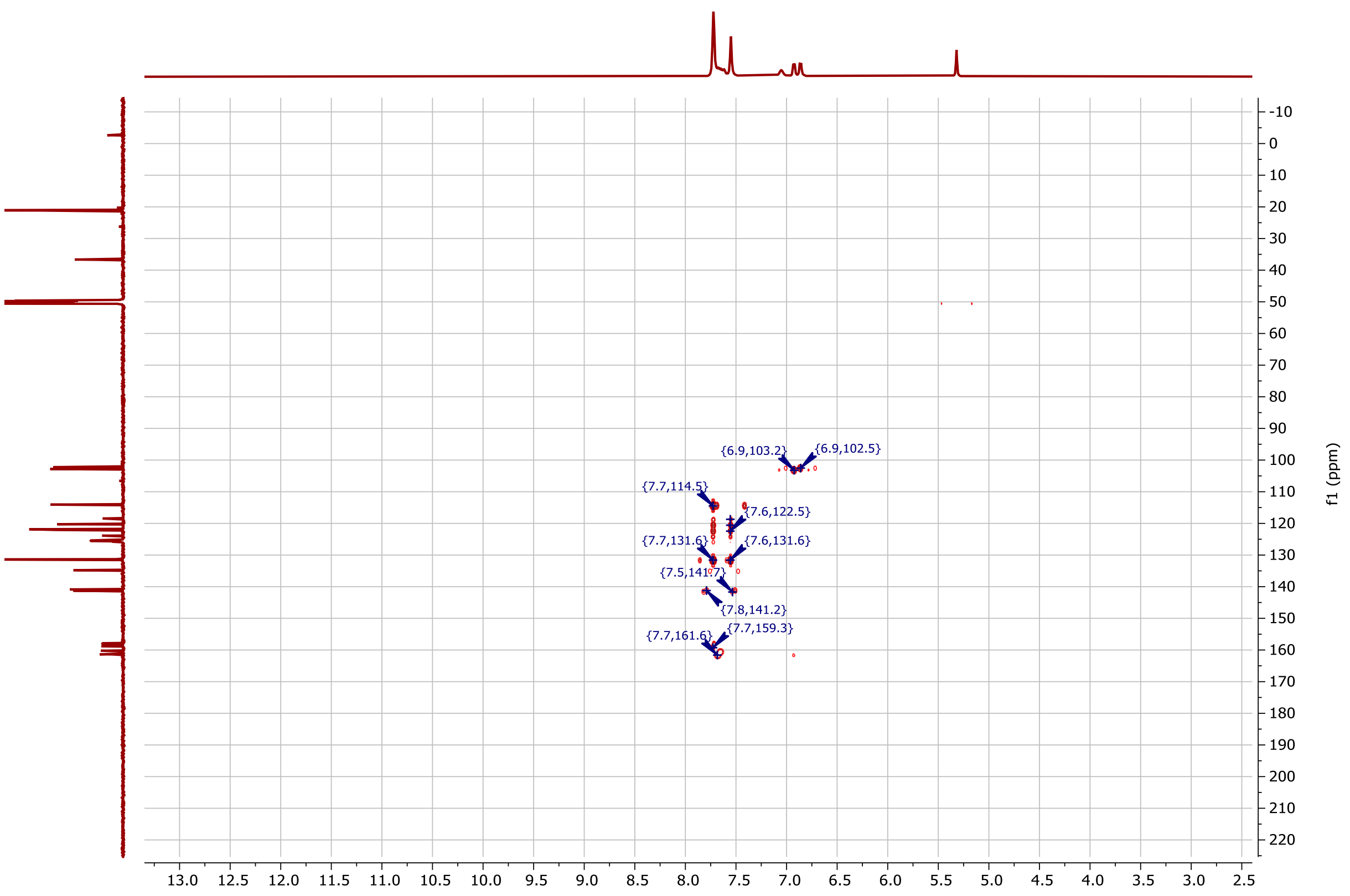

Figure S 43. $^{1}H/^{13}C$*-HMBC spectrum (23°C) of* ***3-Pyr*** *in DCM-*d_2*.*

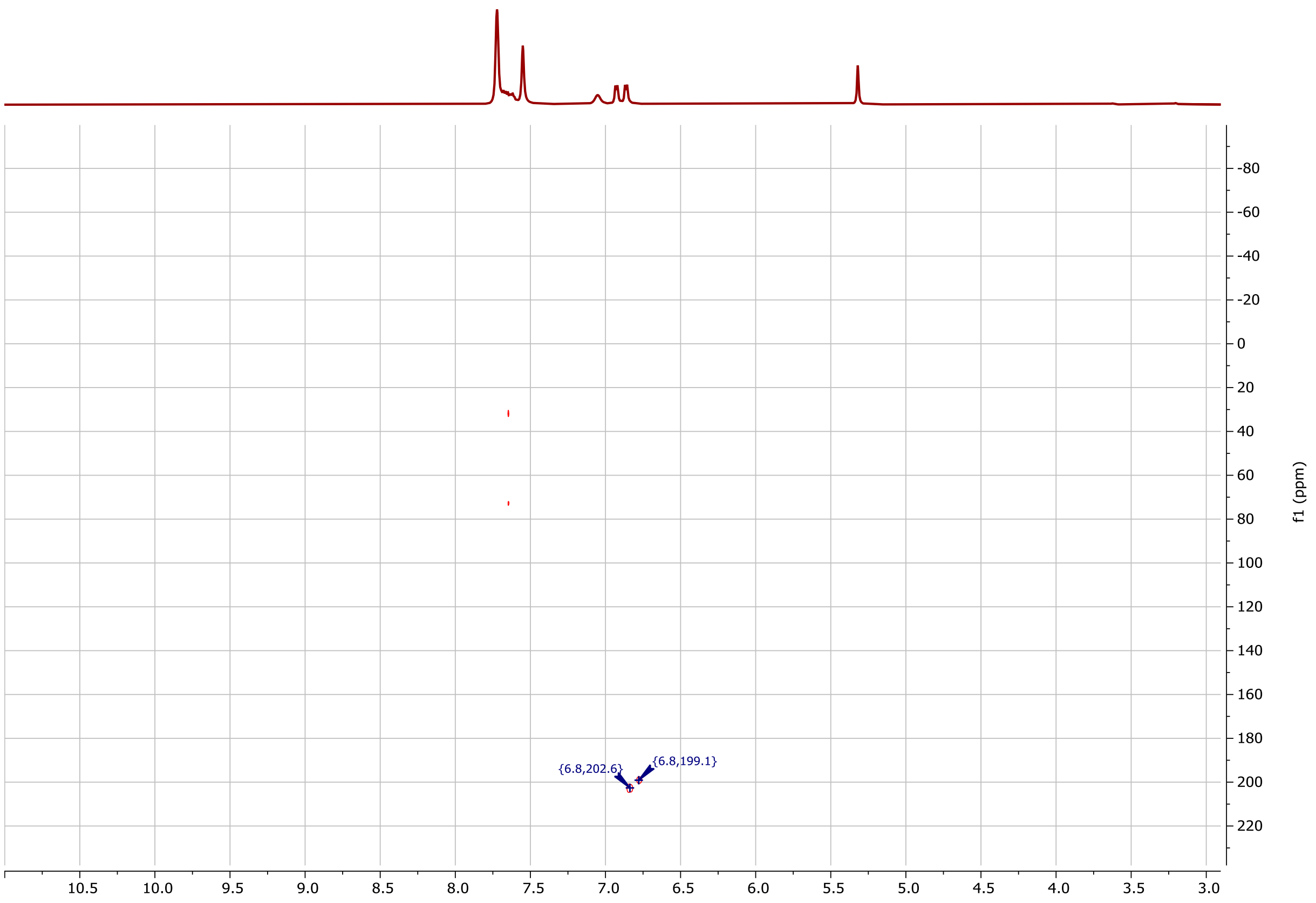

*Figure S 44. ^{1}H/^{15}N-HMBC spectrum (23°C) of **3-Pyr** in DCM-d$_2$.*

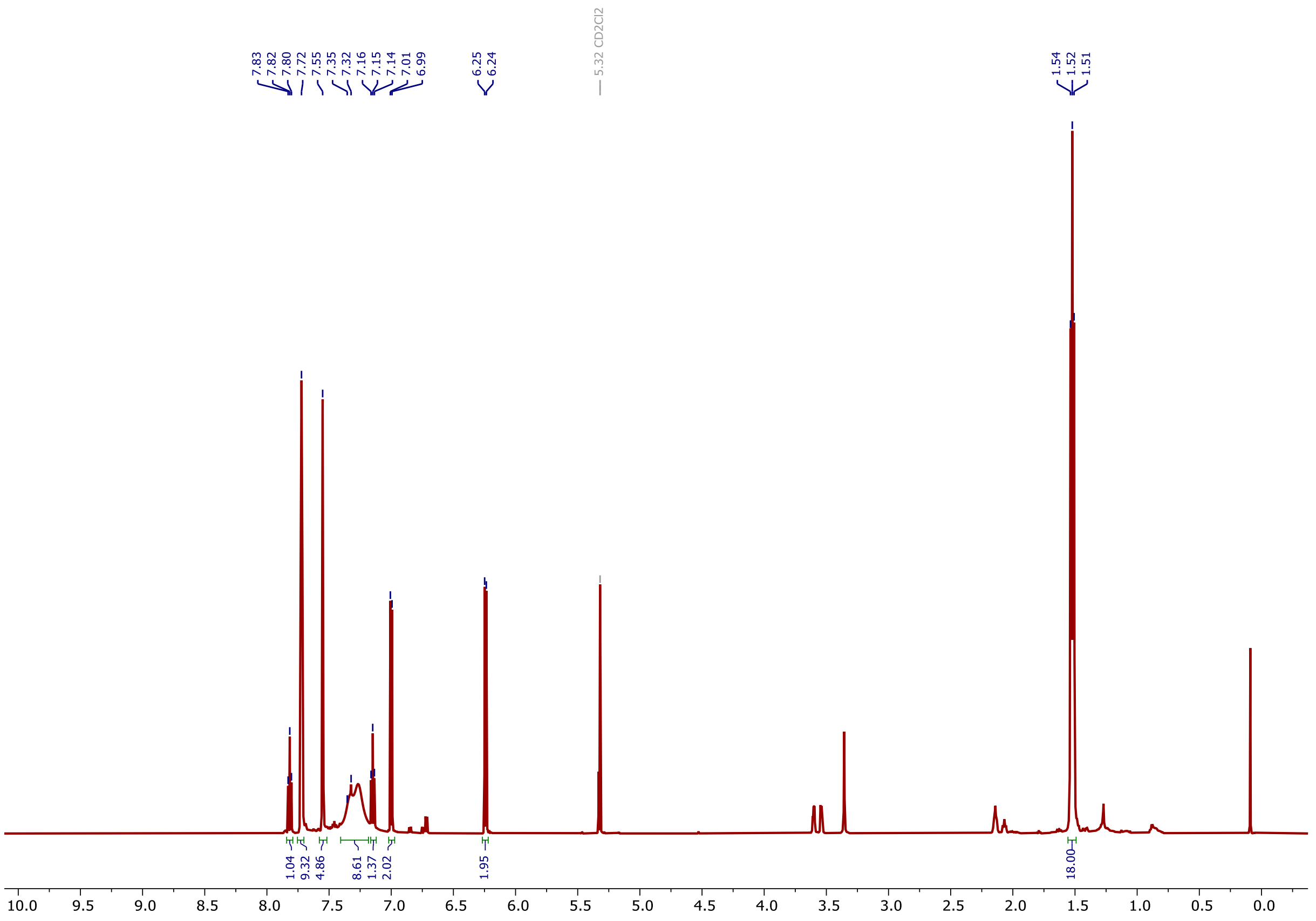

*Figure S 45. ^{1}H-NMR spectrum (23°C, 600 MHz) of **3-PPh$_3$** in DCM-d$_2$.*

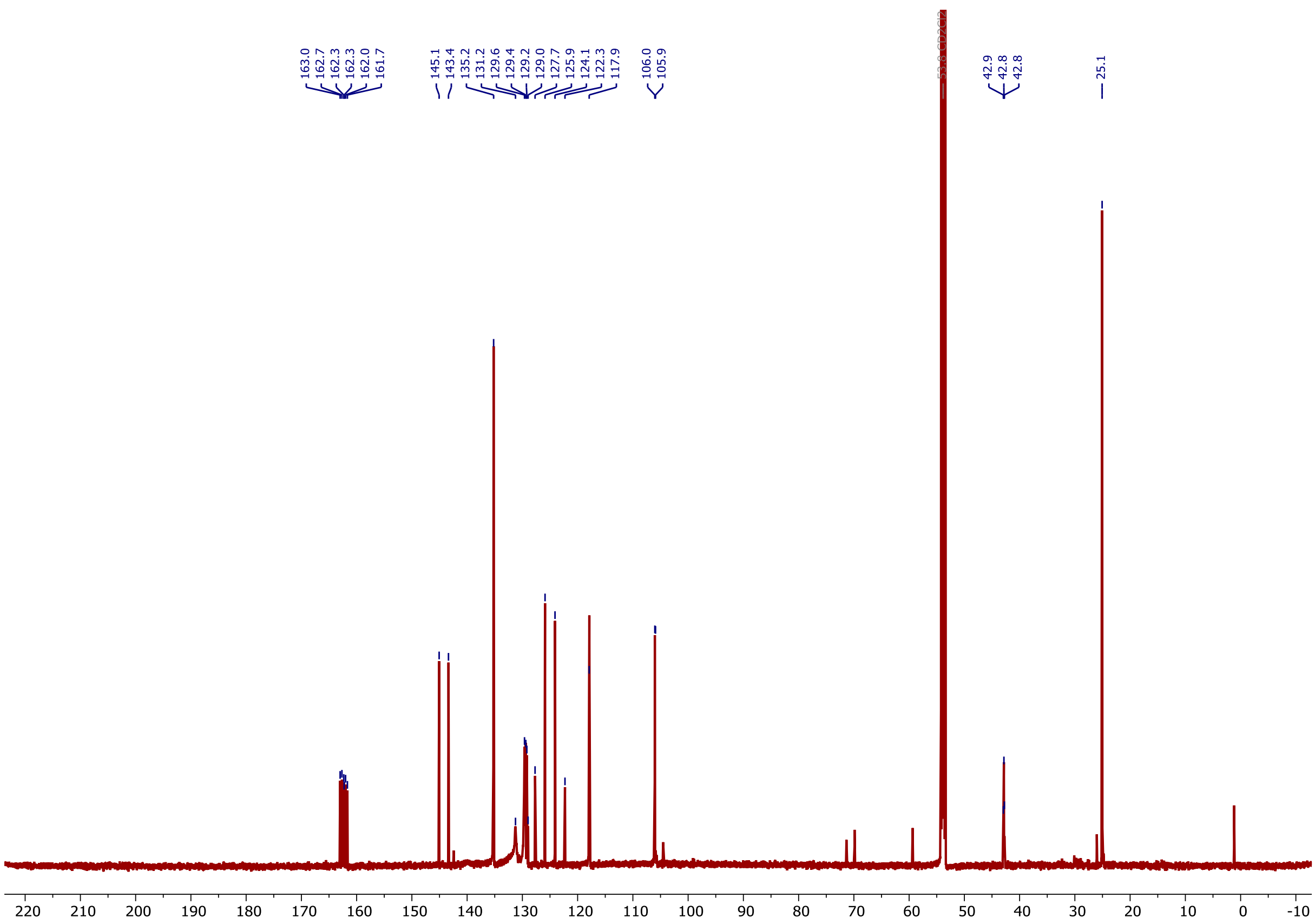

Figure S 46. $^{13}C\{^{1}H\}$-NMR spectrum (23°C, 150 MHz) of ***3-PPh₃*** *in DCM-d₂.*

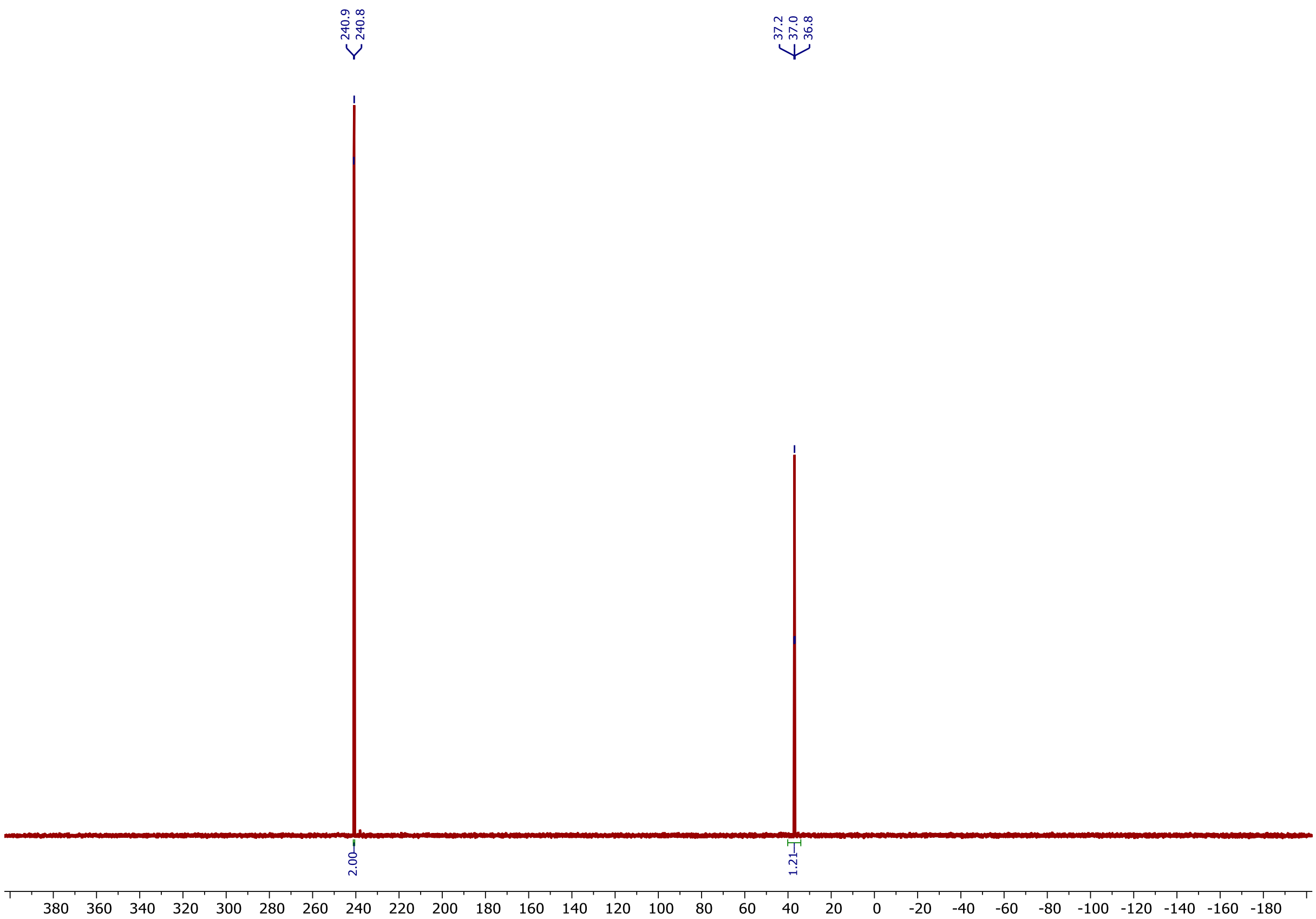

Figure S 47. $^{31}P\{^{1}H\}$-NMR spectrum (23°C, 240 MHz) of ***3-PPh₃*** *in DCM-d₂.*

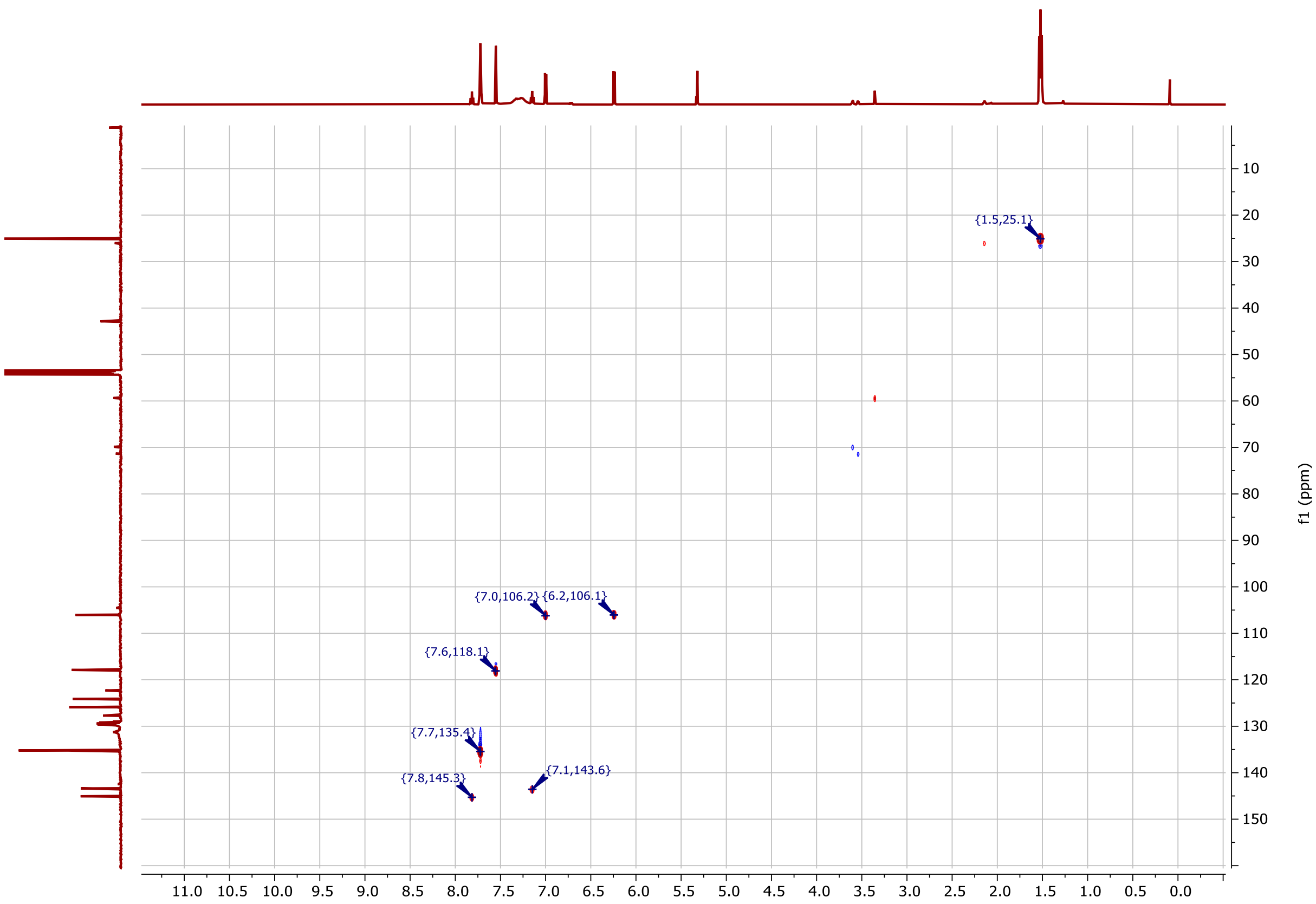

Figure S 48. $^{1}H/^{13}C$*-HSQC spectrum (23°C) of* ***3-PPh₃*** *in DCM-d₂.*

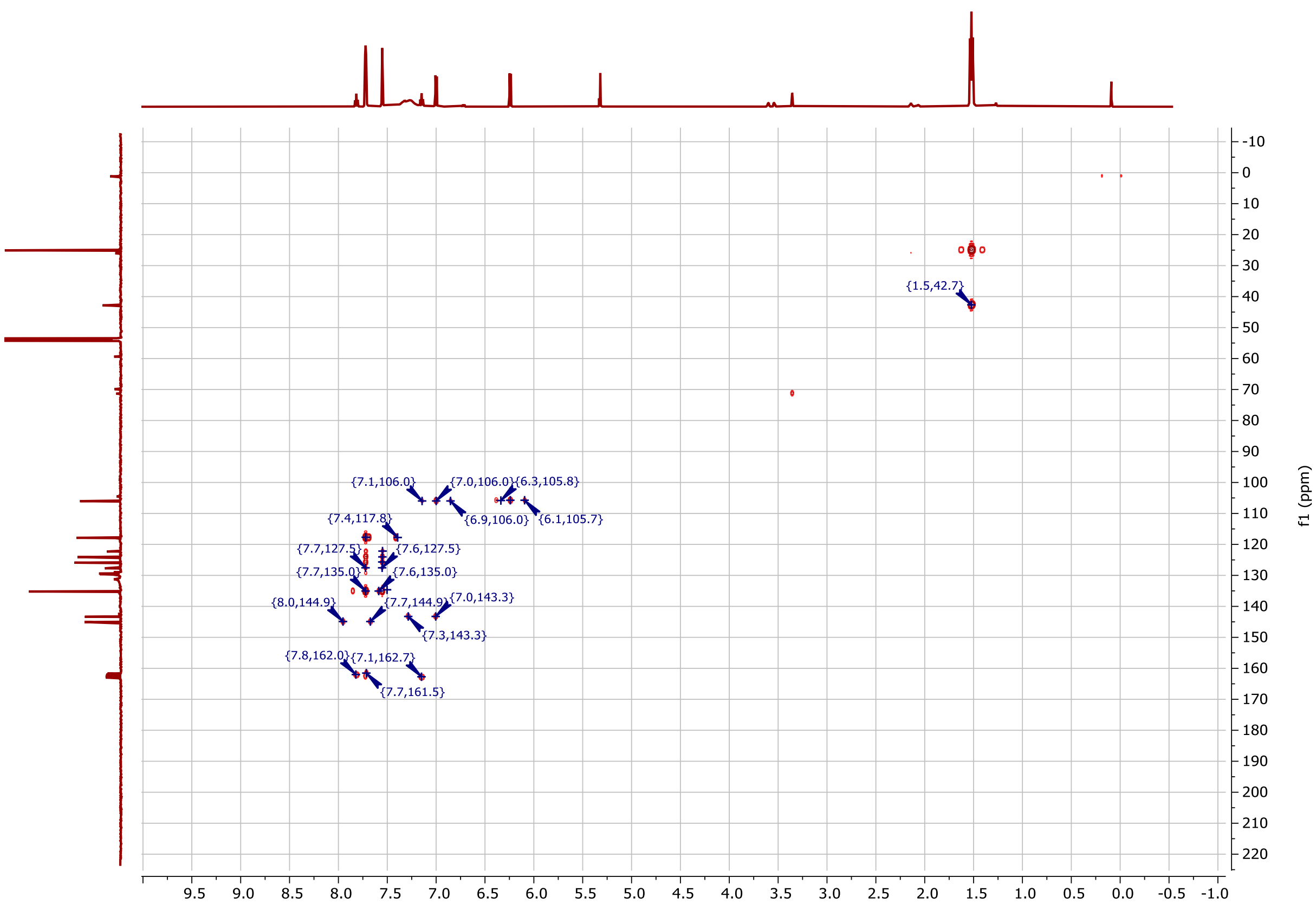

Figure S 49. $^{1}H/^{13}C$*-HMBC spectrum (23°C) of* ***3-PPh₃*** *in DCM-d₂.*

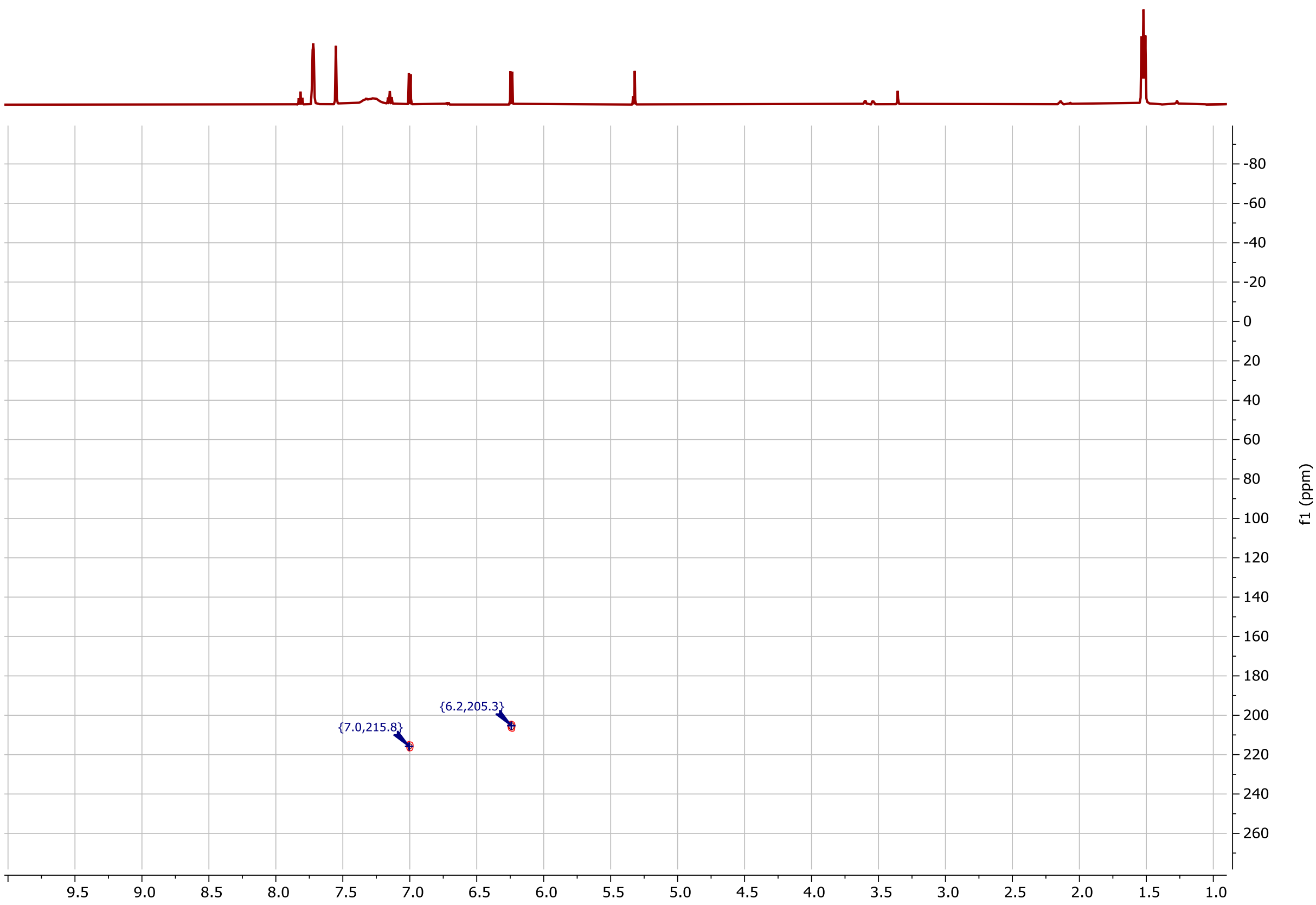

Figure S 50. ^{1}H/^{15}N-HMBC spectrum (23°C) of ***3-PPh₃*** *in DCM-d₂.*

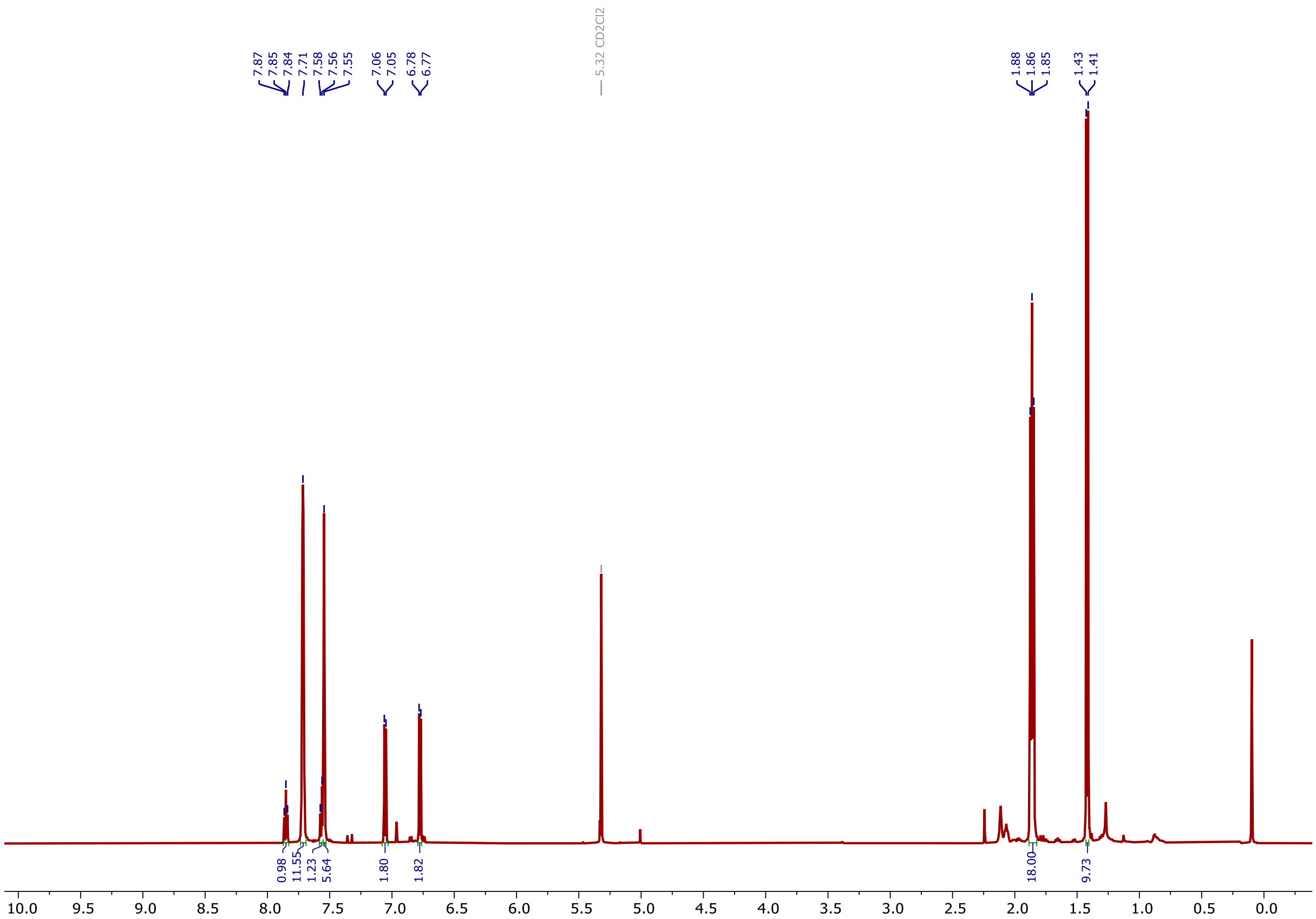

Figure S 51. ^{1}H-NMR spectrum (23°C, 600 MHz) of ***3-PMe₃*** *in DCM-d₂.*

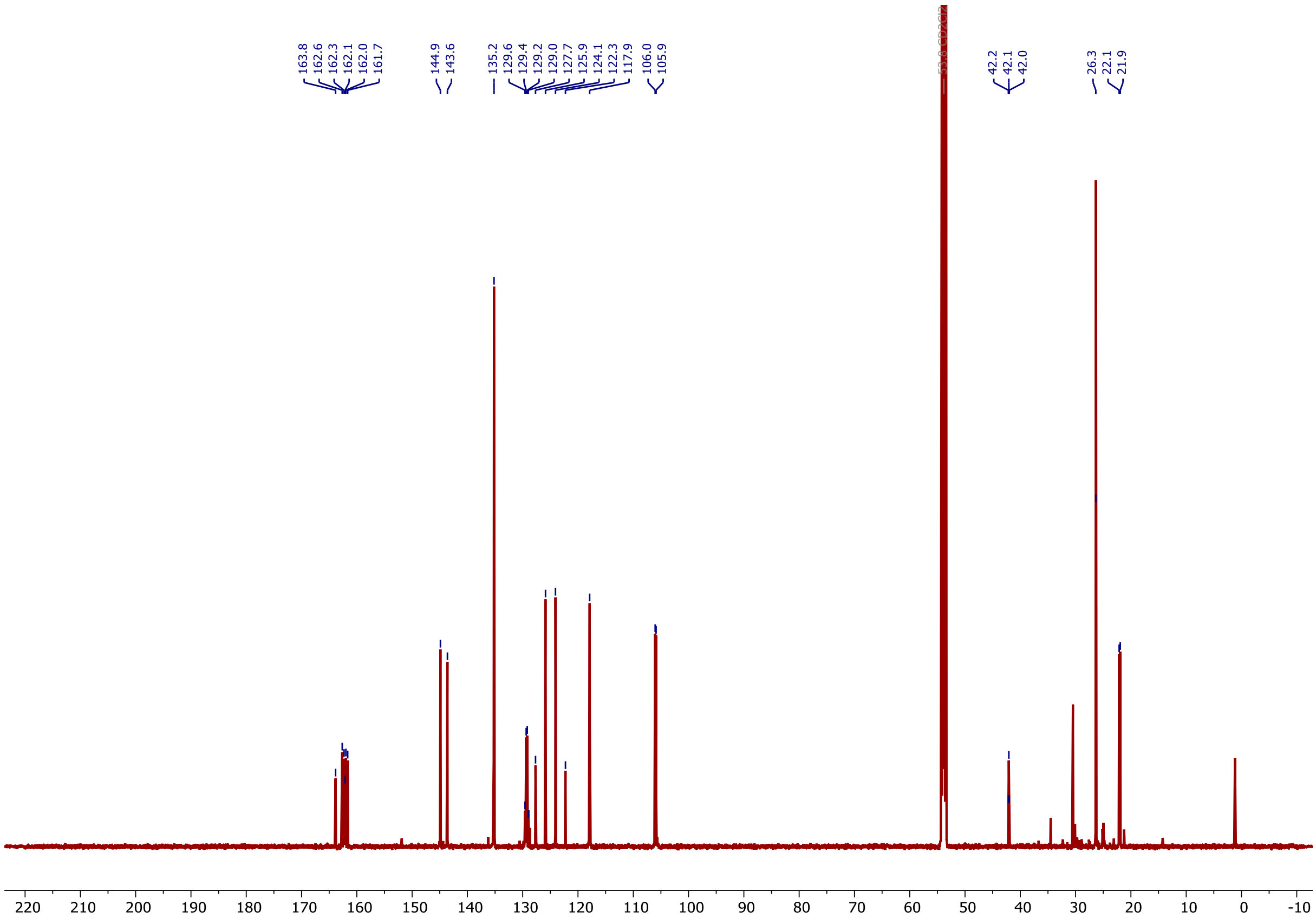

*Figure S 52. $^{13}C\{^{1}H\}$-NMR spectrum (23°C, 150 MHz) of **3-PMe₃** in DCM-d₂.*

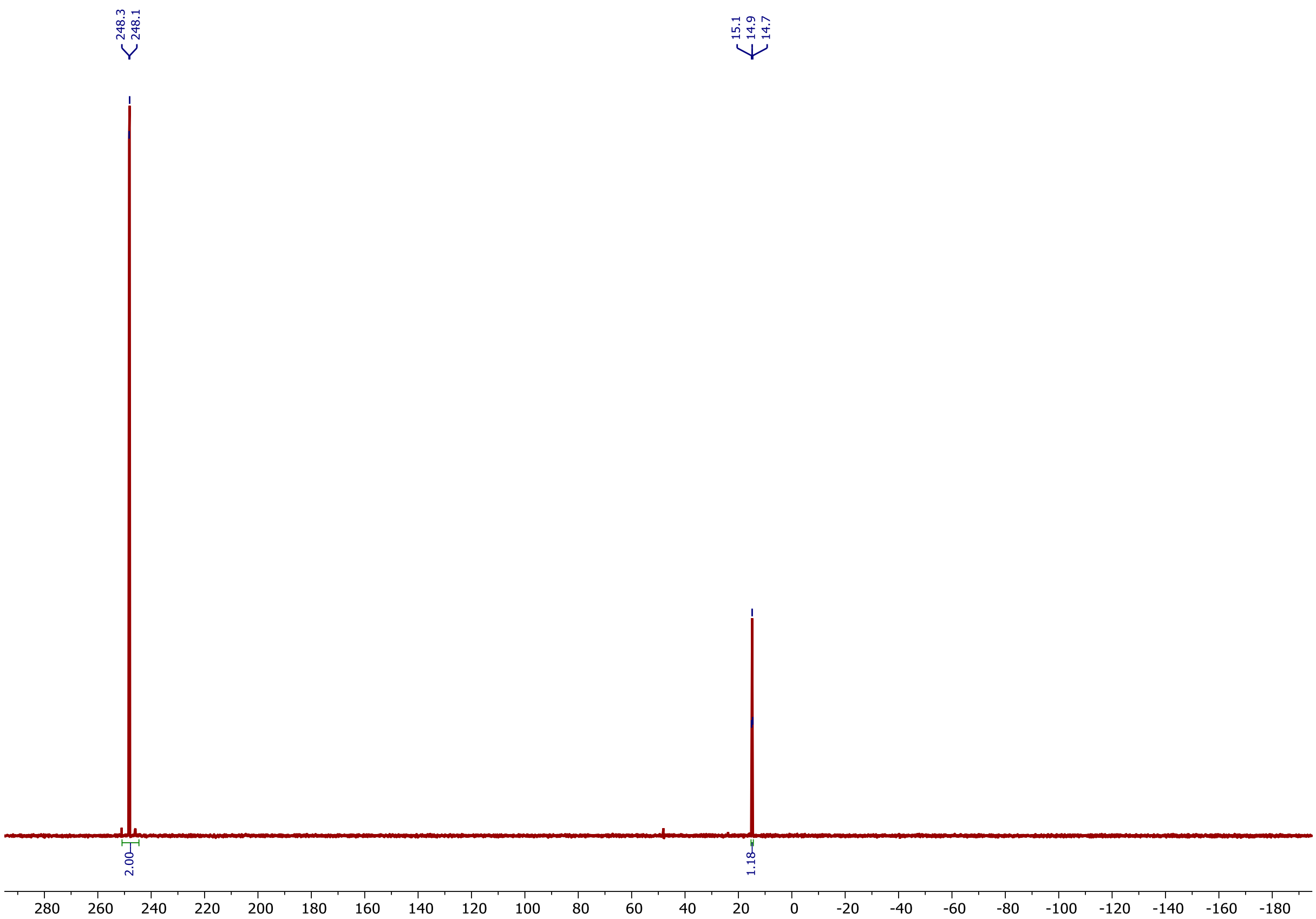

*Figure S 53. $^{31}P\{^{1}H\}$-NMR spectrum (23°C, 240 MHz) of **3-PMe_3** in DCM-d_2.*

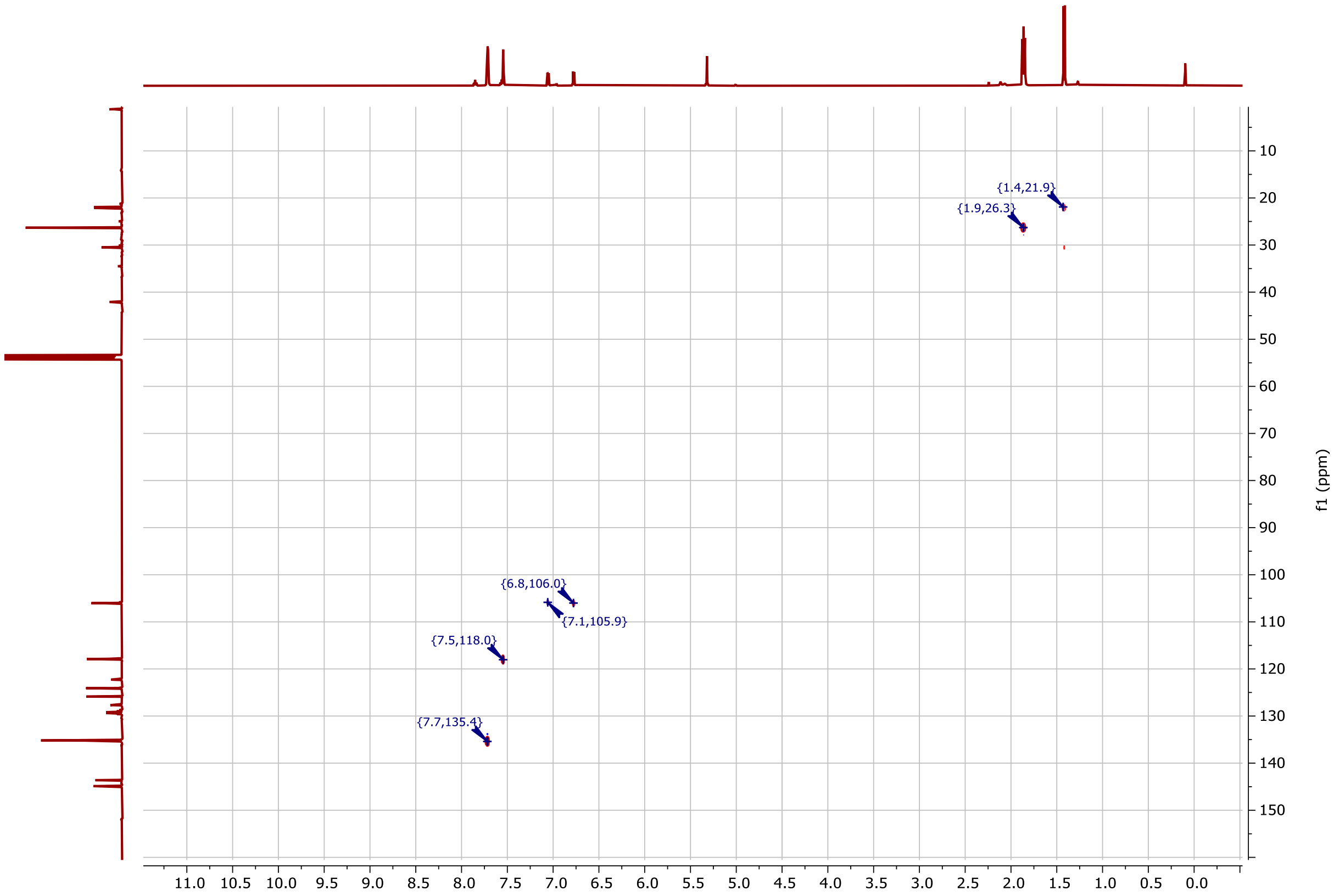

Figure S 54. *¹H/¹³C-HSQC spectrum (23°C) of* ***3-PMe₃*** *in DCM-d₂.*

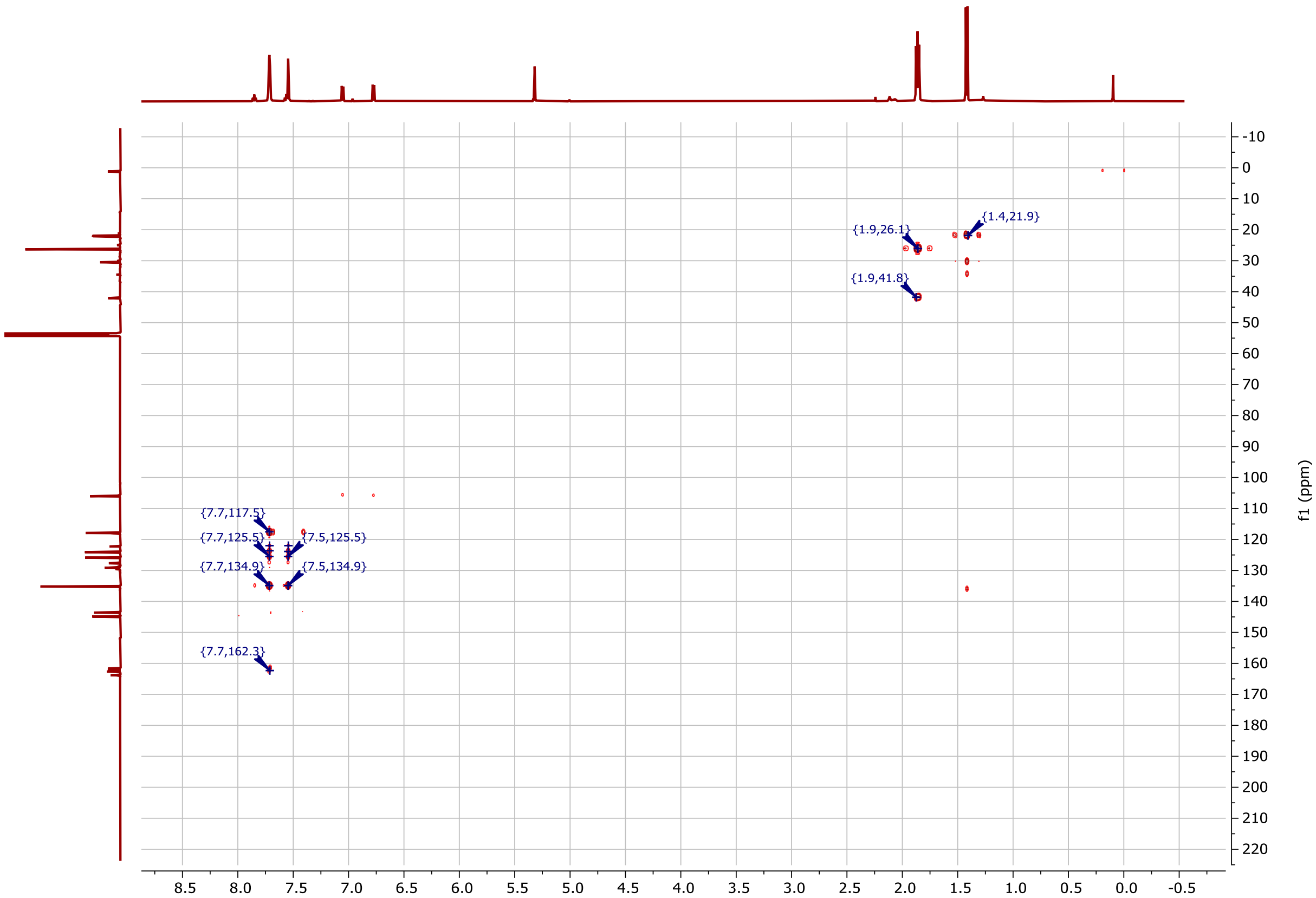

Figure S 55. *¹H/¹³C-HMBC spectrum (23°C) of* ***3-PMe₃*** *in DCM-d₂.*

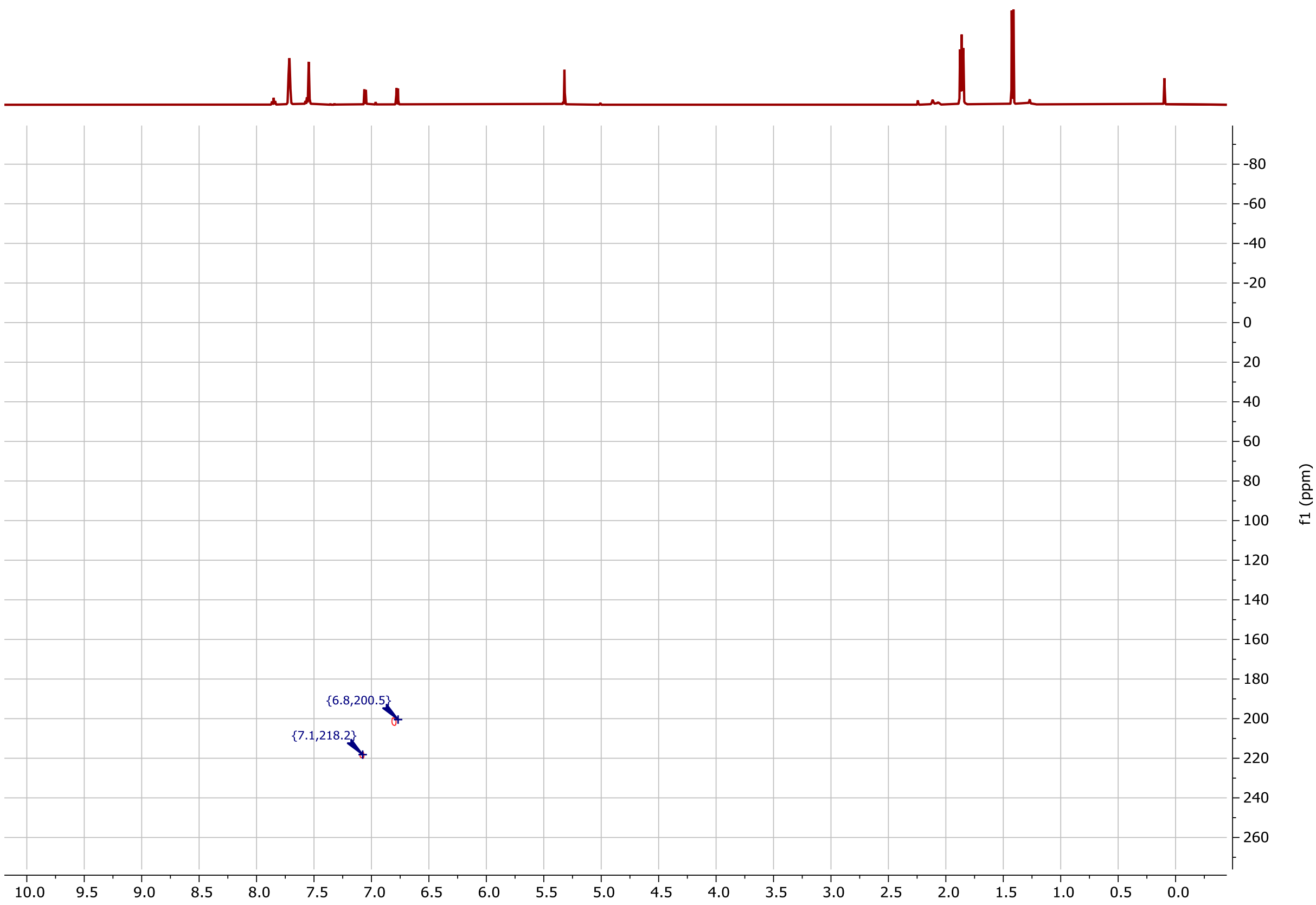

Figure S 56. $^{1}H/^{15}N$*-HMBC spectrum (23°C) of* ***3-PMe₃*** *in DCM-d₂.*

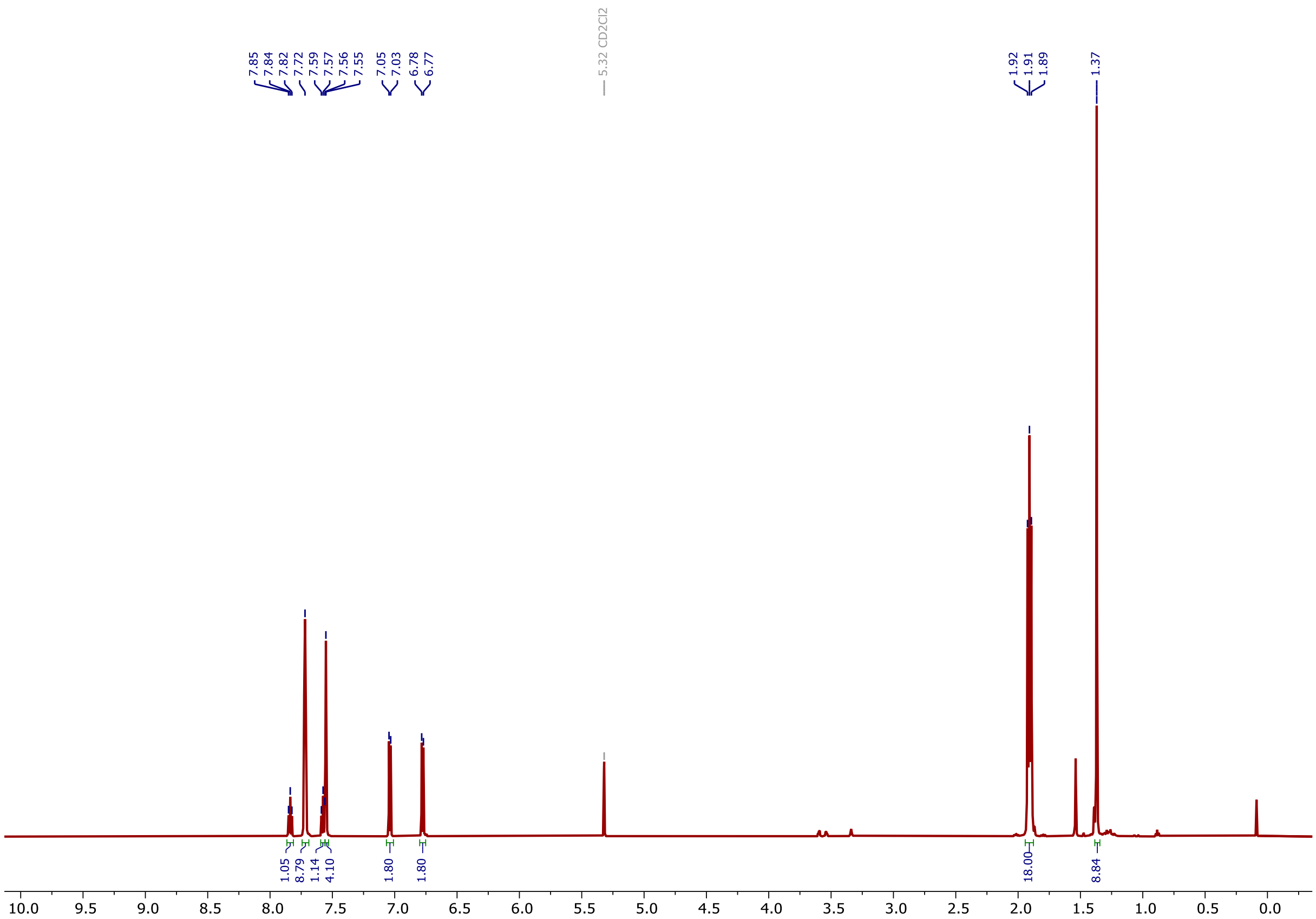

Figure S 57. ^{1}H*-NMR spectrum (23°C, 600 MHz) of* ***3-tBuNC*** *in DCM-d₂.*

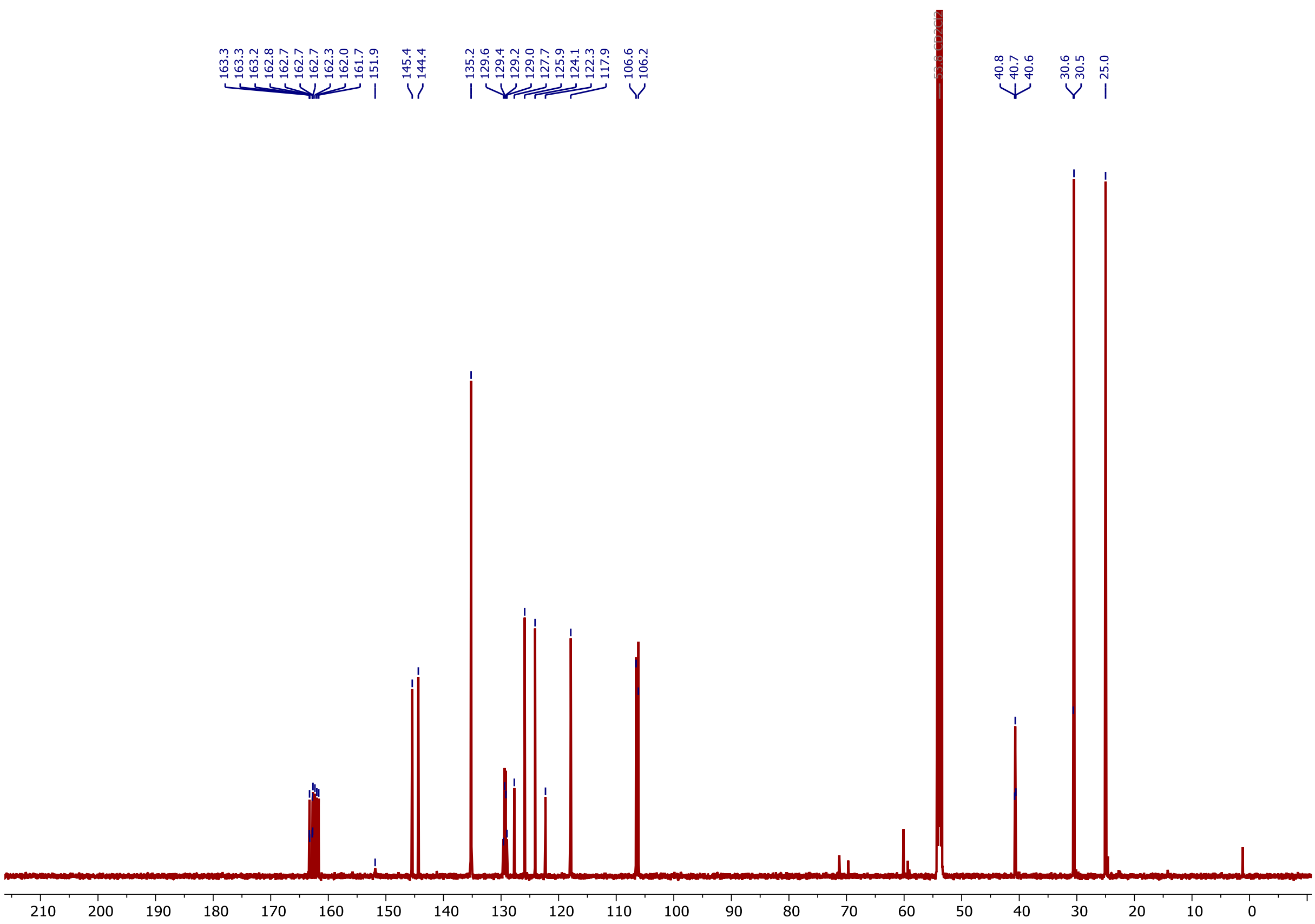

Figure S 58. $^{13}C\{^{1}H\}$*-NMR spectrum (23°C, 150 MHz) of* ***3-tBuNC*** *in DCM-*d_2.

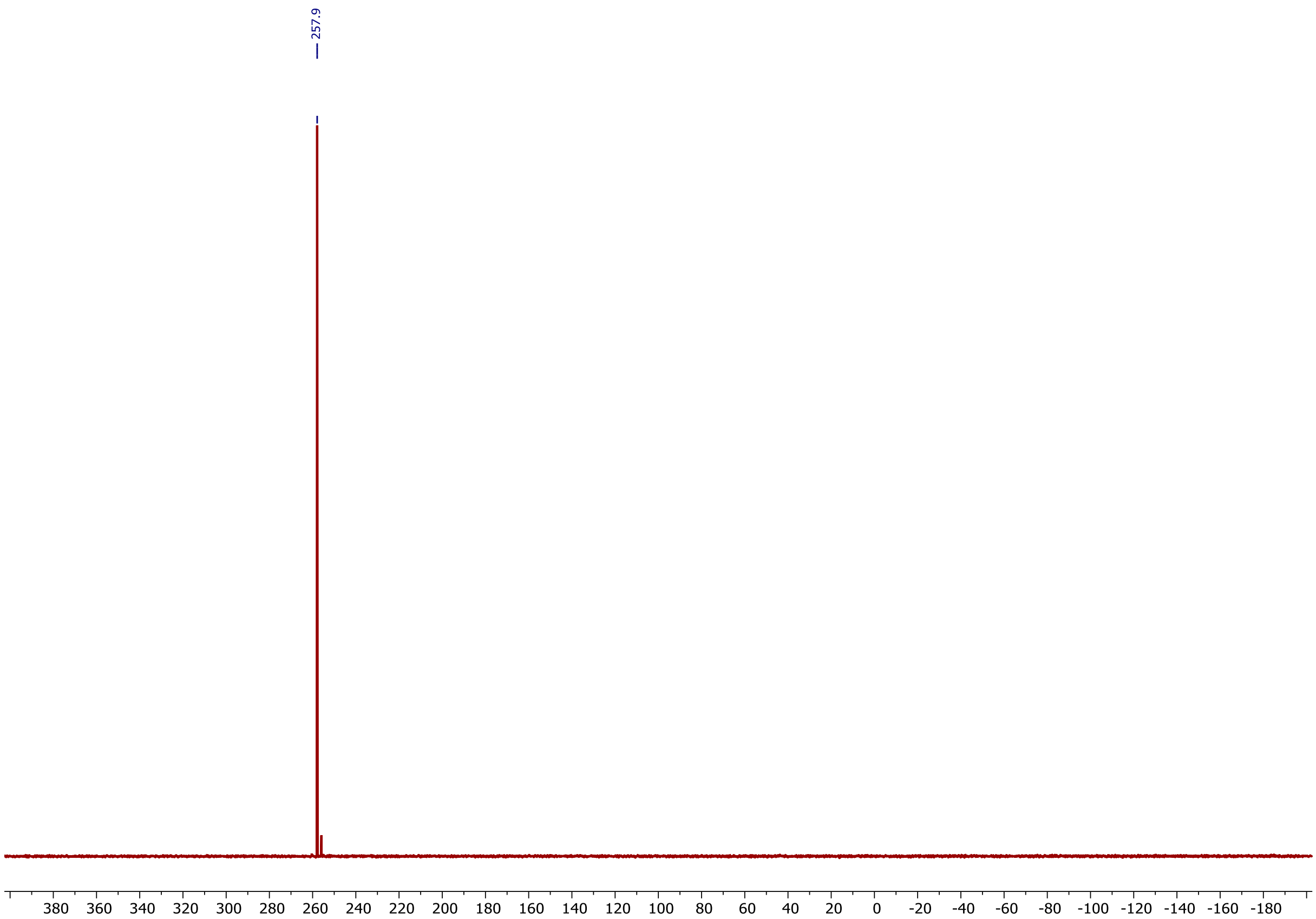

Figure S 59. $^{31}P\{^{1}H\}$*-NMR spectrum (23°C, 240 MHz) of* ***3-tBuNC*** *in DCM-*d_2.

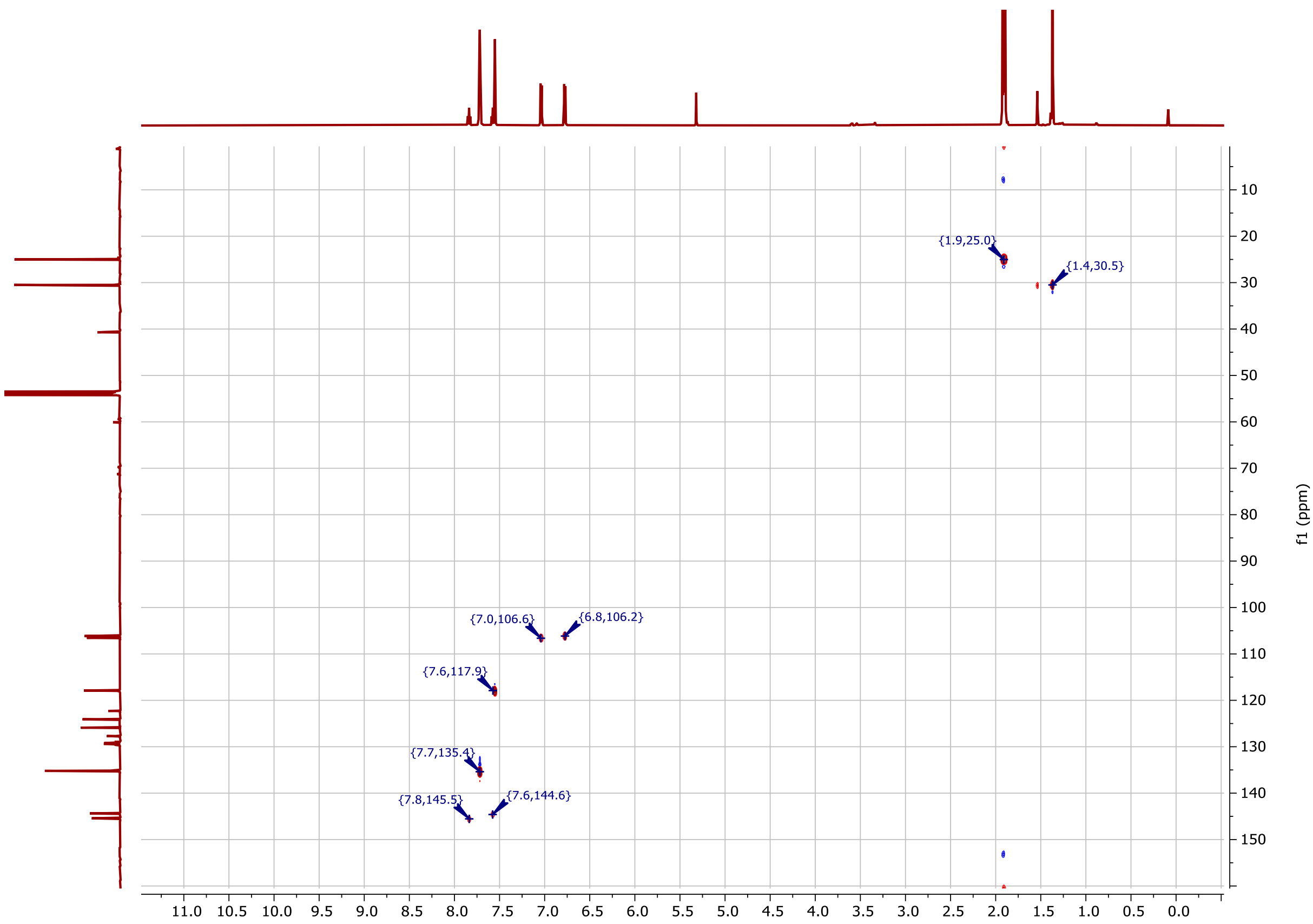

*Figure S 60. $^{1}H/^{13}C$-HSQC spectrum (23°C) of **3-tBuNC** in DCM-d_2.*

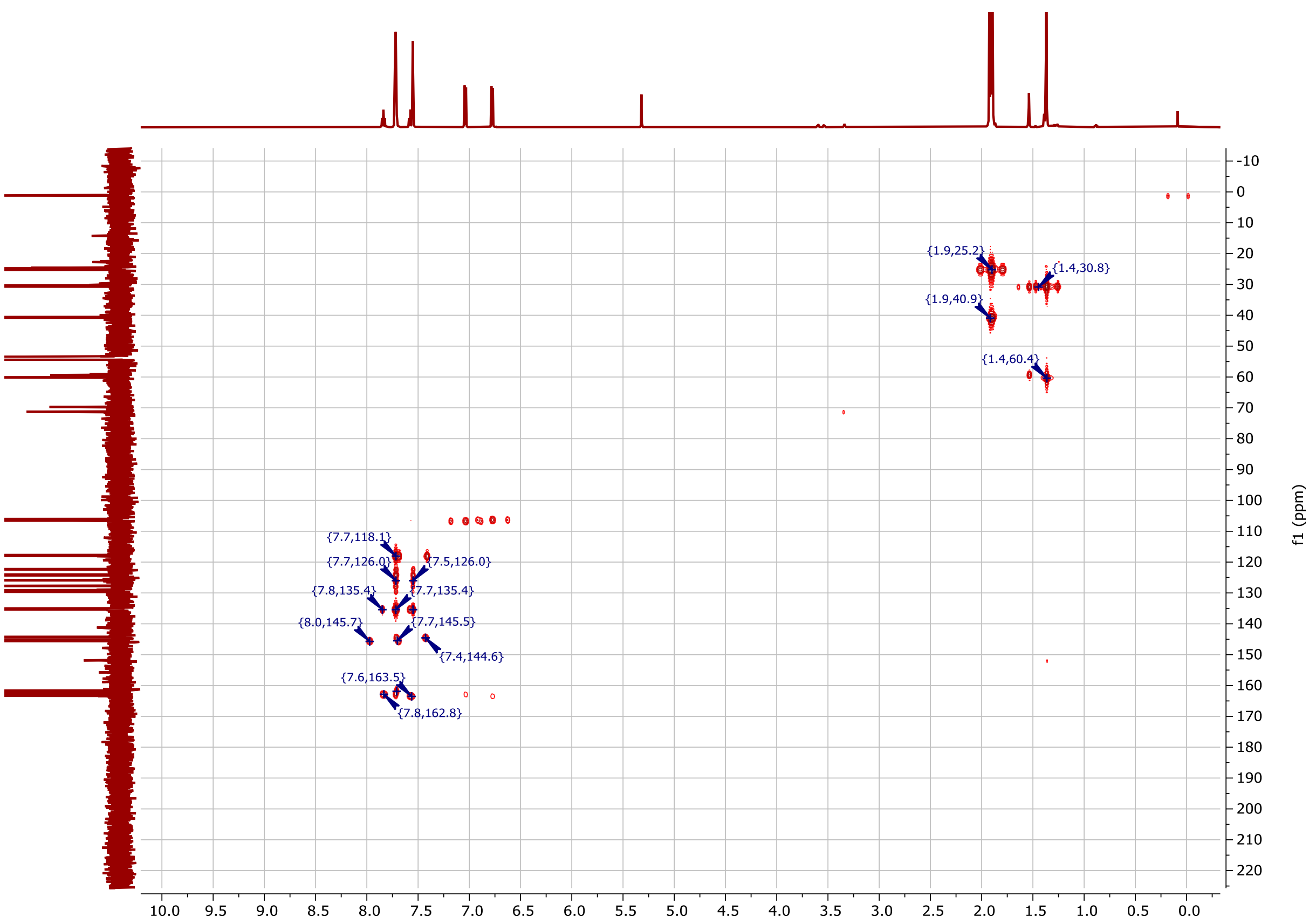

*Figure S 61. $^{1}H/^{13}C$-HMBC spectrum (23°C) of **3-tBuNC** in DCM-d_2.*

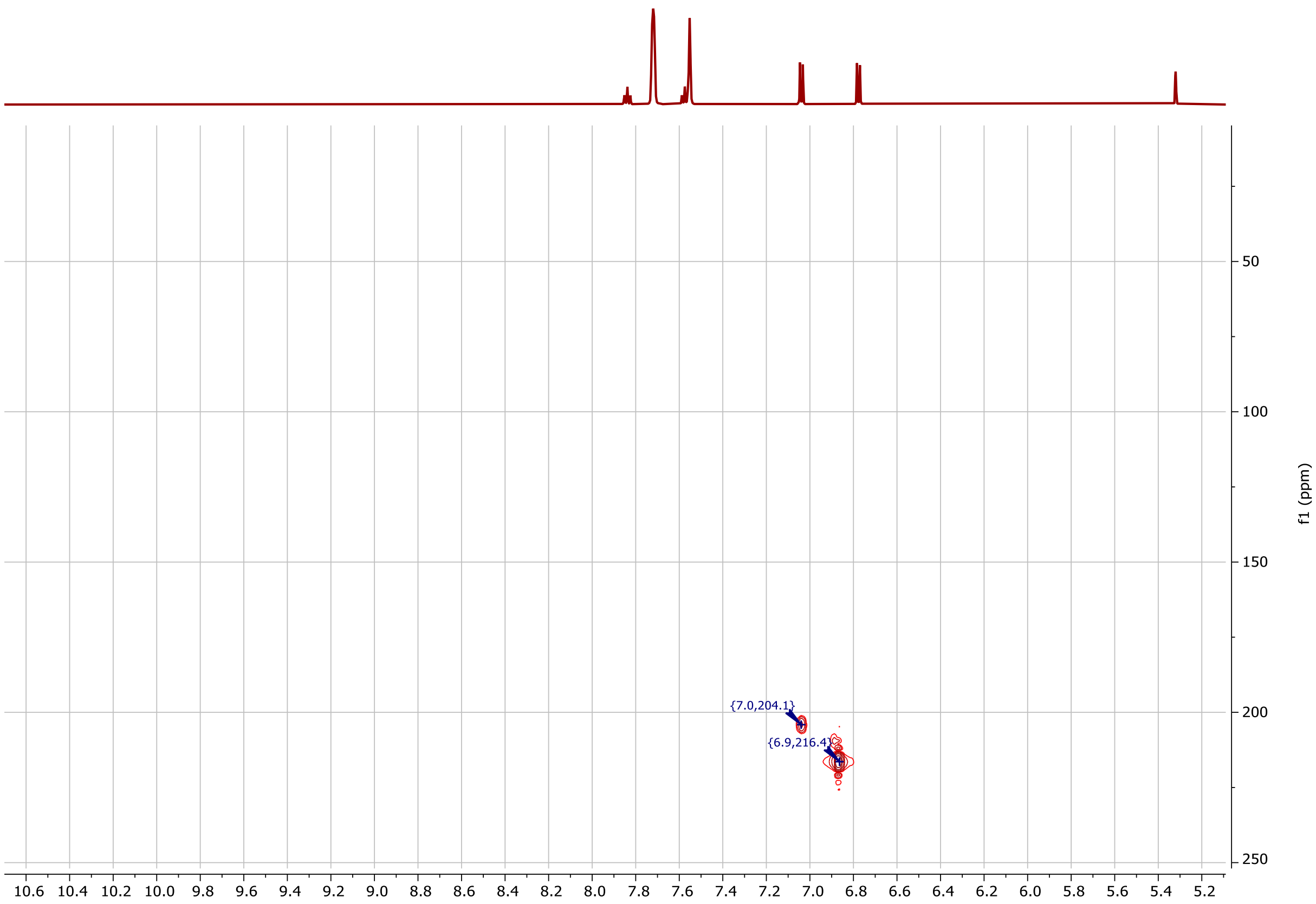

Figure S 62. ^{1}H/^{15}N-HMBC spectrum (23°C) of **3-tBuNC** in DCM-d_2.

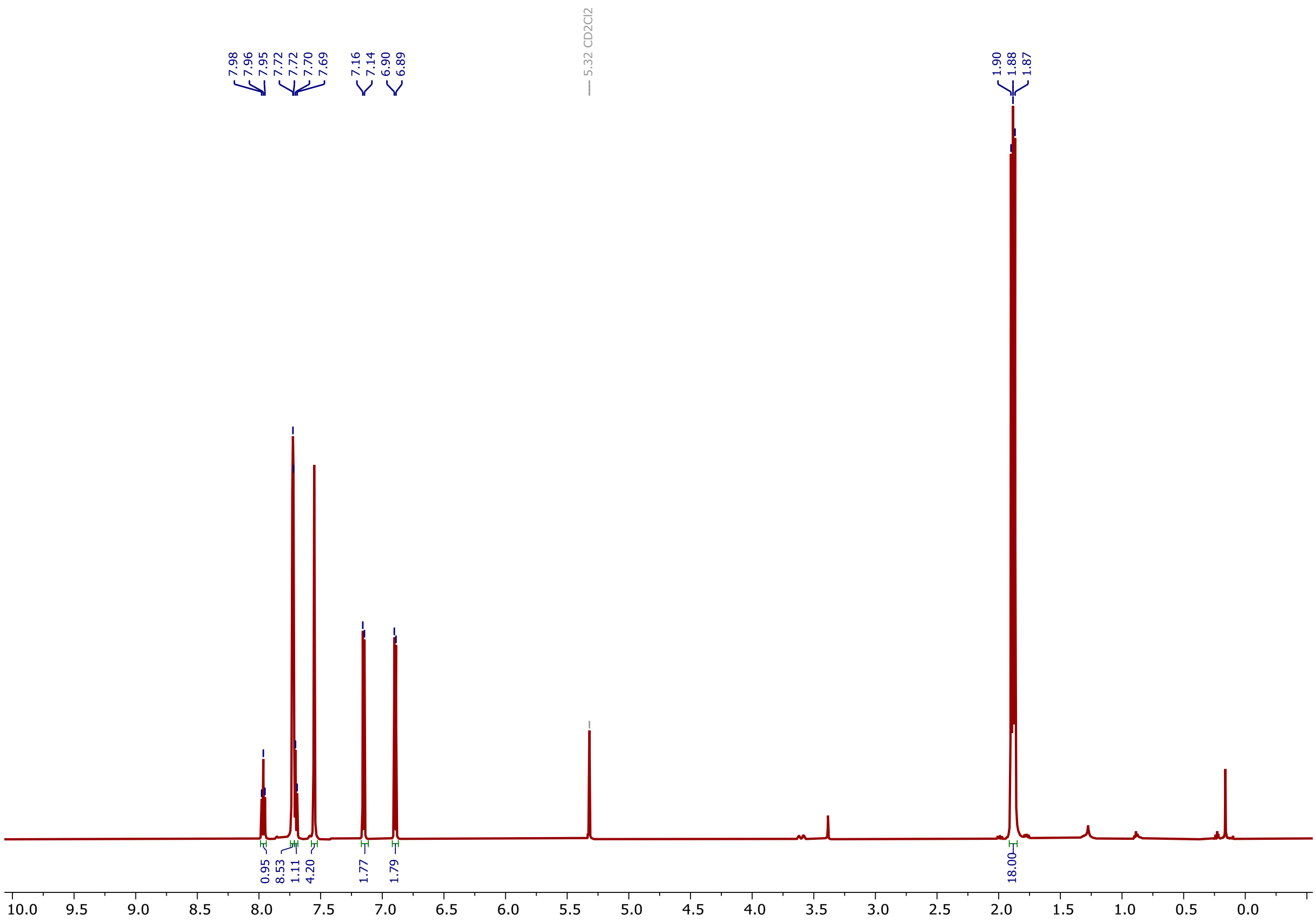

Figure S 63. ^{1}H-NMR spectrum (23°C, 600 MHz) of **3-CO** in DCM-d_2.

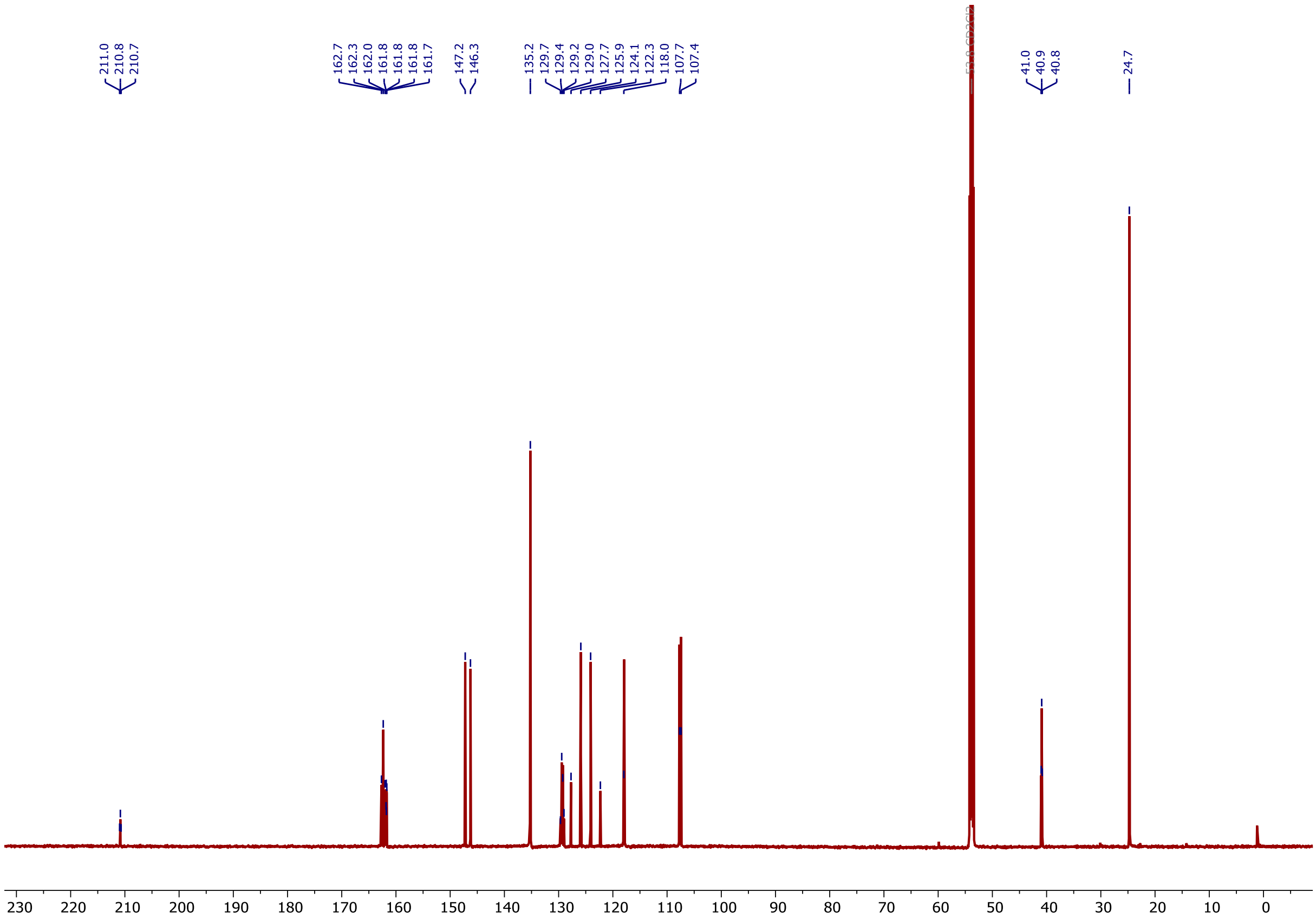

*Figure S 64. $^{13}C\{^{1}H\}$-NMR spectrum (23°C, 150 MHz) of **3-CO** in DCM-d_2.*

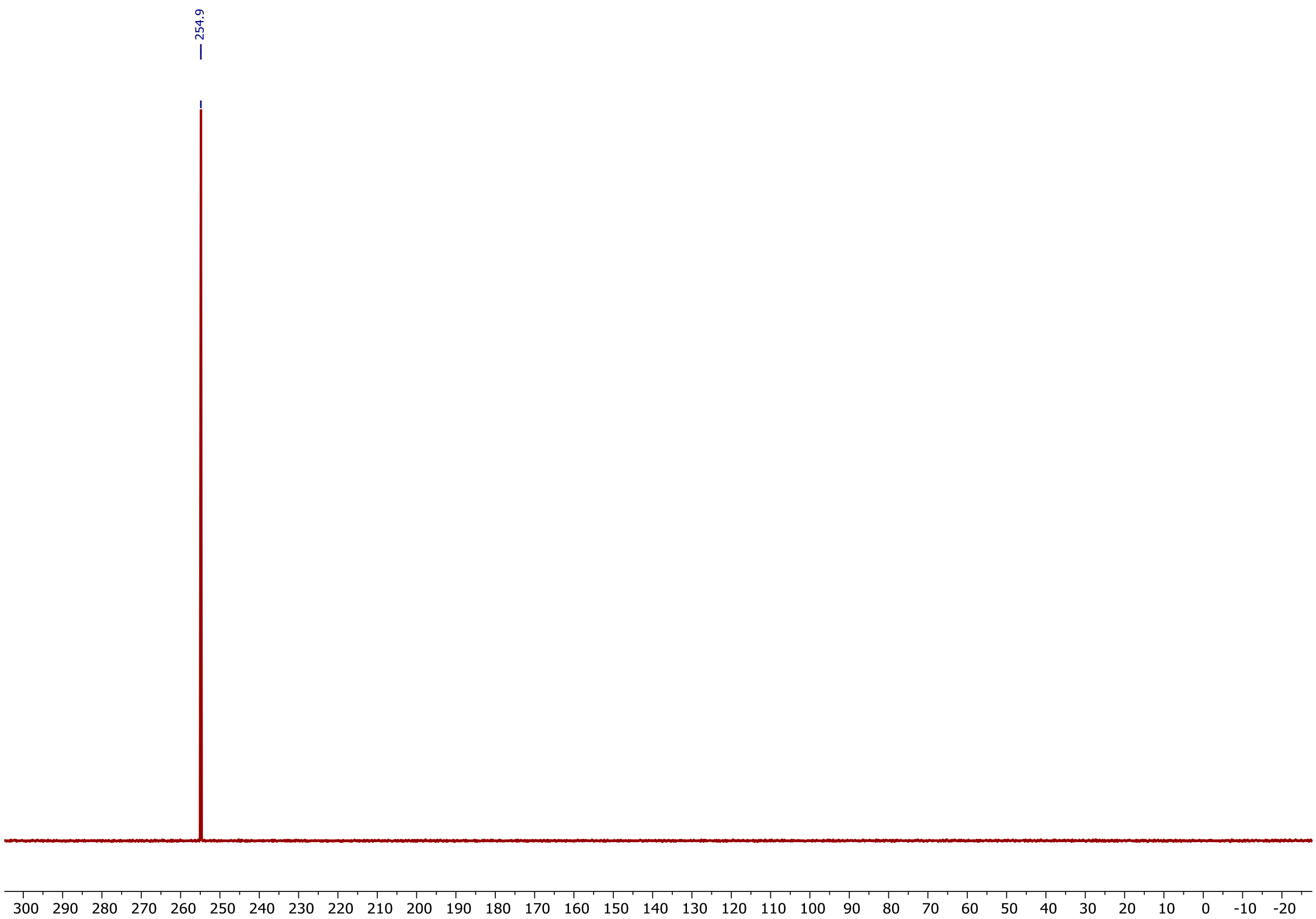

*Figure S 65. $^{31}P\{^{1}H\}$-NMR spectrum (23°C, 240 MHz) of **3-CO** in DCM-d_2.*

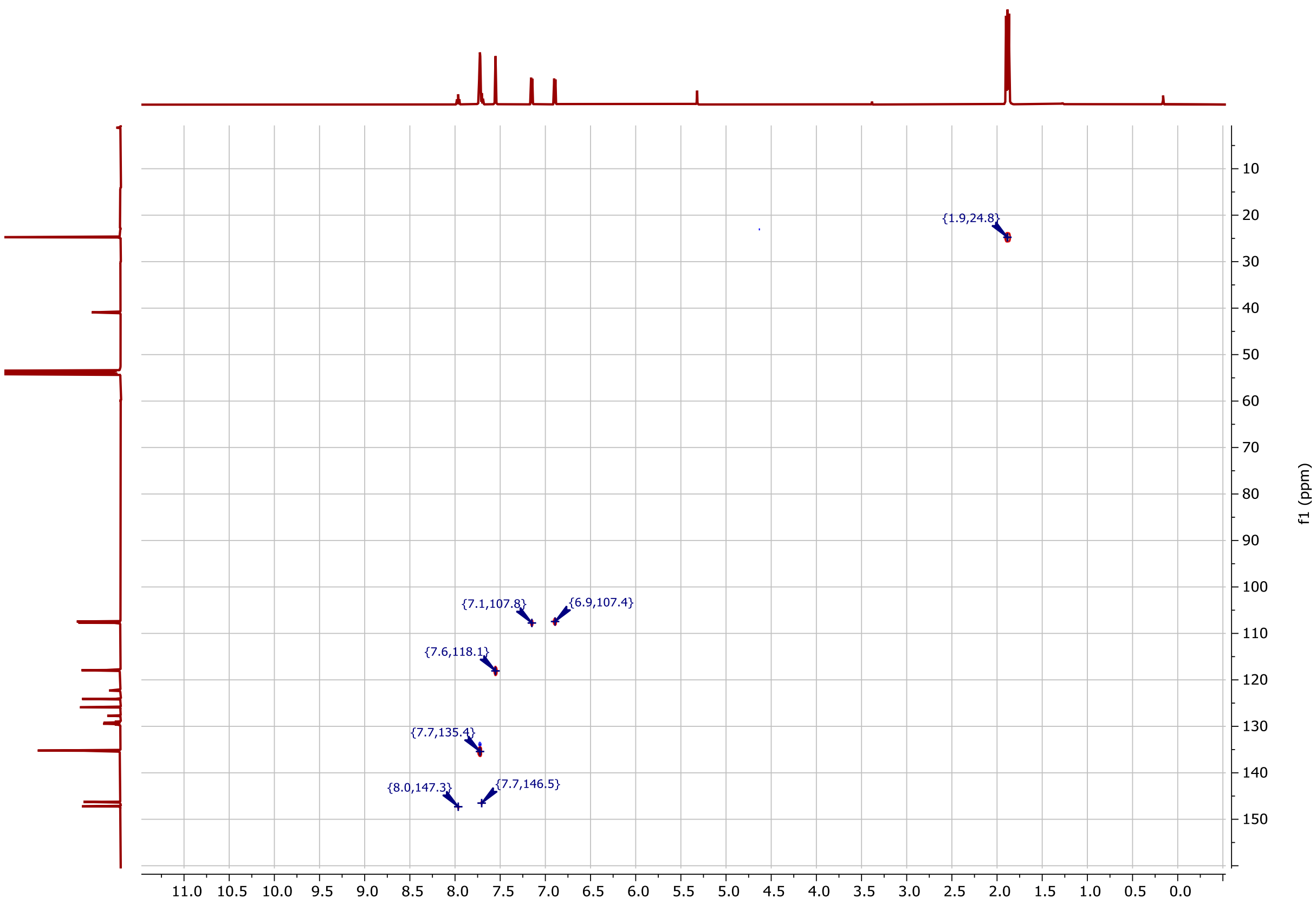

Figure S 66. $^{1}H/^{13}C$*-HMBC spectrum (23°C) of* ***3-CO*** *in DCM-d*$_2$*.*

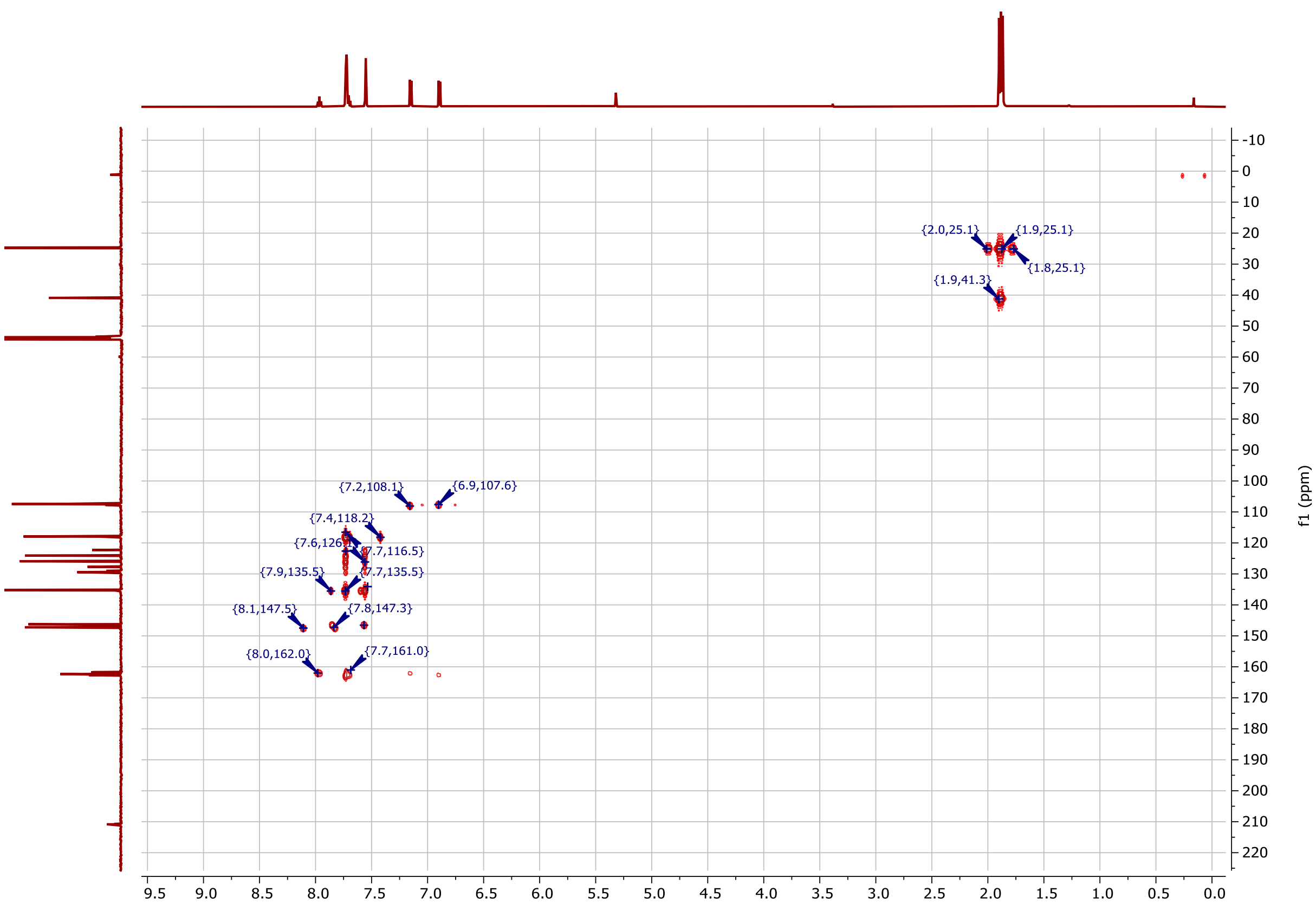

Figure S 67. $^{1}H/^{13}C$*-HMBC spectrum (23°C) of* ***3-CO*** *in DCM-d*$_2$*.*

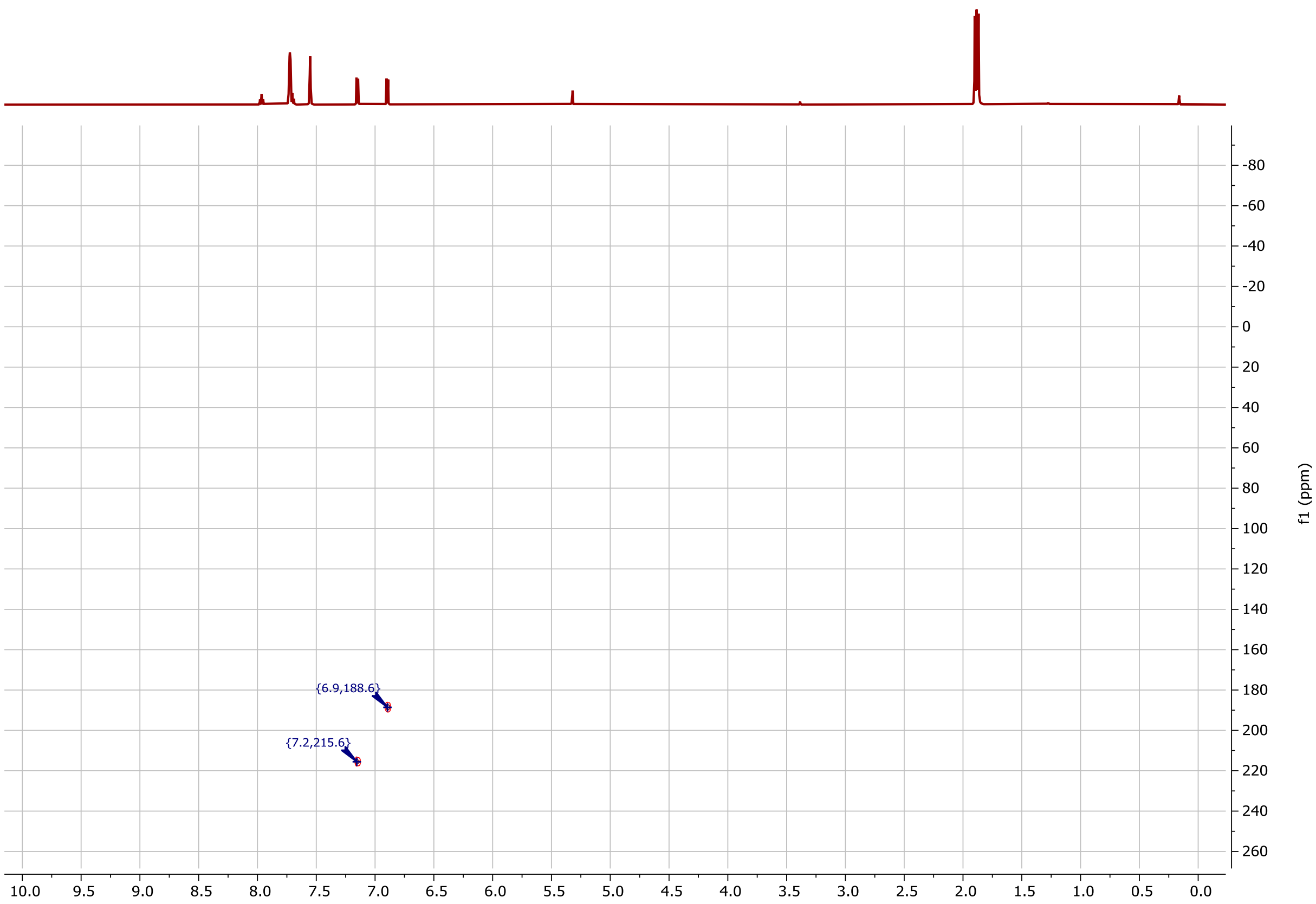

Figure S 68. *^{1}H/^{15}N-HMBC spectrum (23°C) of* ***3-CO*** *in DCM-d_2.*

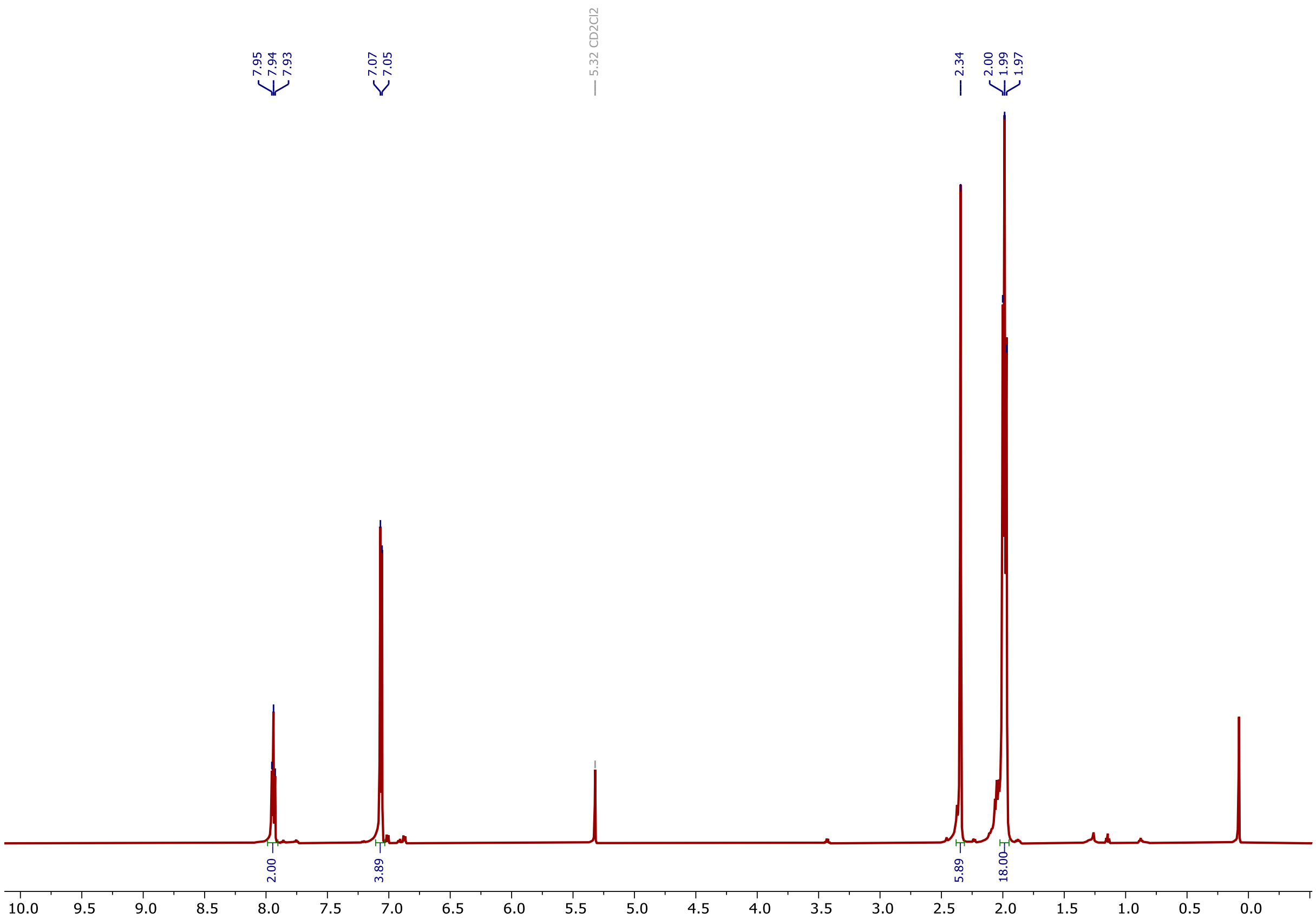

Figure S 69. *^{1}H-NMR spectrum (23°C, 600 MHz) of* ***4*** *in DCM-d_2.*

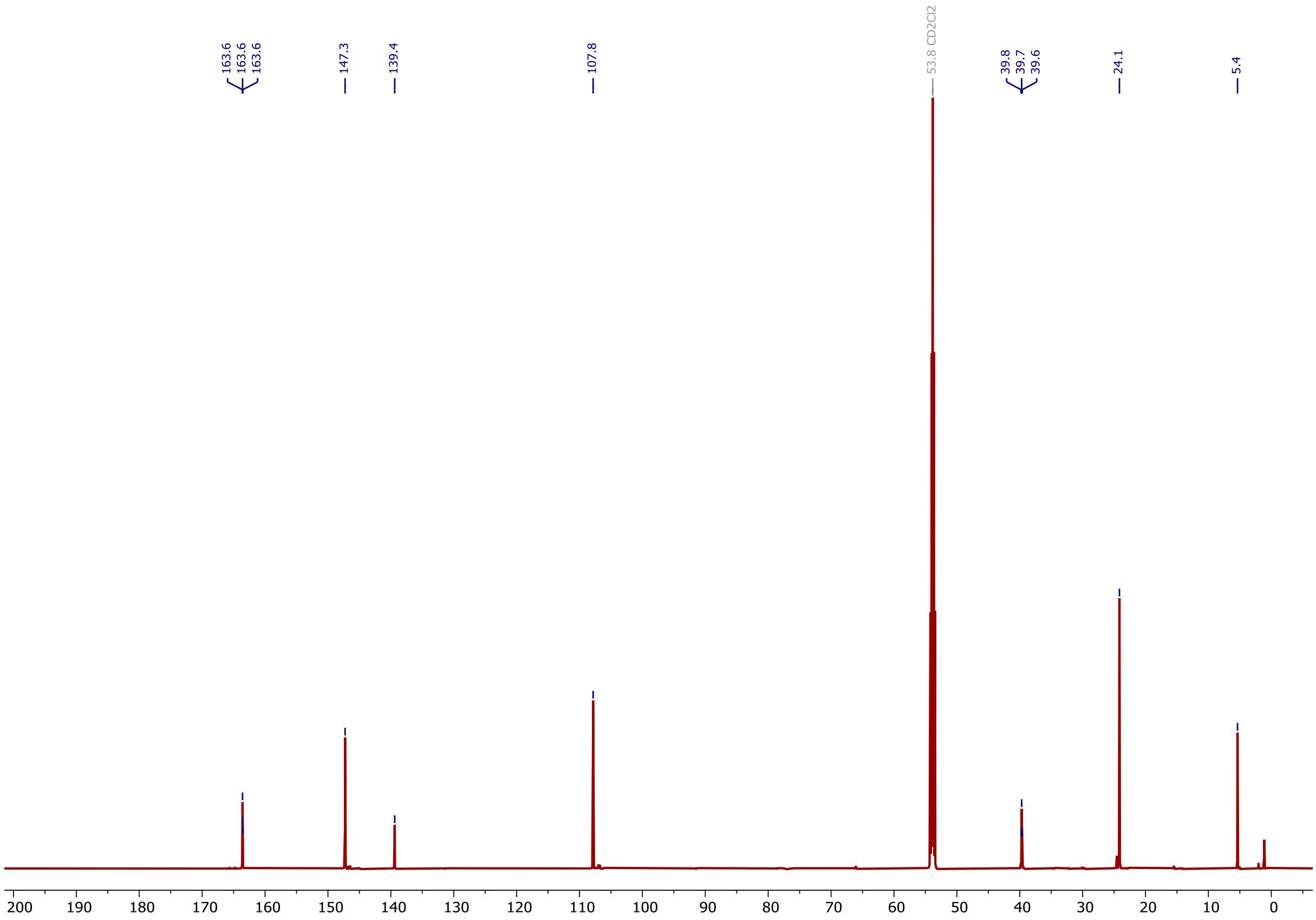

*Figure S 70. $^{13}C\{^{1}H\}$-NMR spectrum (23°C, 150 MHz) of **4** in DCM-d_2.*

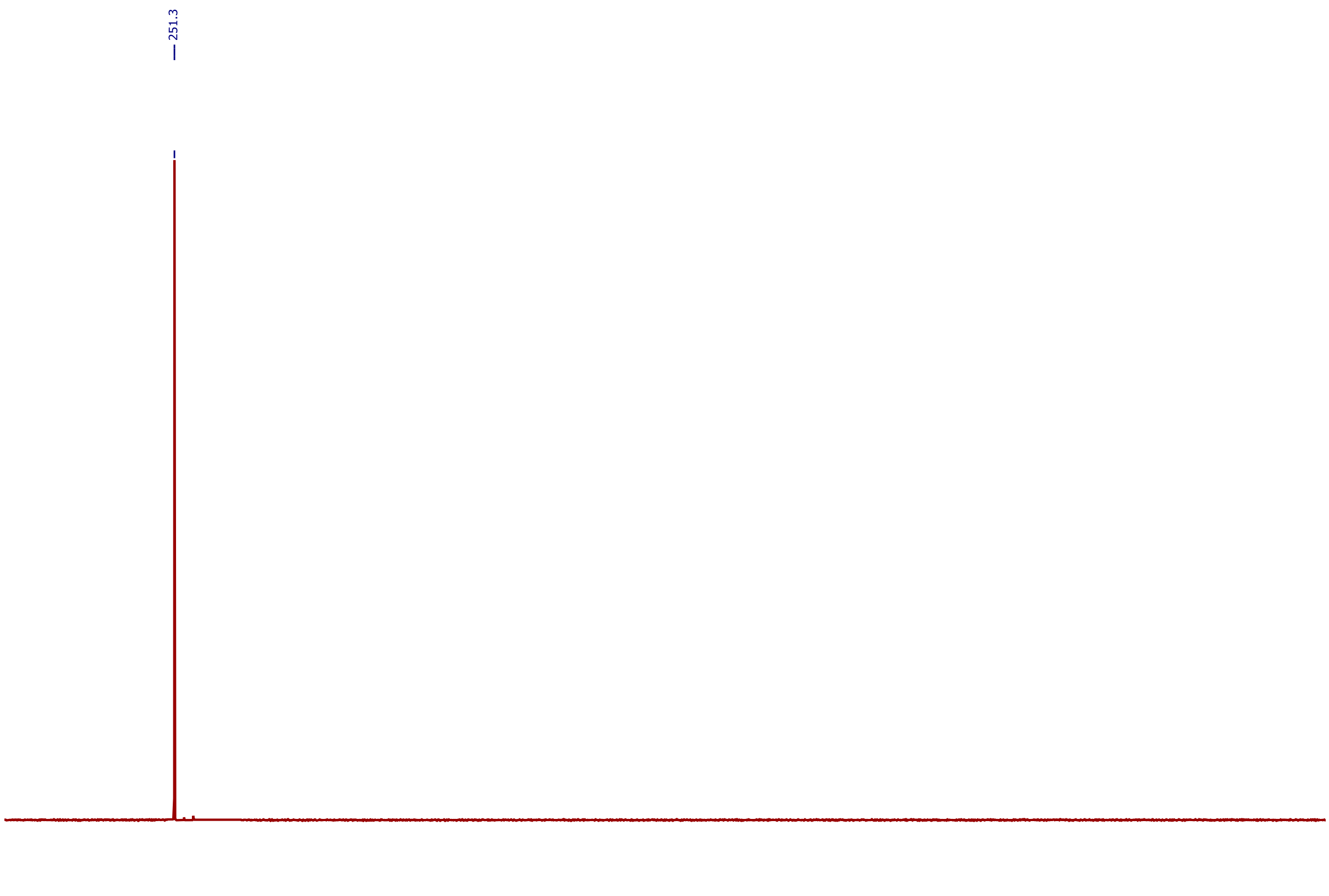

*Figure S 71. $^{31}P\{^{1}H\}$-NMR spectrum (23°C, 150 MHz) of **4** in DCM-d_2.*

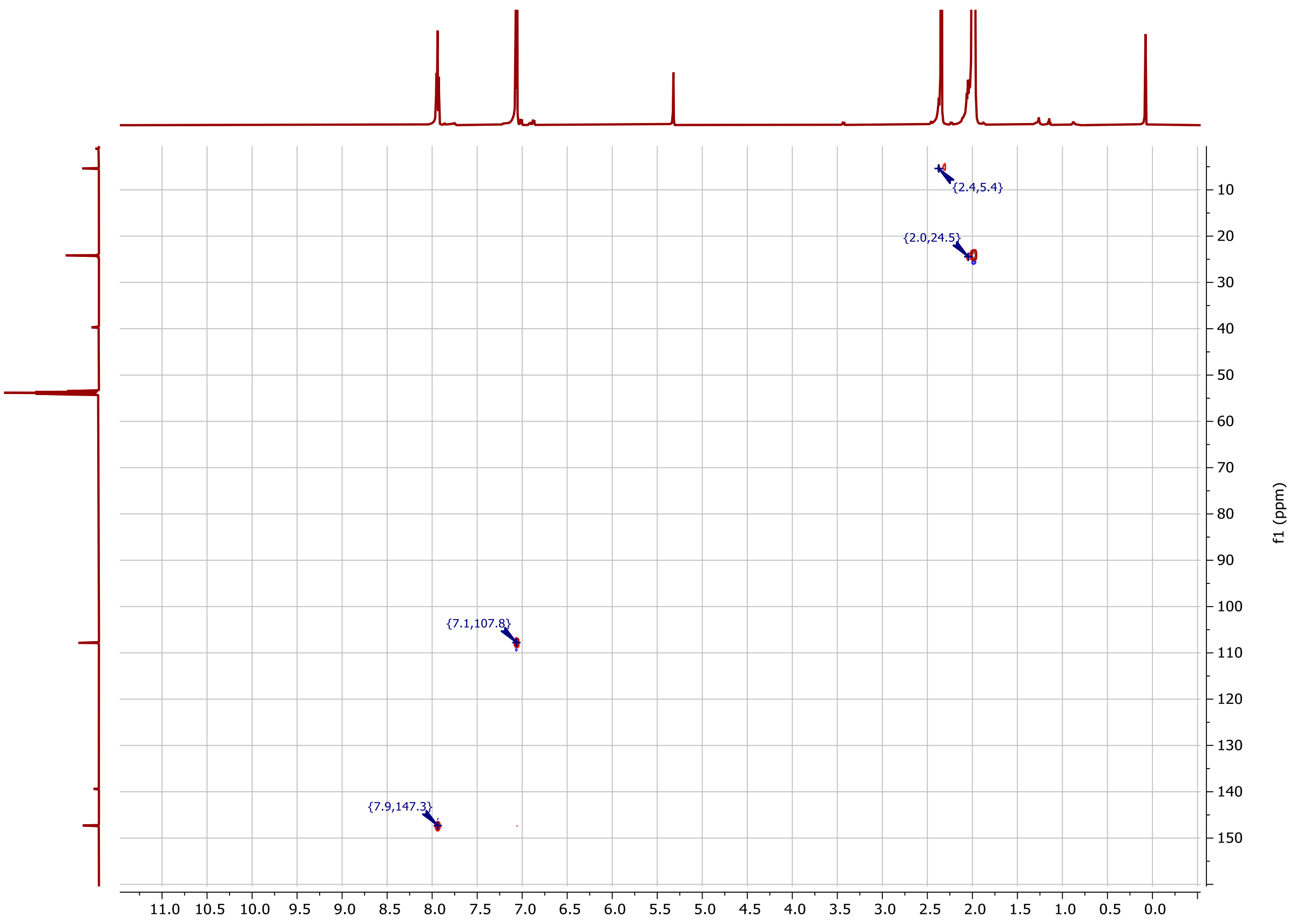

Figure S 72. *^{1}H/^{13}C-HSQC spectrum (23°C) of **4** in DCM-d_2.*

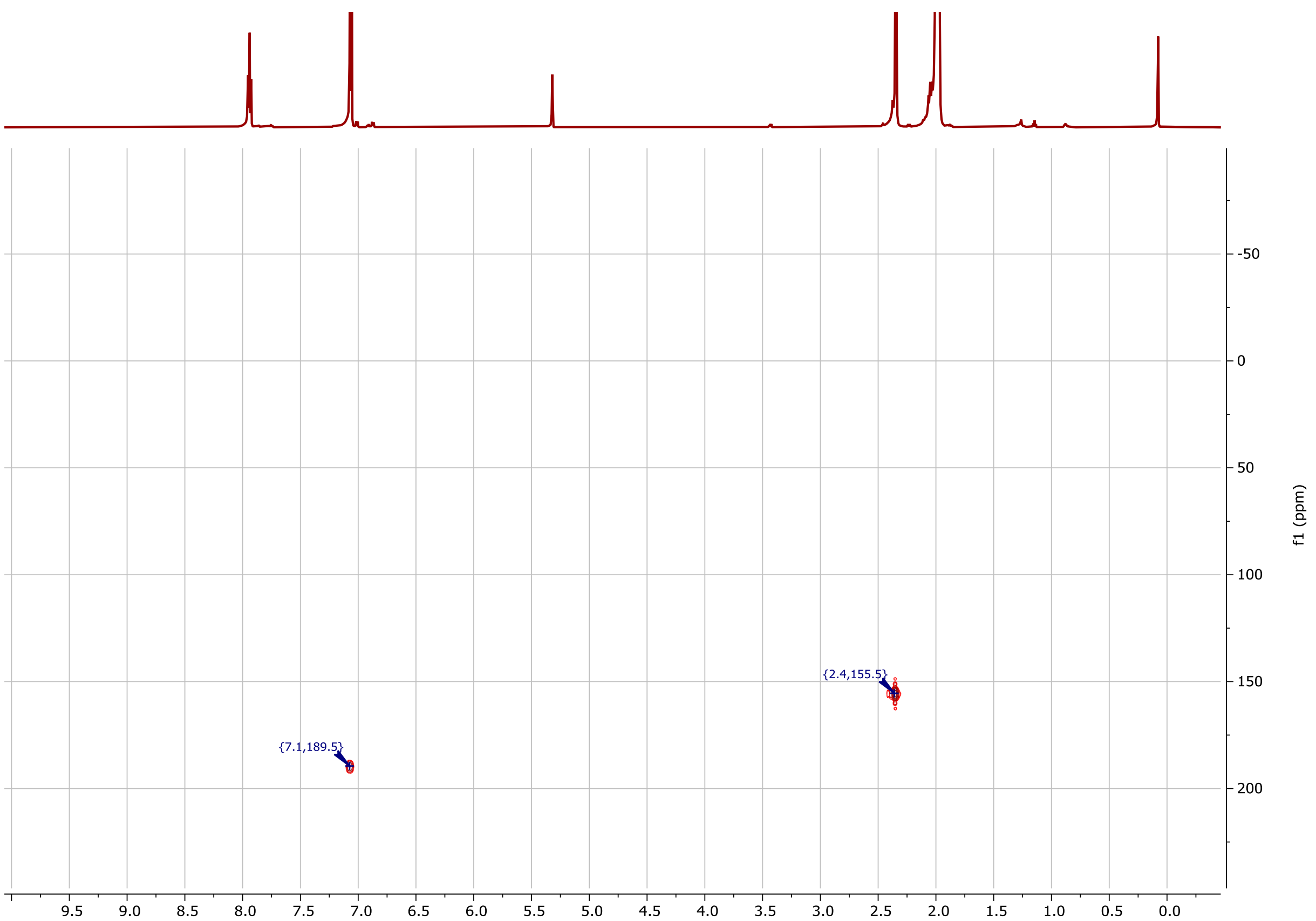

Figure S 73. *^{1}H/^{15}N-HMBC spectrum (23°C) of **4** in DCM-d_2.*

6 References

(1) Fuchigami, K.; Watson, M.B.; Tran, G.N.; Rath, N.P.; Mirica, L.M. Synthesis and Reactivity of $(N_2P_2)Ni$ Complexes Stabilized by a Diphosphonite Pyridinophane Ligand. *Organometallics* **2021**, *40*, 2283-2289, DOI: 10.1021/acs.organomet.1c00003.

(2) Evans, D. F. The Determination of the Paramagnetic Susceptibility of Substances in Solution by Nuclear Magnetic Resonance. *J. Chem. Soc.* **1959**, 2003–2005, DOI: 10.1039/jr9590002003.

(3) Staroverov, V.N.; Scuseria, G.E.; Tao, J.; Perdew, J.P. Comparative assessment of a new nonempirical density functional: Molecules and hydrogen-bonded complexes. *J. Chem. Phys.* **2003**, *119*, 12129–12137, DOI: 10.1063/1.1626543.

(4) Pecak, J.; Talmazan, R.A.; Svatunek, D.; Kirchner, K.; Podewitz, M. Is Mn(I) More Promising Than Fe(II)─A Comparison of Mn vs Fe Complexes for Olefin Metathesis. *Organometallics* **2024**, *43*, 457-466, DOI: 10.1021/acs.organomet.3c00398.

(5) Weigend, F.; Ahlrichs, R. Balanced basis sets of split valence, triple zeta valence and quadruple zeta valence quality for H to Rn: Design and assessment of accuracy. *Phys. Chem. Chem. Phys.* **2005**, *7*, 3297-3305, DOI: 10.1039/B508541A.

(6) Caldeweyher, E.; Ehlert, S.; Hansen, A.; Neugebauer, H.; Spicher, S.; Bannwarth, C.; Grimme, S. A generally applicable atomic-charge dependent London dispersion correction. *J. Chem. Phys.* **2019**, *150*, 154122- 19, DOI: 10.1063/1.5090222.

(7) Hirshfeld, F. L. Bonded-atom fragments for describing molecular charge densities. *Theor. Chim. Acta* **1977**, *44*, 129-138, DOI: 10.1007/BF00549096.

(8) Neese, F. Software Update: The ORCA Program System—Version 6.0 *Comput. Mol. Sci.* **2025**, e70019, DOI: 10.1002/wcms.70019.

(9) Yanai, T.; Tew, D.P.; Handy, N. C. A new hybrid exchange–correlation functional using the Coulomb-attenuating method (CAM-B3LYP). *Chem. Phys. Lett.* **2004**, *393*, 51-57, DOI: 10.1016/j.cplett.2004.06.011.

(10) Jacquemin, D., Adamo, C. Computational Molecular Electronic Spectroscopy with TD-DFT. In: Ferré, N., Filatov, M., Huix-Rotllant, M. (eds) Density-Functional Methods for Excited States. Topics in Current Chemistry, **2015**, *368*. Springer, Cham. DOI: 10.1007/128_2015_638.

(11) X-Area 1.31.192.0, LANA 2.7.12 (STOE & Cie GmbH, Darmstadt, Germany, 2023).

(12) Sheldrick, G.M. SHELXT-Integrated Space-Group and Crystal-Structure Determination. *Acta Crystallogr.* **2015**, *A71*, 3–8, DOI: 10.1107/S2053273314026370.

(13) Sheldrick, G.M. Crystal Structure Refinement with SHELXL. *Acta Crystallogr.* **2015**, *C71*, 3–8, DOI: 10.1107/S2053229614024218.

(14) Macrae, C.F; Edgington, P.R.; McCabe, P.; Pidcock, E.; Shields, G.P.; Taylor, R.; Towler, M.; van de Streek, J. Mercury: Visualization and Analysis of Crystal Structures. *J. Appl. Cryst.* **2006**, *39*, 453–457, DOI: 10.1107/S002188980600731X.
